# Transition-metal-free α-arylation of oxindoles *vi*a visible-light-promoted electron transfer†
†Electronic supplementary information (ESI) available: Full experimental procedures and characterisation for all compounds. See DOI: 10.1039/c8sc05170d


**DOI:** 10.1039/c8sc05170d

**Published:** 2019-01-22

**Authors:** Kangjiang Liang, Na Li, Yang Zhang, Tao Li, Chengfeng Xia

**Affiliations:** a Key Laboratory of Medicinal Chemistry for Natural Resources (Ministry of Education and Yunnan Province) , School of Chemical Science and Technology , Yunnan University , 2 North Cuihu Road , Kunming 650091 , China . Email: xiacf@ynu.edu.cn

## Abstract

An operationally simple photochemical strategy for the direct arylation of oxindoles with (hetero)aryl halides in the absence of both transition metals and photoredox catalysts has been developed. The reaction provides an efficient way to construct various 3-aryloxindole building blocks of pharmaceutical interest at ambient temperature by using household compact fluorescent light (CFL) bulbs as the light source. Preliminarily, mechanistic studies revealed that the intermolecular electron transfer relied on the formation of photon-absorbing electron–donor–acceptor (EDA) complexes between electron-rich oxindole enolates and electron-deficient (hetero)aryl halides, and a radical chain mechanism was operative.

## Abstract 2

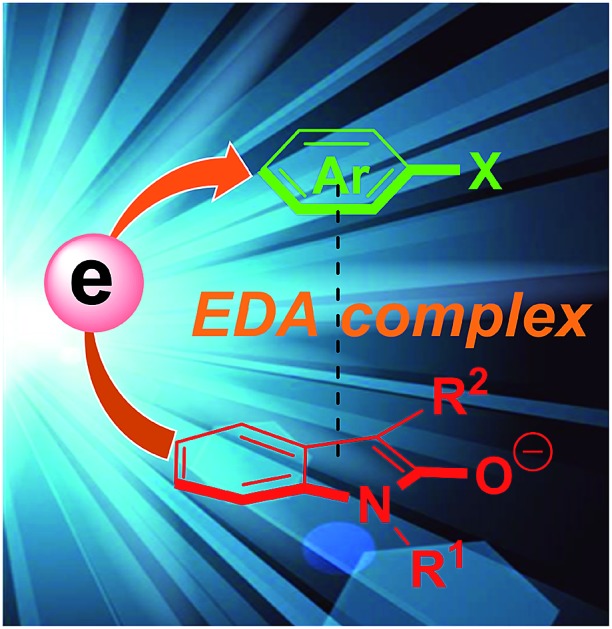
An operationally simple photochemical strategy for the direct arylation of oxindoles with (hetero)aryl halides in the absence of both transition metals and photoredox catalysts has been developed.

## Introduction

**Fig. 1:**
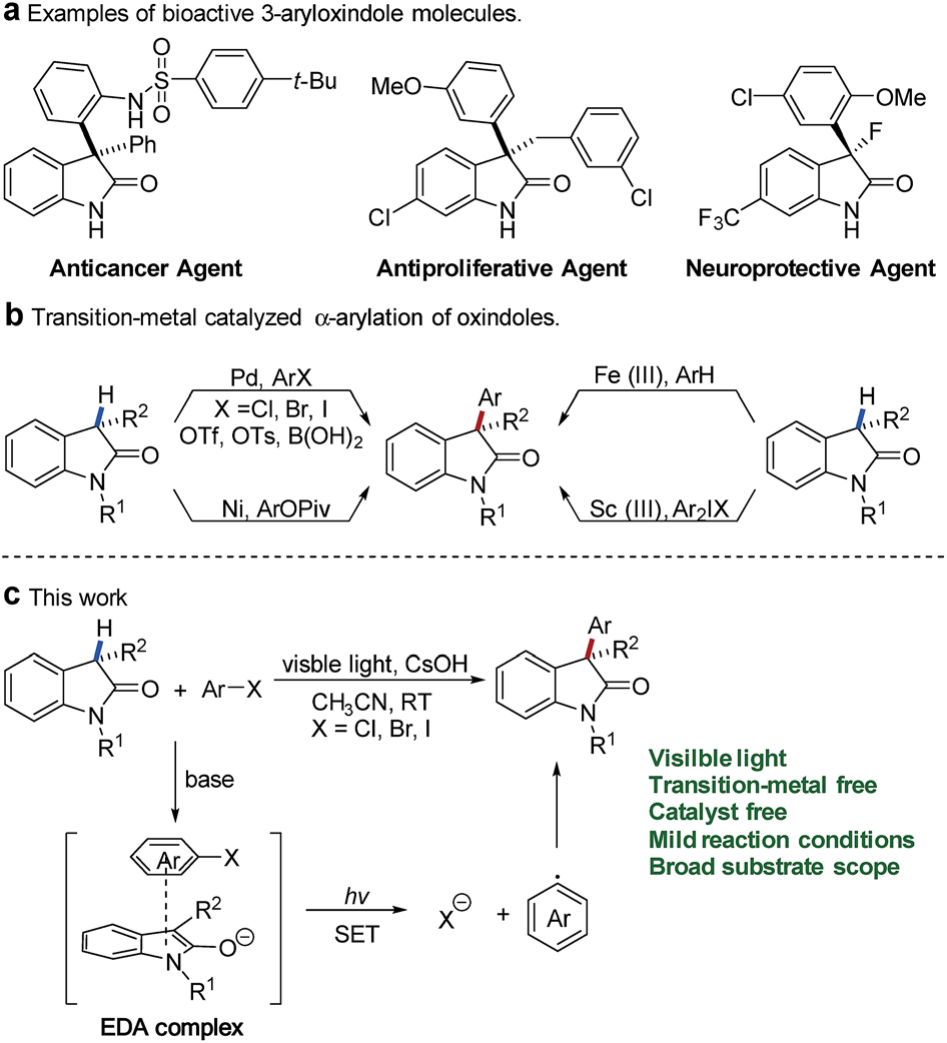
(a) Pharmaceutically active molecules with a 3-aryloxindole moiety. (b) Transition-metal-catalyzed α-arylation of oxindole. (c) Visible-light-promoted arylation of oxindole with an EDA complex.

Oxindoles constitute an essential motif in a wide range of biologically active natural products and a series of pharmaceutically active molecules.^1^ Among them, 3-aryloxindoles are valuable architectures in drug development and display a broad spectrum of biological activities, for instance, as an anticancer agent,^2^ antiproliferative agent,^3^ and neuroprotective agent^4^ (Fig. 1a). In this context, methods for the direct α-arylation of oxindoles hold particular promise because of their ability to construct bioactive molecules of pharmaceutical interest.

During the past two decades, the synthesis of 3-aryloxindoles mainly focused on the transition-metal-catalyzed cross-coupling of oxindole cores with various aromatic compounds (Fig. 1b). Significant efforts have been directed towards the development of palladium-catalyzed cross-coupling reactions of oxindoles with aryl halides,^5^ aryl triflates,^6^ aryl tosylates,^7^ or arylboronic acids.^8^ Alternatively, Itami and co-workers provided a nickel-catalyzed method for 3-aryloxindole formation by α-arylation of oxindoles with aryl pivalates.^9^ In 2015, Li and coworkers described an Fe(iii)-catalyzed cross-dehydrogenative arylation (CDA) between oxindoles and electron-rich arenes under an air atmosphere.^10^ Additionally, Feng and co-workers reported a scandium(iii)-catalyzed α-arylation of oxindoles with diaryliodonium salts.^11^ Although significant advances in transition-metal-catalyzed arylation have been made, tremendous challenges still remain with respect to expensive metal catalysts and specific or air-sensitive ligands.

In contrast, transition-metal-free arylations were only limited in the S_N_Ar reaction of oxindoles with electron-deficient nitroaryl fluorides^12^ or oxidative nucleophilic addition of oxindoles to nitroarenes.^13^ In addition, Srihari and coworkers reported a multiple aryne insertion approach to construct 3,3-diarylated oxindoles.^14^ Even though the above achievements have been made, the direct α-arylation of oxindoles with inactive aryl halides under transition-metal-free conditions remains a challenge.

Recently, visible-light-promoted photochemical transformations by intermolecular electron transfer have received great attention^15^ and (hetero)aryl halides have also been disclosed as effective electron acceptors in the thiolate–(hetero)aryl halide EDA complex.^16^ In addition, methods for photoinduced S_RN_1 arylation of ketone enolates by excitation of EDA complexes between ketone enolates and aryl iodides were also reported.^17^ Oxindole enolates are a strong electron donor and have been widely used in SET oxidation reactions.^18^ Inspired by these studies, we questioned whether electron-rich oxindole enolates could act as donors for the formation of EDA complexes with (hetero)aryl halides to realize the direct α-arylation of oxindoles under visible-light irradiation. In our design, we envisaged that activation of (hetero)aryl halides might be completed by visible-light-promoted intermolecular electron transfer within the oxindole enolate–(hetero)aryl halide EDA complex (Fig. 1c). The resulting radical anion of (hetero)aryl halide would be able to release a halogen anion to form an electrophilic aryl radical (Ar˙),^19^ which was further trapped by electron-rich oxindole enolate to afford the desired product.

## Results and discussion

### Optimization of reaction conditions

With this design in mind, our initial investigations focused on the cross-coupling between iodobenzene **1a** and the oxindole derivative **2a** (Table 1). To our delight, when using a household 23 W compact fluorescent light (CFL) bulb to irradiate a DMSO solution of iodobenzene and **2a** in the presence of CsOH as the base, we observed that the desired α-arylation product **3a** was obtained in a moderate yield of 52% at room temperature (entry 1). Conduction of the experiments under illumination with a 30 W white LED resulted in a slight reduction of the yield (entry 2). Among the solvents screened (entries 3–6), acetonitrile was most efficient for this reaction, and the desired product was obtained in 85% yield (entry 4). Evaluating various alkoxide/hydroxide bases indicated that bases with larger radius alkali metal cations, such as Cs^+^ or K^+^, provided the arylation product in moderate to good yields (entries 4, 7, and 10), whereas bases with Na^+^ or Li^+^ gave decreased yields or failed to afford any products (entries 8, 9, 11, and 12).

**Table 1:** Optimization studies

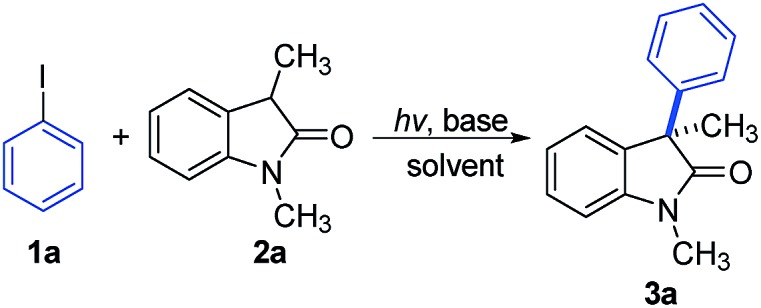				
Entry^*a*^	*hv*	Base	Solvent	Yield^*b*^ (%)
1	23 W CFL	CsOH^*c*^	DMSO	52
2	30 W white LED	CsOH	DMSO	46
3	23 W CFL	CsOH	DMF	38
4	23 W CFL	CsOH	CH_3_CN	85
5	23 W CFL	CsOH	THF	19
6	23 W CFL	CsOH	DCM	0
7	23 W CFL	*t*BuOK	CH_3_CN	55
8	23 W CFL	*t*BuONa	CH_3_CN	21
9	23 W CFL	*t*BuOLi	CH_3_CN	0
10	23 W CFL	KOH	CH_3_CN	62
11	23 W CFL	NaOH	CH_3_CN	15
12	23 W CFL	LiOH	CH_3_CN	0

Entry^*a*^	Change from the best conditions (entry 4)	Yield^*b*^ (%)
13	No base	0
14	No light	0
15	No light and heating to 80 °C	0
16	Bromobenzene instead of iodobenzene	51
17	Chlorobenzene instead of iodobenzene	34

^*a*^Reaction conditions: 1 equiv. aryl halide, 2 equiv. oxindole, and 3 equiv. base [0.1 M]; all solvents were rigorously degassed by freeze/pump/thaw.

^*b*^Isolated yields obtained by chromatography.

^*c*^CsOH·H_2_O is abbreviated as CsOH.

**Fig. 2:**
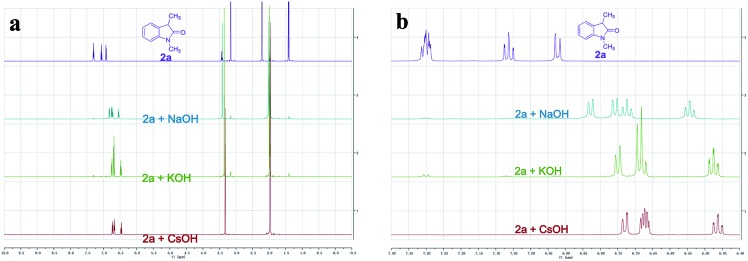
^1^H NMR spectra of **2a** with different MOHs (M = Na^+^, K^+^, or Cs^+^) in CD_3_CN. (a) Full ^1^H NMR spectra. (b) Selected aromatic region of the ^1^H NMR spectral window between 7.40 and 6.40 ppm.

To better understand whether different alkali metal cations affecting reactivity was due to either the deprotonation step or a secondary effect in the electron transfer process,^16^ we recorded the ^1^H NMR spectra of **2a** with different MOHs (M = Na^+^, K^+^, or Cs^+^) in CD_3_CN. Complete enolate formation of **2a** was observed after treatment with 1.5 equiv. of MOH (M = Na^+^, K^+^, or Cs^+^), which demonstrated that all the MOHs could efficiently deprotonate the oxindole (Fig. 2a). Moreover, significant upfield peak shifting of aromatic hydrogens was observed with the magnitude following the order Cs^+^ solution > K^+^ solution > Na^+^ solution (Fig. 2b). These results provided evidence that different alkali metal cations affecting reactivity might be due to a secondary effect in the electron transfer process. The important role of CsOH was further highlighted by a control experiment which showed that the desired product **3a** was not formed in the absence of base (entry 13). Control reactions also revealed the photochemical nature of this transformation, as essentially no C–C cross-coupling was observed in the absence of light, even upon heating at 80 °C (entries 14 and 15). Apart from iodobenzene, bromobenzene and chlorobenzene were also successfully coupled to oxindoles, albeit with lower yields (entries 16 and 17).

### Substrate scope

With the optimum conditions in hand, the scope of the arylation reaction was explored. As illustrated in Table 2, a wide range of (hetero)aryl halides (ArX, where X = I, Br, or Cl) were successfully employed and they afforded the corresponding 3-aryloxindole products in moderate to high yields. First, aryl halides with different substitution patterns, in terms of electronic properties and substitution positions on the aromatic ring, were tested. Under the standard conditions, substrates with electron-rich, electron-neutral or electron-poor substituents positioned para to the halogen proceeded smoothly (**3b–3f**). Exploration demonstrated that aryl halides with an electron-poor substituent on the aromatic ring gave slightly higher yields than those with electron-rich or electron-neutral substituents. Meta-substituted substrates were also applicable substrates for this photoinduced arylation reaction (**3g–3i**), whereas substrates with ortho-substituents led to low yields, probably due to the steric hindrance from the substituents (**3j** and **3k**). Next, a series of polycyclic aromatic hydrocarbon halides was employed. To our pleasure, polycyclic aromatic hydrocarbons including naphthalene, phenanthrene, anthracene, and fluorene were efficient coupling partners under the optimal conditions (**3l–3o**). Finally, we investigated this arylation reaction involving various heterocycles, which are ubiquitous among pharmaceutical products. Remarkably, heterocycles such as pyridine, quinoline, isoquinoline, thiophene, thianaphthene, and benzofuran also readily participated in the reaction and provided the desired products with acceptable to good yields in this photoinduced system (**3p–3u**).

**Table 2:** Substrate scope of (hetero)aryl halides^*a*^

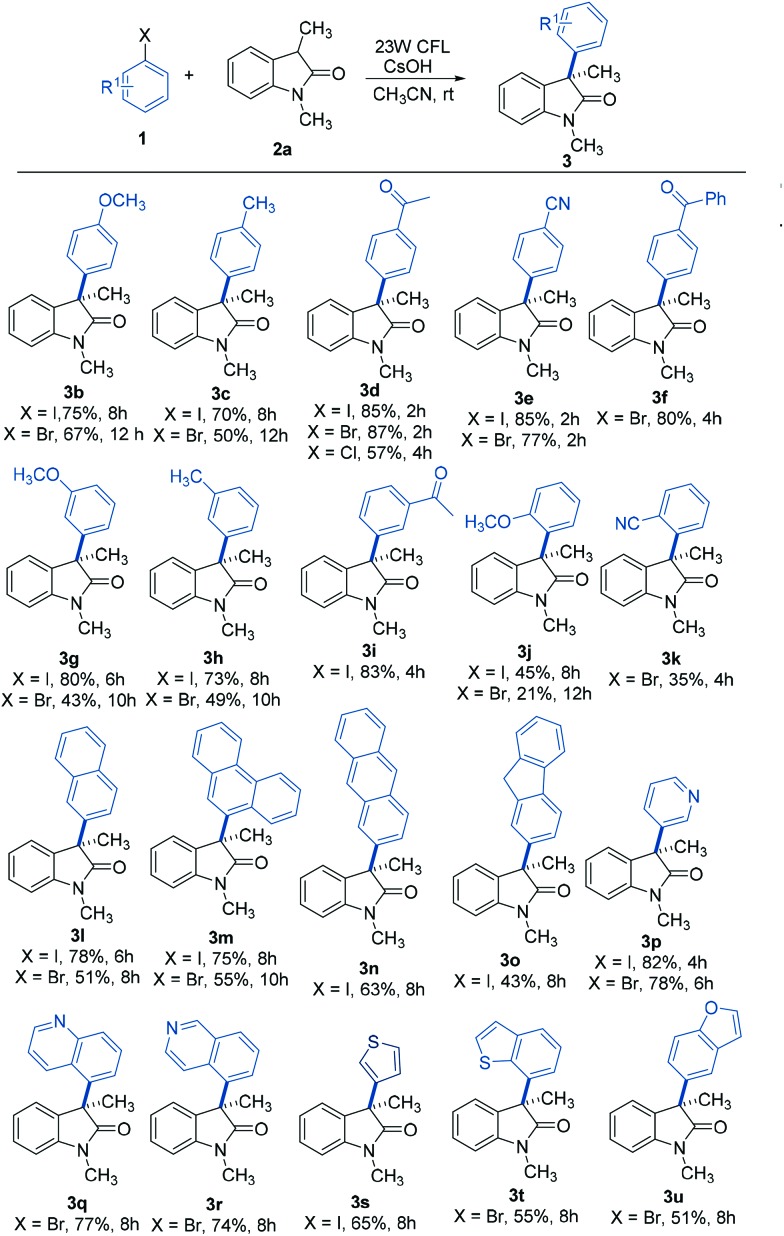

^*a*^General conditions: **1** (0.1 mmol), **2a** (0.2 mmol), CsOH (0.3 mmol), and CH_3_CN (1.0 mL). Isolated yields are provided.

To further explore the generality of this reaction, a variety of substituted oxindoles were examined (Table 3). Arylation was explored with an *N*-unprotected oxindole to provide the corresponding product **4a** in 68% yield. Pleasingly, arylation of other *N*-protected oxindoles (**4b** (*N*-allyl), **4c** (*N*-benzyl), and **4d** (*N*-MOM)) proceeded smoothly and gave satisfying results. Other alkyl groups at the C3 position were also compatible with this reaction system (**4e** and **4f**). It is important to note that tryptophol or tryptamine-containing substrates were well-tolerated, as were those with a 5-methyl or methoxy group, the motifs commonly found in bioactive oxindole-based compounds (**4g–4j**).^20^ Further studies revealed that oxindole substrates containing free amide, hydroxyl, or carboxyl groups (**4k–4m**) were also tolerated in our reaction system, avoiding the need for protecting groups.

**Table 3:** Substrate scope of substituted oxindoles^*a*^

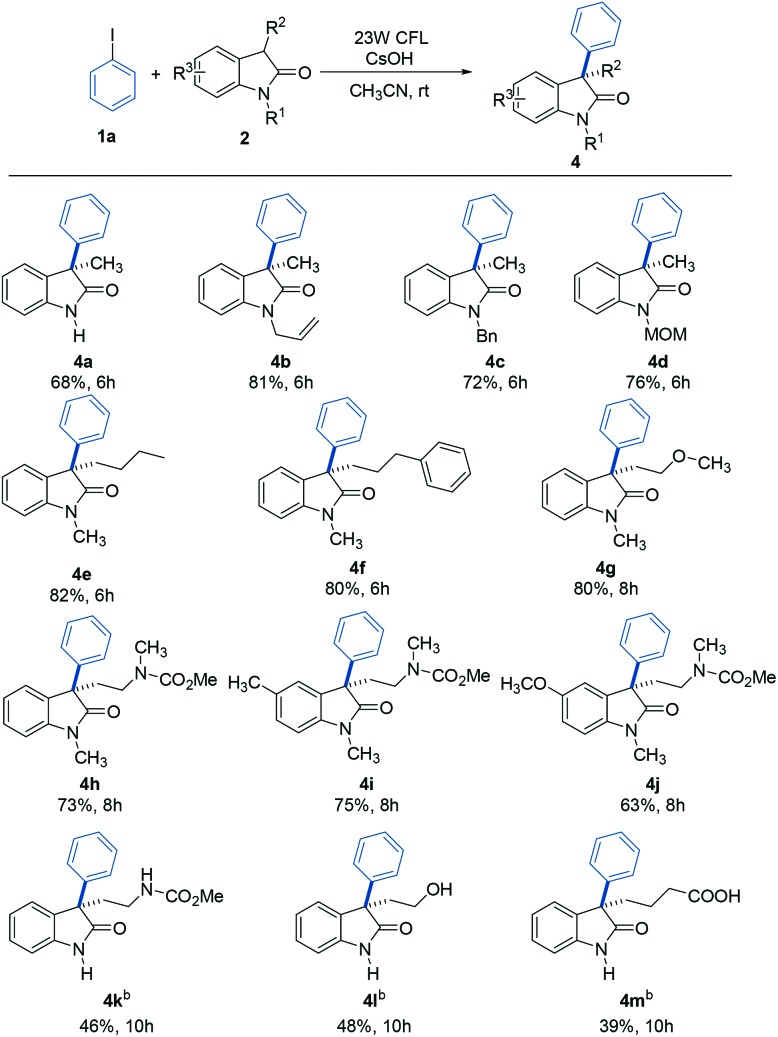

^*a*^General conditions: **1a** (0.1 mmol), **2** (0.2 mmol), CsOH (0.3 mmol), and CH_3_CN (1.0 mL). Isolated yields are provided.

^*b*^Using DMSO instead of acetonitrile as the solvent.

### Mechanistic investigation

**Fig. 3:**
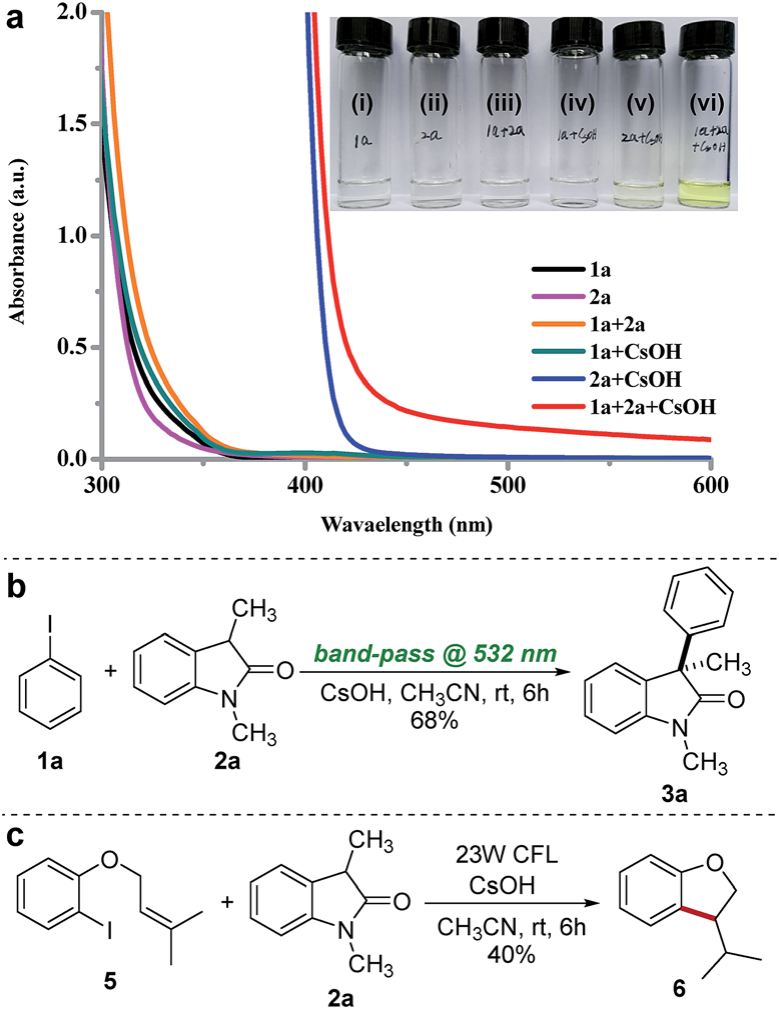
Mechanistic studies: (a) UV/Vis absorption spectra of acetonitrile solutions (0.1 M) of **1a** (i), **2a** (ii), mixture of **1a** and **2a** (iii), mixture of **1a** and CsOH (iv), mixture of **2a** and CsOH (v), and mixture of **1a**, **2a** and CsOH (iv). (b) Long wavelength experiment. (c) Intramolecular radical clock experiment.

To gain further insight into the mechanism of this photoinduced arylation reaction, we performed UV-vis spectroscopic measurements on various combinations of **1a**, **2a**, and CsOH in acetonitrile at a concentration of 0.1 M to investigate the formation of the proposed EDA complex between iodobenzene **1a** and the enolate of **2a**. Immediately after mixing an acetonitrile solution of the enolate of **2a**, which was *in situ* generated by the deprotonation of **2a** with CsOH, with iodobenzene **1a**, a dramatic color change was observed (Fig. 3a), and this was accompanied by a bathochromic shift in the UV-vis spectra, which is diagnostic of an EDA complex (red line, Fig. 3a).^15^,^16^,^21^ Interestingly, the absorption spectra of **2a** also show a red shift to the visible region upon CsOH addition (blue line, Fig. 3a). These results suggested that the enolate of **2a** may interact with visible light in two different ways, serving either as a donor in photon-absorbing EDA complex formation or as a photosensitizer upon direct excitation. To elucidate the reaction mechanism, we carried out a control experiment by using a 45 W white LED lamp, equipped with a band-pass filter at 532 nm (the enolate of **2a** is unable to absorb at this wavelength, blue line in Fig. 3a), which did not significantly alter the reaction efficiency (Fig. 3b). This result excluded the direct photoexcitation of the enolate of **2a** and suggested that the reaction proceeded likely by the EDA complex-driven photochemical mechanism.15e,^22^ A molar donor/acceptor ratio of 1 : 1 for the complex in solution was established by employing Job's method of continuous variation (full details of the stoichiometry determination are reported in ESI note 3†).^23^ Additional insight into the mechanism was obtained from an intramolecular radical clock experiment. When the reaction was carried out with substrate **5** under standard conditions, the exocyclic product **6** was generated through a 5-*exo-trig* radical cyclization (Fig. 3c), thereby confirming the radical nature of this reaction. Finally, the quantum yield for the arylation of **2a** was determined to be *Φ* = 11.1 (full details of the quantum yield determination are reported in ESI note 6†), indicating that a radical chain process might be involved in the reaction.15e,g,^22^,^24^

**Scheme 1:**
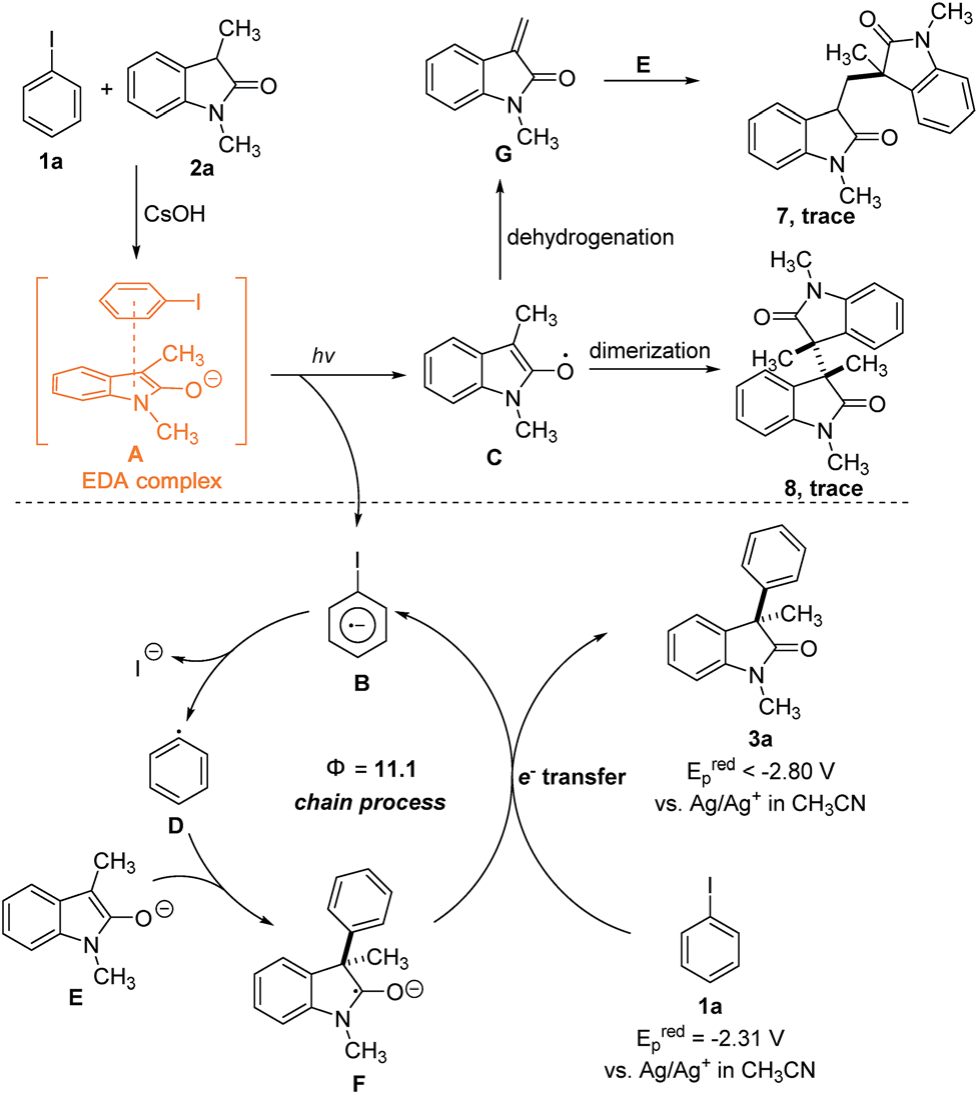
Proposed mechanism for visible-light-promoted arylation.

Based on the above observations, a plausible mechanism is outlined in Scheme 1. Initiation of the radical chain begins with the formation of EDA complex **A** between the enolate of **2a** and iodobenzene **1a**. The photochemical activity of **A** triggers a single-electron transfer (SET) to form a radical anion **B** and radical **C**. Fragmentation of the carbon–halogen within **B** releases the iodine anion and produces an aryl radical **D**. The electrophilic aryl radical is next trapped by the electron-rich enolate **E** to afford ketyl radical intermediate **F**, which is a strong single-electron reductant^25^ (the oxidation potential of **F** was estimated by measuring the reduction potential of **3a**, and no reduction waves were detected for **3a** in the range of –2.80 V–0 V *vs.* Ag/Ag^+^ in CH_3_CN, ESI Fig. 5†). This cyclic voltammetry result means that ketyl radical intermediate **F** can transfer an electron to iodobenzene **1a** (*E*_p_^red^ = –2.31 V *vs.* Ag/Ag^+^ in CH_3_CN, ESI Fig. 4†) to regenerate aryl radical **D** and propagate the radical chain. The SET process was strongly inhibited when the model reaction was performed with redox trap 1,4-dinitrobenzene (0.2 equiv., 42% after 6 h), which confirmed the electron transfer propagation pathway.^26^ Another aspect to consider is the fate of the oxindole radical **C**, which is an unproductive intermediate since it lies outside of the radical chain propagation cycle. We have obtained evidence that the radical **C** facilitated dimerization and dehydrogenation reactions in our system, since a trace amount of dimerization products **7** and **8** were detected in the gram scale experiment (ESI note 8†). These results further demonstrate the SET process within the EDA complex.

## Conclusions

In summary, we have disclosed a transition-metal-free protocol for the C–C cross-coupling of oxindoles and aryl halides under visible-light irradiation without the need for any external photoredox catalysts. The reactions were conducted at ambient temperature by using a household CFL bulb as the light source. Since no preactivation of the starting substrate is required and only the halogen anion is released as a byproduct, this transformation is highly atom- and step-economical. This new methodology offers a convenient and powerful synthetic tool for accessing 3-aryloxindole products under redox neutral conditions, and a wide range of 3-aryloxindole derivatives (33 examples) of pharmaceutical interest were constructed in a single step. The results of mechanistic experiments are consistent with a radical chain propagation pathway, wherein the aryl radical is generated by visible-light-induced electron transfer within the oxindole enolate-aryl halide EDA complex.

## Conflicts of interest

There are no conflicts to declare.

## Supplementary Material

Supplementary information
