# Supplementary material for: Transition-metal-free α-arylation of oxindoles via visible-light-promoted electron transfer
Source: Chem Sci. 2019 Jan 22;10(10):3049–53. doi: 10.1039/c8sc05170d (PMC6427940; doi:10.1039/c8sc05170d)
Supplement: Supplementary file 1 [file SC-010-C8SC05170D-s001.pdf]

Electronic Supplementary Material (ESI) for Chemical Science.
This journal is © The Royal Society of Chemistry 2019

Supplementary Information for

Transition-Metal-Free α-Arylation of Oxindoles *via* Visible-Light-Promoted Electron Transfer

Kangjiang Liang, Na Li, Yang Zhang, Tao Li, Chengfeng Xia*

Key Laboratory of Medicinal Chemistry for Natural Resources (Ministry of Education and Yunnan Province), School of Chemical Science and Technology, Yunnan University, 2 North Cuihu Road, Kunming 650091, China.

*Correspondence to: xiacf@ynu.edu.cn

Table of Contents

General Information 3

General Procedure for Visible-Light-Promoted α-Arylation of Oxindoles 3

Supplementary Note 1. ^{1}H NMR Spectroscopic Studies 4

Supplementary Note 2. UV-Vis Spectroscopic Measurement..5

Supplementary Note 3. Determination of the Stoichiometry of the EDA Complex..........................5

Supplementary Note 4. Long Wavelength Experiment. 6

Supplementary Note 5 Intramolecular Radical Clock Experiment..................................6

Supplementary Note 6. Quantum Yield Measurement 7

Supplementary Note 7. Cyclic Voltammetry Experiments..9

Supplementary Note 8. Gram Scale Experiment 10

Identification of Compounds 12

References..31

NMR Spectral Data 33

General Information

All reactions were performed under argon atmosphere using flame-dried glassware unless otherwise noted. CH_3CN was distilled over CaH_2 and rigorously degassed by freeze/pump/thaw. All reagents were commercially available and used without further purification unless indicated otherwise. Thin layer chromatographies were carried out on GF254 plates (0.25 mm layer thickness). Flash chromatography was performed with 200-300 mesh silica gels. Visualization of the developed chromatogram was performed by fluorescence quenching or by ceric ammonium molybdate, or $KMnO_4$ stain. Yields reported were for isolated, spectroscopically pure compounds.

^{1}H and ^{13}C NMR spectra were recorded on Bruker Avance 400 and 600 MHz spectrophotometers. Chemical shifts (δ) are expressed in ppm., and *J*-values are given in Hz. The residual solvent protons (^{1}H) or the solvent carbons (^{13}C) were used as internal standards. ESIMS and HRESIMS were taken on Agilent 6540 Q-TOF spectrometer. UV-Vis measurements were carried out on a HITACHI U-4100 spectrophotometer. Cyclic voltammetry studies was carried out on a CHI 760E electrochemical workstation (Shanghai CH Instruments Co., China).

General Procedure for Visible-Light-Promoted α-Arylation of Oxindoles

To an oven dried 10 mL glass tube with a magnetic stirring bar was added oxindoles (0.2 mmol) and $CsOH·H_2O$ (0.3 mmol). Then the reaction tube was allowed to be vacuumed and purged with Argon for three times. CH_3CN (1 mL) and (hetero)aryl halides (0.1 mmol) were carefully added under Argon. The reaction mixture was stirred under a household 23 W compact fluorescent light (CFL) bulb (the distance was about 7 cm) irradiation for the indicated time. Irradiation was stopped and the reaction was extracted with ethyl acetate (15 mL × 3). The combined organic layers were washed with brine, dried over anhydrous Na_2SO_4 and concentrated. The crude product was subjected to column chromatography (acetone/petroleum ether) on silica gel to afford the product.

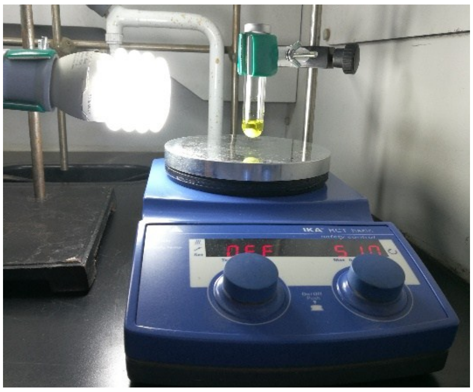

Supplementary Figure 1. Experimental setup for visible-light-promoted α-arylation of oxindoles

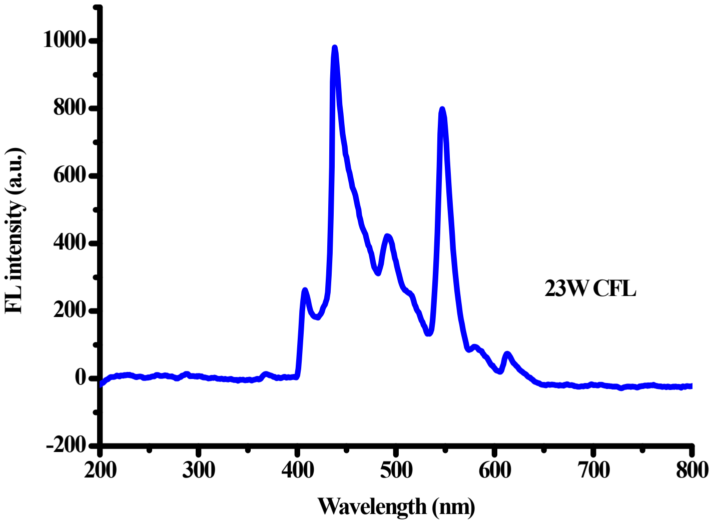

Supplementary Figure 2. Emission spectra of the 23W CFL lamp. The emission spectra was recorded on a Fluorescence Spectrophotometer F-4500.

Supplementary Note 1. ^{1}H NMR Spectroscopic Studies

The ^{1}H NMR analysis was made on a solution containing 8 mg of **2a** (0.05 mmol) and MOH (M = Cs^+, K^+ or Na^+) (0.075 mmol) in 0.5 mL of CD_3CN. Under these conditions (Argon and solvent were rigorously degassed by freeze/pump/thaw), all the MOHs could efficiently deprotonate the oxindole and complete enolate formation of **2a** was observed. Moreover, significant upfield peak shifting of aromatic hydrogens

were observed with the magnitude followed the order: Cs^+ solution > K^+ solution > Na^+ solution. These results provided evidences that varying alkali metal cations affecting reactivity might be due to secondary effect in the charge transfer process.[1]

Supplementary Note 2. UV-Vis Spectroscopic Measurement

The UV-Vis absorption spectra of acetonitrile solutions (0.1 M) of **1a**, **2a**, mixtures of **1a** and **2a**, mixtures of **1a** and CsOH, mixtures of **2a** and CsOH, and mixtures of **1a**, **2a** and CsOH were recorded on HITACHI U-4100 UV-Visible Spectrophotometer. The bathochromic shift to the visible spectral region is indicative of EDA complex formation.

Supplementary Note 3. Determination of the Stoichiometry of the EDA Complex

The stoichiometry of the EDA complex formed between iodobenzene **1a** and the enolate of **2a** (generated *in situ* by the deprotonation of **2a** with 1.1 equiv CsOH) in CH_3CN was determined using the Job's plot method. The Job's plot was constructed by measuring the absorption at 470 nm of CH_3CN solutions of different ratios of iodobenzene **1a** and the enolate of **2a**, where the total concentration of the two components remained constant at 0.1 M. The maximum absorbance is obtained at 50% molar fraction, indicating that the stoichiometry of the EDA complex is 1:1.

Supplementary Table 1. Absorbance of 0.1 M CH_3CN solutions of different molar ratios of iodobenzene **1a** and the enolate of **2a**.

entry	**1a**:the enolate of **2a** ratio	molar fraction of the enolate of **2a** (%)	absorbance at 470 nm
1	1.0:0.0	0	0
2	0.8:0.2	20	0.042
3	0.6:0.4	40	0.059
4	0.5:0.5	50	0.062
5	0.4:0.6	60	0.056
6	0.2:0.8	80	0.039
7	0.0:1.0	100	0

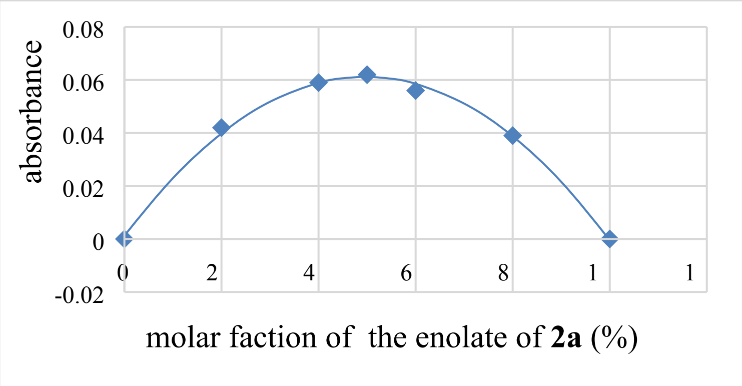

Supplementary Figure 3. Job's plot of iodobenzene **1a** and the enolate of **2a** in CH_3CN.

Supplementary Note 4. Long Wavelength Experiment.

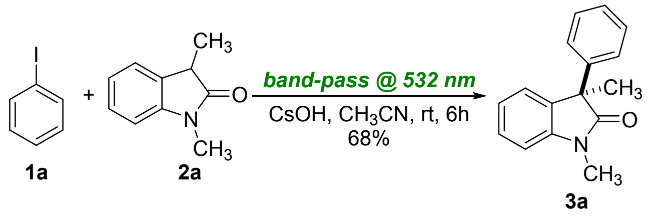

To an oven dried 10 mL glass tube with a magnetic stirring bar was added **2a** (32.2 mg, 0.2 mmol) and $CsOH{\cdot}H_2O$ (50.4 mg, 0.3 mmol). Then the reaction tube was allowed to be vacuumed and purged with Argon for three times. CH_3CN (1 mL) and iodobenzene **1a** (20.4 mg, 0.1 mmol) were carefully added under Argon. The reaction mixture was stirred under a 45 W white LED lamp which equipped with a band-pass filter at 532 nm irradiation for 6h. Irradiation was stopped and the reaction was extracted with ethyl acetate (15 mL × 3). The combined organic layers were washed with brine, dried over anhydrous Na_2SO_4 and concentrated. The crude product was subjected to column chromatography (acetone/petroleum ether = 1:5) on silica gel to afford **3a** as a colorless oil (16.1 mg, 68% yield).

Supplementary Note 5. Intramolecular Radical Clock Experiment

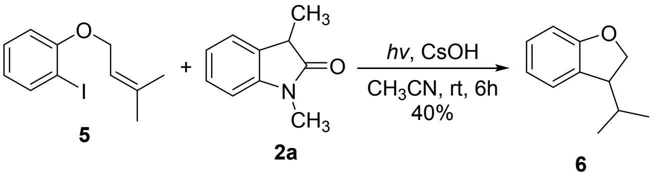

To an oven dried 10 mL glass tube with a magnetic stirring bar was added **2a**

(32.2 mg, 0.2 mmol) and $CsOH \cdot H_2O$ (50.4 mg, 0.3 mmol). Then the reaction tube was allowed to be vacuumed and purged with Argon for three times. CH_3CN (1 mL) and compound **5** (28.8 mg, 0.1 mmol) were carefully added under Argon. The reaction mixture was stirred under a household 23 W compact fluorescent light (CFL) bulb irradiation for 8h. Irradiation was stopped and the reaction was extracted with ethyl acetate (15 mL × 3). The combined organic layers were washed with brine, dried over anhydrous Na_2SO_4 and concentrated. The crude product was subjected to column chromatography (acetone/petroleum ether = 1:100) on silica gel to afford **6** (6.5 mg, 40% yield). ^1H NMR (600 MHz, $CDCl_3$): δ 7.18 (d, J = 7.4, 1H), 7.12 (td, J = 8.1, 0.7, 1H), 6.84 (td, J = 7.4, 0.9, 1H), 6.77 (d, J = 8.0, 1H), 4.51 (t, J = 9.1, 1H), 4.37 (dd, J = 9.0, 5.1, 1H), 3.35 – 3.29 (m, 1H), 2.02 – 1.92 (m, 1H), 0.95 (d, J = 6.8, 3H), 0.87 (d, J = 6.8, 3H); ^{13}C NMR (151 MHz, $CDCl_3$): δ 160.4, 129.4, 128.1, 125.1, 120.1, 109.3, 73.8, 48.2, 31.7, 19.8, 18.4; HR-ESI-MS (*m/z*): calcd. for $C_{11}H_{15}O$ $[M + H]^+$, 163.1117, found 163.1117.

Supplementary Note 6. Quantum Yield Measurement

Determination of the Photon Flux:

The photon flux of the LED setup was determined by standard ferrioxalate actinometry.[2] A 0.15 M ferrioxalate solution was prepared by dissolving 2.21 g of potassium ferrioxalate trihydrate in 30 mL of 0.05 M H_2SO_4. A buffered phenanthroline solution was prepared by dissolving 50 mg of 1,10-phenanthroline 11.25 g of sodium acetate in 50 mL of 0.5 M H_2SO_4. Both solutions were stored in the dark. To determine the photon flux of the LED setup, 2.0 mL of the ferrioxalate solution was placed in a quartz cuvette (l = 1.0 cm). The cuvette was placed 10 cm from an 18W blue LED lamp (λ_{max} at 455 nm) and irradiated for 30 s. After irradiation, 0.5 mL of the 1,10-phenanthroline solution was added to the cuvette. The mixture was then allowed to rest for 1 h to allow the ferrous ions to completely coordinate to the 1,10-phenanthroline. The absorbance of the solution was measured at 510 nm. The

absorbance of a non-irradiated sample was also measured.

Conversion was calculated using:

$$mol\,Fe^{2+} = \frac{V \cdot \Delta A}{l \cdot \varepsilon}$$

Where **V** is the total volume (0.0025 L) of the solution after addition of 1,10-phenanthroline, **ΔA** is the difference in absorbance at 510 nm between the irradiated and non-irradiated solutions, **l** is the path length (1.0 cm), and **ε** is the molar absorptivity at 510 nm (11,100 L mol^{-1} cm^{-1}).

The photon flux can be calculated using:

$$photon\,flux = \frac{mol\,Fe^{2+}}{\Phi \cdot t \cdot f}$$

Where ***Φ*** is the quantum yield for the ferrioxalate actinometer (a reported value of 0.85 for a 0.15 M solution at λ = 458 nm was used in the calculations),[3] **t** is the time (30 s), and **f** is the fraction of absorbed light at λ = 455 nm, where **f** = $1 - 10^{-A}$. The absorbance (A) of the ferrioxalate solution at λ = 455 nm was measured by UV/Vis spectroscopy to be 0.901, therefore **f** = 0.8744. The photon flux was calculated (average of three experiments) to be 8.54×10^{-9} einstein s^{-1}.

Sample calculation:

$$mol\,Fe^{2+} = \frac{0.0025\,L \cdot 0.849}{1.000\,cm \cdot 11100\,L\,mol^{-1}cm^{-1}} = 1.91 \times 10^{-7}\,mol$$

$$photon\,flux = \frac{1.91 \times 10^{-7}\,mol}{0.85 \cdot 30.0\,s \cdot 0.8744} = 8.57 \times 10^{-9}\,einstein\,s^{-1}$$

Determination of the Quantum Yield:

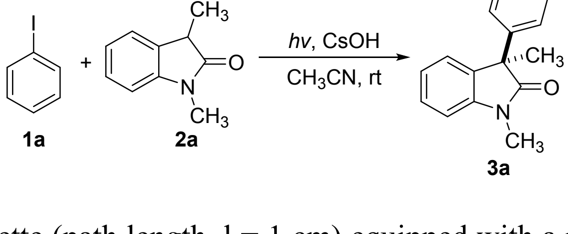

To a quartz cuvette (path length, l = 1 cm) equipped with a magnetic stir bar was

added **2a** (32.2 mg, 0.2 mmol) and $CsOH{\cdot}H_2O$ (50.4 mg, 0.3 mmol). The cuvette was purged with Argon before being sealed with a septum. CH_3CN (1 mL) and iodobenzene **1a** (20.4 mg, 0.1 mmol) were carefully added under Argon and the cuvette was further sealed with parafilm. The cuvette was positioned 10 cm away from an 18 W blue LED lamp (λ_{max} at 455 nm) and the reaction was irradiated for 1200 s (20 min). The yield was determined by ^1H NMR, using diphenylacetonitrile as a standard, to be (average of three experiments) 43% (4.3×10^{-5} mol).

The quantum yield (***Φ***) was then calculated using:

$$\Phi = \frac{mol\,product}{photon\,flux{\cdot}t{\cdot}f}$$

Where **t** is the time (1200 s) and **f** is the fraction of light absorbed by the reaction mixture at $\lambda = 455$ nm, where **f** $= 1 - 10^{-A}$ (the absorbance of the reaction mixture (A) was determined by UV/Vis spectroscopy to be 0.207, thus **f** = 0.3791). The photon flux of the LED setup was determined using standard ferrioxalate actinometry to be 8.54×10^{-9} einstein s^{-1}.

$$\Phi = \frac{4.3 \times 10^{-5}}{8.54 \times 10^{-9}{\cdot}1200{\cdot}0.3791} = 11.1$$

Supplementary Note 7. Cyclic Voltammetry Experiments

Tetrabutylammonium hexafluorophosphate (378 mg, 1.0 mmol) and **1a** (20.4 mg, 0.1 mmol) were dissolved in dry acetonitrile (10 mL) and the solution was vigorously bubbled with N_2 for 5 minutes prior to the measurement. The reduction potential was measured using a glassy carbon working electrode, a platinum wire counter electrode, and a KCl saturated Ag/AgCl reference electrode at 0.1 V/s scan rate. A completely irreversible reduction wave was observed with $E_p^{red} = -2.31$ V vs. Ag/Ag^+ in CH_3CN.

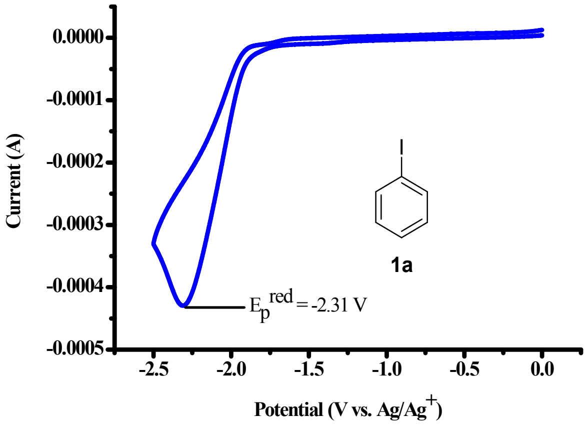

Supplementary Figure 4. The cyclic voltammogram of **1a** vs Ag/Ag^{+} in acetonitrile at 0.1V/s.

The cyclic voltammetry of **3a** was also carried out, using the same experimental conditions and concentrations. No reduction waves were detected for **3a** in the range -2.80 – 0 V.

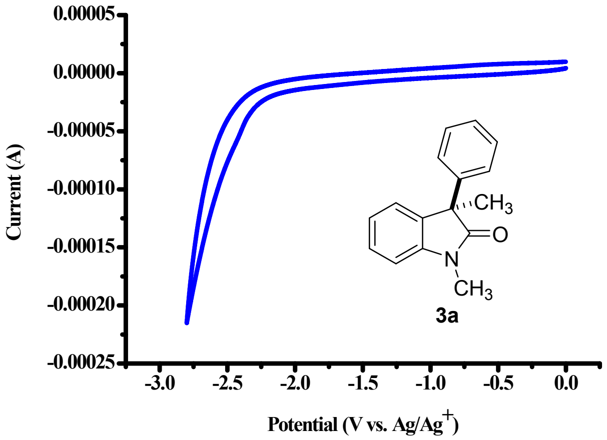

Supplementary Figure 5. The cyclic voltammogram of **3a** vs Ag/Ag^{+} in acetonitrile at 0.1V/s.

Supplementary Note 8. Gram Scale Experiment

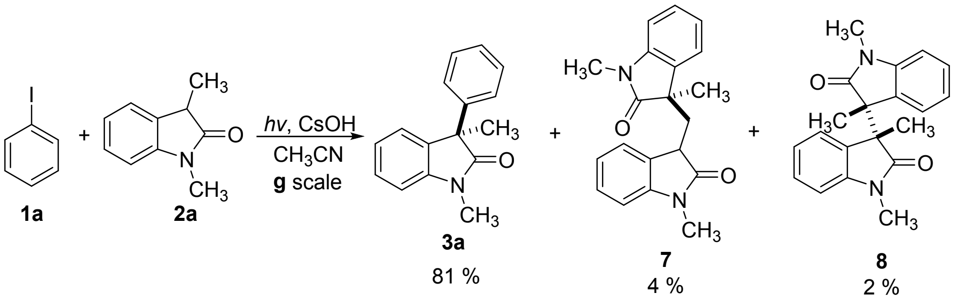

To an oven dried 100 mL round-bottom flask with a magnetic stirring bar was added **2a** (1.61 g, 10 mmol) and $CsOH\cdot H_2O$ (2.52 g, 15 mmol). Then the reaction tube was allowed to be vacuumed and purged with Argon for three times. CH_3CN (25 mL) and Iodobenzene **1a** (1.02 g, 5 mmol) were carefully added under Argon. The reaction mixture was stirred under four household 23 W compact fluorescent light (CFL) bulbs irradiation for 24 h. Irradiation was stopped and the reaction was extracted with ethyl acetate. The combined organic layers were washed with brine, dried over anhydrous Na_2SO_4 and concentrated. The crude product was subjected to column chromatography (acetone/petroleum ether = 1:5) on silica gel to afford **3a** as a colorless oil (0.96 g, 81% yield), **7** as a colorless oil (63.1 mg, 4% yield), and **8** as a colorless oil (33.5 mg, 2% yield).

(±)-1,3-dimethyl-3-((1-methyl-2-oxoindolin-3-yl)methyl)indolin-2-one (7): ^{1}H NMR (600 MHz, $CDCl_3$): δ 7.19 (d, *J* = 7.3, 1H), 7.16 (d, *J* = 7.3, 1H), 7.08-7.05 (m, 2H), 6.99-6.94 (m, 2H), 6.37-6.35 (m, 2H), 3.33 (d, *J* = 7.1, 1H), 2.96 (dd, *J* = 14.8, 7.3, 1H), 2.79 (d, *J* = 14.8, 1H), 2.67 (s, 6H), 1.30 (s, 3H); ^{13}C NMR (150 MHz, $CDCl_3$): δ 180.6, 177.3, 144.4, 143.3, 130.8, 127.7, 125.9, 125.5, 125.2, 121.5, 121.4, 107.2, 46.8, 43.8, 35.2, 26.4, 25.8; HR-ESI-MS (*m/z*): calcd. for $C_{20}H_{21}N_2O_2$ $[M + H]^+$, 321.1598, found 321.1599.

(±)-1,1',3,3'-tetramethyl-[3,3'-biindoline]-2,2'-dione (8): ^{1}H NMR (400 MHz, $CDCl_3$): δ 7.06 (d, *J* = 7.5, 2H), 7.04 – 6.99 (m, 2H), 6.82 (t, *J* = 7.6, 2H), 6.45 (d, *J* = 7.8, 2H), 3.09 (s, 6H), 1.75 (s, 6H); ^{13}C NMR (100 MHz, $CDCl_3$): δ 178.2, 142.6, 131.1, 128.1, 122.8, 121.8, 107.4, 51.1, 25.7, 16.1; calcd. for $C_{20}H_{20}N_2O_2Na$ $[M + Na]^+$, 343.1417, found 343.1418.

Identification of Compounds

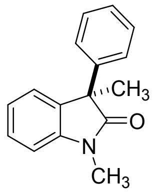

3a

(±)-1,3-dimethyl-3-phenylindolin-2-one (3a)

Prepared according to the general procedure using iodobenzene **1a** (20.4 mg, 0. 1 mmol), **2a** (32.2 mg, 0.2 mmol), $CsOH·H_2O$ (50.4 mg, 0.3 mmol), and CH_3CN (1 mL). Time of irradiation: 6 hours. The crude mixture was purified by silica gel chromatography (acetone/petroleum ether 1:5) to afford the product **3a** as a colorless oil (X = I, 21.1 mg, 85% yield; X = Br, 12.1 mg, 51% yield; X = Cl, 8.1 mg, 34% yield). ^{1}H NMR (400 MHz, $CDCl_3$): δ 7.37 – 7.21 (m, 6H), 7.19 (d, *J* = 7.3, 1H), 7.10 (t, *J* = 7.5, 1H), 6.92 (d, *J* = 7.8, 1H), 3.24 (s, 3H), 1.79 (s, 3H); ^{13}C NMR (100 MHz, $CDCl_3$): δ 179.5, 143.3, 140.8, 134.8, 128.6, 128.1, 127.3, 126.7, 124.2, 122.8, 108.3, 52.2, 26.5, 23.8; HR-ESI-MS (*m/z*): calcd. for $C_{16}H_{16}NO$ $[M + H]^+$, 238.1226, found 238.1230.

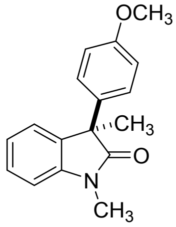

3b

(±)-3-(4-methoxyphenyl)-1,3-dimethylindolin-2-one (3b)

Prepared according to the general procedure using 4-iodoanisole (23.4 mg, 0. 1 mmol), **2a** (32.2 mg, 0.2 mmol), $CsOH·H_2O$ (50.4 mg, 0.3 mmol), and CH_3CN (1 mL). Time of irradiation: 8 hours. The crude mixture was purified by silica gel chromatography (acetone/petroleum ether 1:5) to afford the product **3b** as a colorless oil (X = I, 20.0 mg, 75% yield; X = Br, 17.9 mg, 67% yield). ^{1}H NMR (400 MHz, $CDCl_3$): δ 7.32 (t, *J* = 7.7, 1H), 7.22 (d, *J* = 8.7, 2H), 7.19 (d, *J* = 7.6, 1H), 7.10 (t, *J* = 7.5, 1H), 6.91 (d, *J* = 7.8, 1H), 6.82 (d, *J* = 8.6, 2H), 3.76 (s, 3H), 3.23 (s, 3H), 1.76 (s, 3H); ^{13}C NMR

(100 MHz, $CDCl_3$): δ 179.7, 158.7, 143.2, 135.0, 132.9, 128.1, 127.8, 124.2, 122.8, 113.9, 108.3, 55.3, 51.5, 26.5, 23.9; HR-ESI-MS (*m/z*): calcd. for $C_{17}H_{17}NO_2Na$ [M + Na]$^+$, 290.1151, found 290.1151.

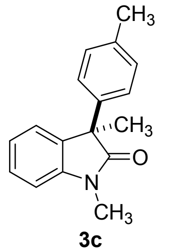

3c

(±)-1,3-dimethyl-3-(p-tolyl)indolin-2-one (3c)

Prepared according to the general procedure using 4-iodotoluene (21.8 mg, 0. 1 mmol), **2a** (32.2 mg, 0.2 mmol), $CsOH{\cdot}H_2O$ (50.4 mg, 0.3 mmol), and CH_3CN (1 mL). Time of irradiation: 8 hours. The crude mixture was purified by silica gel chromatography (acetone/petroleum ether 1:5) to afford the product **3c** as a colorless oil (X = I, 17.6 mg, 70% yield; X = Br, 12.6 mg, 50% yield). ^1H NMR (400 MHz, $CDCl_3$): δ 7.33 (t, J = 7.7, 1H), 7.21 – 7.17 (m, 3H), 7.16 – 7.05 (m, 3H), 6.92 (d, J = 7.8, 1H), 3.24 (s, 3H), 2.30 (s, 3H), 1.78 (s, 3H); ^{13}C NMR (100 MHz, $CDCl_3$): δ 179.6, 143.3, 137.8, 137.0, 135.0, 129.3, 128.1 126.5, 124.2, 122.8, 108.3, 51.9, 26.5, 23.7, 21.0; HR-ESI-MS (*m/z*): calcd. for $C_{17}H_{17}NONa$ [M + Na]$^+$, 274.1202, found 274.1206.

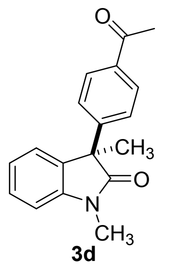

3d

(±)-3-(4-acetylphenyl)-1,3-dimethylindolin-2-one (3d)

Prepared according to the general procedure using 4-acetyliodobenzene (24.6 mg, 0. 1 mmol), **2a** (32.2 mg, 0.2 mmol), $CsOH{\cdot}H_2O$ (50.4 mg, 0.3 mmol), and CH_3CN (1 mL). Time of irradiation: 2 hours. The crude mixture was purified by silica gel

chromatography (acetone/petroleum ether 1:3) to afford the product **3d** as a colorless oil (X = I, 23.7 mg, 85% yield; X = Br, 24.3 mg, 87% yield; X = Cl, 15.9 mg, 57% yield). ^{1}H NMR (400 MHz, $CDCl_3$): δ 7.88 (d, *J* = 8.2, 2H), 7.40 (d, *J* = 8.2, 2H), 7.35 (t, *J* = 7.8, 1H), 7.17 (d, *J* = 7.3, 1H), 7.11 (t, *J* = 7.4, 1H), 6.95 (d, *J* = 7.8, 1H), 3.25 (s, 3H), 2.56 (s, 3H), 1.81 (s, 3H); ^{13}C NMR (100 MHz, $CDCl_3$): δ 197.7, 178.7, 146.1, 143.2, 136.0, 134.1, 128.6, 128.5, 127.0, 124.2, 123.0, 108.6, 52.3, 26.7, 26.6, 23.7; HR-ESI-MS (*m/z*): calcd. for $C_{18}H_{17}NO_2Na$ [M + Na]$^+$, 302.1151, found 302.1154.

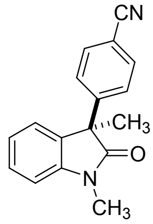

3e

(±)-4-(1,3-dimethyl-2-oxoindolin-3-yl)benzonitrile (3e)

Prepared according to the general procedure using 4-iodobenzonitrile (22.9 mg, 0. 1 mmol), **2a** (32.2 mg, 0.2 mmol), $CsOH\cdot H_2O$ (50.4 mg, 0.3 mmol), and CH_3CN (1 mL). Time of irradiation: 2 hours. The crude mixture was purified by silica gel chromatography (acetone/petroleum ether 1:4) to afford the product **3e** as a colorless oil (X = I, 22.3 mg, 85% yield; X = Br, 20.2 mg, 77% yield). ^{1}H NMR (400 MHz, $CDCl_3$): δ 7.58 (d, *J* = 8.4, 2H), 7.43 (d, *J* = 8.4, 2H), 7.37 (td, *J* = 7.6, 1.5, 1H), 7.22 – 7.10 (m, 2H), 6.95 (d, *J* = 7.8, 1H), 3.25 (s, 3H), 1.79 (s, 3H); ^{13}C NMR (100 MHz, $CDCl_3$): δ 178.2, 146.1, 143.2, 133.4, 132.3, 128.8, 127.7, 124.2, 123.2, 118.7, 111.2, 108.7, 52.3, 26.7, 23.8; HR-ESI-MS (*m/z*): calcd. for $C_{17}H_{14}N_2ONa$ [M + Na]$^+$, 285.0998, found 285.1006.

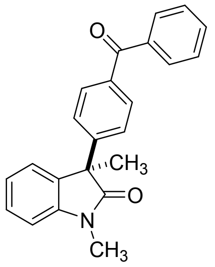

3f

(±)-3-(4-benzoylphenyl)-1,3-dimethylindolin-2-one (3f)

Prepared according to the general procedure using 4-bromobenzophenone (26.1 mg, 0. 1 mmol), **2a** (32.2 mg, 0.2 mmol), $CsOH·H_2O$ (50.4 mg, 0.3 mmol), and CH_3CN (1 mL). Time of irradiation: 4 hours. The crude mixture was purified by silica gel chromatography (acetone/petroleum ether 1:3) to afford the product **3f** as a colorless oil (X = Br, 27.3 mg, 80% yield). ^1H NMR (400 MHz, $CDCl_3$): δ 7.78 (d, J = 7.3, 2H), 7.73 (d, J = 8.4, 2H), 7.57 (t, J = 7.4, 1H), 7.49 – 7.42 (m, 4H), 7.40 – 7.32 (m, 1H), 7.20 (d, J = 7.2, 1H), 7.13 (t, J = 7.5, 1H), 6.95 (d, J = 7.8, 1H), 3.27 (s, 3H), 1.83 (s, 3H); ^{13}C NMR (100 MHz, $CDCl_3$): δ 196.2, 178.8, 145.4, 143.2, 137.5, 136.5, 134.2, 132.5, 130.3, 130.1, 128.5, 128.3, 126.7, 124.2, 123.0, 108.6, 52.4, 26.6, 23.8; HR-ESI-MS (*m/z*): calcd. for $C_{23}H_{19}NO_2Na$ $[M + Na]^+$, 364.1308, found 364.1329.

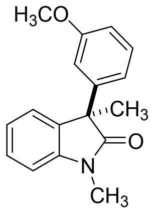

3g

(±)-3-(3-methoxyphenyl)-1,3-dimethylindolin-2-one (3g)

Prepared according to the general procedure using 3-iodoanisole (23.4 mg, 0. 1 mmol), **2a** (32.2 mg, 0.2 mmol), $CsOH·H_2O$ (50.4 mg, 0.3 mmol), and CH_3CN (1 mL). Time of irradiation: 6 hours. The crude mixture was purified by silica gel chromatography (acetone/petroleum ether 1:5) to afford the product **3g** as a colorless oil (X = I, 21.4 mg, 80% yield; X = Br, 11.5 mg, 43% yield). ^1H NMR (400 MHz, $CDCl_3$): δ 7.32 (td, J = 7.7, 1.0, 1H), 7.24 – 7.16 (m, 2H), 7.09 (t, J = 7.5, 1H), 6.92 – 6.87 (m, 3H), 6.78 (dd, J = 7.4, 1.7, 1H), 3.76 (s, 3H), 3.24 (s, 3H), 1.77 (s, 3H); ^{13}C NMR (100 MHz,

$CDCl_3$): δ 179.3, 159.6, 143.2, 142.4, 134.7, 129.5, 128.1, 124.2, 122.8, 119.1, 113.2, 112.0, 108.3, 55.2, 52.1, 26.5, 23.7; HR-ESI-MS (*m/z*): calcd. for $C_{17}H_{17}NO_2Na$ [M + Na]$^+$, 290.1151, found 290.1150.

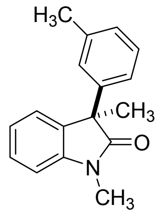

3h

(±)-1,3-dimethyl-3-(m-tolyl)indolin-2-one (3h)

Prepared according to the general procedure using 3-iodotoluene (21.8 mg, 0. 1 mmol), **2a** (32.2 mg, 0.2 mmol), $CsOH\cdot H_2O$ (50.4 mg, 0.3 mmol), and CH_3CN (1 mL). Time of irradiation: 8 hours. The crude mixture was purified by silica gel chromatography (acetone/petroleum ether 1:5) to afford the product **3h** as a colorless oil (X = I, 18.3 mg, 73% yield; X = Br, 12.3 mg, 49% yield). ^{1}H NMR (400 MHz, $CDCl_3$): δ 7.32 (td, *J* = 7.7, 1.2, 1H), 7.19 – 7.17 (m, 2H), 7.12 – 7.06 (m, 4H), 6.92 (d, *J* = 7.8, 1H), 3.25 (s, 3H), 2.30 (s, 3H), 1.77 (s, 3H); ^{13}C NMR (100 MHz, $CDCl_3$): δ 179.6, 143.2, 140.7, 138.2, 135.1, 128.4, 128.1, 127.3, 124.2, 123.7, 122.8, 108.3, 52.1, 26.5, 23.7, 21.6; HR-ESI-MS (*m/z*): calcd. for $C_{17}H_{17}NONa$ [M + Na]$^+$, 274.1202, found 274.1203.

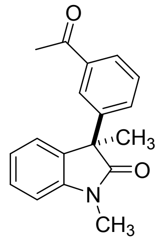

3i

(±)-3-(3-acetylphenyl)-1,3-dimethylindolin-2-one (3i)

Prepared according to the general procedure using 3-iodoacetophenone (24.6 mg, 0. 1 mmol), **2a** (32.2 mg, 0.2 mmol), $CsOH\cdot H_2O$ (50.4 mg, 0.3 mmol), and CH_3CN (1 mL). Time of irradiation: 4 hours. The crude mixture was purified by silica gel

chromatography (acetone/petroleum ether 1:3) to afford the product **3i** as a colorless oil (X = I, 22.6 mg, 83% yield). ^{1}H NMR (400 MHz, $CDCl_3$): δ 7.94 (d, *J* = 1.6, 1H), 7.83 (d, *J* = 7.7, 1H), 7.51 – 7.46 (m, 1H), 7.41 – 7.31 (m, 2H), 7.21 – 7.15 (m, 1H), 7.11 (t, *J* = 7.5, 1H), 6.94 (d, *J* = 7.8, 1H), 3.26 (s, 3H), 2.56 (s, 3H), 1.82 (s, 3H); ^{13}C NMR (100 MHz, $CDCl_3$): δ 198.0, 179.0, 143.2, 141.5, 137.4, 134.2, 131.6, 128.8, 128.5, 127.4, 126.4, 124.1, 123.1, 108.6, 52.1, 26.7, 26.6, 24.0; HR-ESI-MS (*m/z*): calcd. for $C_{18}H_{17}NO_2Na$ $[M + Na]^+$, 302.1151, found 302.1151.

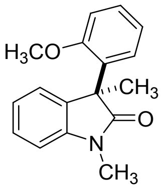

3j

(±)-3-(2-methoxyphenyl)-1,3-dimethylindolin-2-one (3j)

Prepared according to the general procedure using 2-iodoanisole (23.4 mg, 0. 1 mmol), **2a** (32.2 mg, 0.2 mmol), $CsOH\cdot H_2O$ (50.4 mg, 0.3 mmol), and CH_3CN (1 mL). Time of irradiation: 8 hours. The crude mixture was purified by silica gel chromatography (acetone/petroleum ether 1:5) to afford the product **3j** as a colorless oil (X = I, 12.0 mg, 45% yield; X = Br, 5.6 mg, 21% yield). ^{1}H NMR (400 MHz, $CDCl_3$): δ 7.58 (dd, *J* = 7.7, 1.1, 1H), 7.30 – 7.19 (m, 2H), 7.04 (t, *J* = 7.3, 1H), 6.93 (t, *J* = 7.4, 1H), 6.89 – 6.84 (m, 2H), 6.75 (d, *J* = 8.1, 1H), 3.41 (s, 3H), 3.32 (s, 3H), 1.70 (s, 3H); ^{13}C NMR (100 MHz, $CDCl_3$): δ 181.1, 157.0, 143.6, 135.7, 129.8, 128.8, 127.6, 127.3, 122.2, 120.9, 112.0, 107.4, 55.9, 49.8, 26.4, 23.4; HR-ESI-MS (*m/z*): calcd. for $C_{17}H_{17}NO_2Na$ $[M + Na]^+$, 290.1151, found 290.1153.

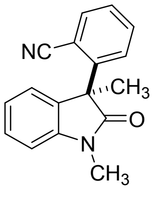

3k

(±)-2-(1,3-dimethyl-2-oxoindolin-3-yl)benzonitrile (3k)

Prepared according to the general procedure using 2-bromobenzonitrile (18.2 mg, 0. 1 mmol), **2a** (32.2 mg, 0.2 mmol), $CsOH \cdot H_2O$ (50.4 mg, 0.3 mmol), and CH_3CN (1 mL). Time of irradiation: 4 hours. The crude mixture was purified by silica gel chromatography (acetone/petroleum ether 1:5) to afford the product **3k** as a colorless oil (X = Br, 9.2 mg, 35% yield). ^1H NMR (400 MHz, $CDCl_3$): δ 7.78 (d, J = 8.0, 1H), 7.72 – 7.62 (m, 1H), 7.57 (d, J = 7.6, 1H), 7.39 – 7.33 (m, 2H), 7.03 (t, J = 7.5, 1H), 6.96 (d, J = 7.8, 1H), 6.89 (d, J = 7.3, 1H), 3.34 (s, 3H), 1.85 (s, 3H); ^{13}C NMR (100 MHz, $CDCl_3$): δ 178.6, 144.2, 143.4, 135.1, 134.0, 132.8, 128.9, 128.1, 127.9, 123.0, 116.8, 111.9, 108.8, 52.3, 26.5, 24.3; HR-ESI-MS (*m/z*): calcd. for $C_{17}H_{14}N_2ONa$ $[M + Na]^+$, 285.0998, found 285.0992.

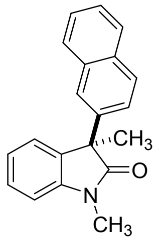

3l

(±)-1,3-dimethyl-3-(naphthalen-2-yl)indolin-2-one (3l)

Prepared according to the general procedure using 2-iodonaphthalene (25.4 mg, 0. 1 mmol), **2a** (32.2 mg, 0.2 mmol), $CsOH \cdot H_2O$ (50.4 mg, 0.3 mmol), and CH_3CN (1 mL). Time of irradiation: 6 hours. The crude mixture was purified by silica gel chromatography (acetone/petroleum ether 1:5) to afford the product **3l** as a colorless oil (X = I, 22.4 mg, 78% yield; X = Br, 14.6 mg, 51% yield). ^1H NMR (400 MHz, $CDCl_3$): δ 7.78 – 7.74 (m, 4H), 7.46 – 7.44 (m, 2H), 7.37 – 7.35 (m, 2H), 7.22 (d, J = 7.3, 1H), 7.12 (t, J = 7.5, 1H), 6.96 (d, J = 7.8, 1H), 3.28 (s, 3H), 1.90 (s, 3H); ^{13}C NMR (100 MHz, $CDCl_3$): δ 179.5, 143.3, 138.1, 134.9, 133.2, 132.5, 128.4, 128.2, 128.1, 127.5, 126.1, 126.0, 125.3, 124.9, 124.3, 122.9, 108.4, 52.3, 26.6, 23.6; HR-ESI-MS (*m/z*): calcd. for $C_{20}H_{18}NO$ $[M + H]^+$, 288.1383, found 288.1389.

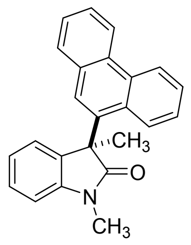

3m

(±)-1,3-dimethyl-3-(phenanthren-9-yl)indolin-2-one (3m)

Prepared according to the general procedure using 9-iodophenanthrene (30.4 mg, 0. 1 mmol), **2a** (32.2 mg, 0.2 mmol), $CsOH·H_2O$ (50.4 mg, 0.3 mmol), and CH_3CN (1 mL). Time of irradiation: 8 hours. The crude mixture was purified by silica gel chromatography (acetone/petroleum ether 1:5) to afford the product **3m** as a colorless oil (X = I, 25.3 mg, 75% yield; X = Br, 18.5 mg, 55% yield). ^1H NMR (400 MHz, $CDCl_3$): δ 8.67 (d, *J* = 8.3, 1H), 8.63 (d, *J* = 7.8, 1H), 8.12 (s, 1H), 7.97 (d, *J* = 7.5, 1H), 7.70 – 7.58 (m, 2H), 7.49 (t, *J* = 7.6, 1H), 7.32 (t, *J* = 7.7, 1H), 7.23 (d, *J* = 7.8, 1H), 7.07 (d, *J* = 7.8, 1H), 6.95 – 6.86 (m, 3H), 3.46 (s, 3H), 1.98 (s, 3H); ^{13}C NMR (100 MHz, $CDCl_3$): δ 180.4, 142.3, 136.5, 133.4, 131.3, 130.6, 130.3, 129.0, 128.1, 128.0, 127.1, 126.8, 126.7, 126.1, 124.3, 123.3, 123.2, 122.8, 122.4, 108.8, 52.6, 27.2, 26.8; HR-ESI-MS (*m/z*): calcd. for $C_{24}H_{19}NONa$ $[M + Na]^+$, 360.1359, found 360.1368.

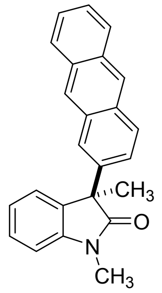

3n

(±)-3-(anthracen-2-yl)-1,3-dimethylindolin-2-one (3n)

Prepared according to the general procedure using 2-iodoanthracene (30.4 mg, 0. 1 mmol), **2a** (32.2 mg, 0.2 mmol), $CsOH·H_2O$ (50.4 mg, 0.3 mmol), and CH_3CN (1 mL). Time of irradiation: 8 hours. The crude mixture was purified by silica gel chromatography (acetone/petroleum ether 1:5) to afford the product **3n** as a colorless

oil (X = I, 21.2 mg, 63% yield). ^{1}H NMR (600 MHz, $CDCl_3$): δ 8.36 (s, 1H), 8.34 (s, 1H), 7.98 – 7.94 (m, 2H), 7.92 (s, 1H), 7.89 (d, *J* = 9.0, 1H), 7.44 (s, 1H), 7.42 (s, 1H), 7.35 (td, *J* = 7.8, 1.1, 1H), 7.31 (dd, *J* = 8.9, 1.6, 1H), 7.23 (d, *J* = 6.8, 1H), 7.12 (t, *J* = 7.5, 1H), 6.95 (d, *J* = 7.8, 1H), 3.28 (s, 3H), 1.92 (s, 3H); ^{13}C NMR (150 MHz, $CDCl_3$): δ 179.4, 143.4, 137.5, 134.7, 131.9, 131.8, 131.4, 130.8, 128.8, 128.3, 128.2, 126.5, 125.8, 125.4, 125.3, 124.7, 124.3, 122.9, 108.4, 52.5, 26.6, 23.4; HR-ESI-MS (*m/z*): calcd. for $C_{24}H_{19}NONa$ $[M + Na]^+$, 360.1359, found 360.1360.

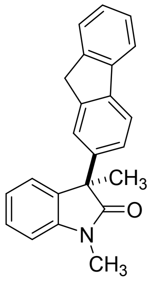

3o

(±)-3-(9H-fluoren-2-yl)-1,3-dimethylindolin-2-one (3o)

Prepared according to the general procedure using 2-iodofluorene (29.2 mg, 0. 1 mmol), **2a** (32.2 mg, 0.2 mmol), $CsOH{\cdot}H_2O$ (50.4 mg, 0.3 mmol), and CH_3CN (1 mL). Time of irradiation: 8 hours. The crude mixture was purified by silica gel chromatography (acetone/petroleum ether 1:5) to afford the product **3o** as a colorless oil (X = I, 14.0 mg, 43% yield). ^{1}H NMR (400 MHz, $CDCl_3$): δ 7.73 (d, *J* = 7.5, 1H), 7.68 (d, *J* = 8.0, 1H), 7.52 – 7.50 (m, 2H), 7.39 – 7.27 (m, 4H), 7.23 (d, *J* = 7.4, 1H), 7.12 (t, *J* = 7.5, 1H), 6.95 (d, *J* =7 .8, 1H), 3.84 (s, 2H), 3.27 (s, 3H), 1.84 (s, 3H); ^{13}C NMR (100 MHz, $CDCl_3$): δ 179.7, 143.6, 143.4, 143.3, 141.3, 140.9, 139.3, 135.2, 128.1, 126.7, 125.3, 125.0, 124.3, 123.4, 122.9, 119.9, 119.8, 108.4, 52.3, 37.0, 26.6, 24.0; HR-ESI-MS (*m/z*): calcd. for $C_{23}H_{20}NO$ $[M + H]^+$, 326.1539, found 326.1541.

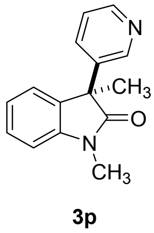

3p

(±)-1,3-dimethyl-3-(pyridin-3-yl)indolin-2-one (3p)

Prepared according to the general procedure using 3-iodopyridine (20.5 mg, 0. 1 mmol), **2a** (32.2 mg, 0.2 mmol), $CsOH{\cdot}H_2O$ (50.4 mg, 0.3 mmol), and CH_3CN (1 mL). Time of irradiation: 4 hours. The crude mixture was purified by silica gel chromatography (acetone/petroleum ether 1:3) to afford the product **3p** as a colorless oil (X = I, 19.5 mg, 82% yield; X = Br, 18.6 mg, 78% yield). ^{1}H NMR (400 MHz, $CDCl_3$): δ 8.51 – 8.49 (m, 2H), 7.75 – 7.69 (m, 1H), 7.36 (td, *J* = 7.7, 1.2, 1H), 7.29 – 7.19 (m, 2H), 7.13 (t, *J* = 7.5, 1H), 6.95 (d, *J* = 7.8, 1H), 3.25 (s, 3H), 1.81 (s, 3H); ^{13}C NMR (100 MHz, $CDCl_3$): δ 178.6, 148.5, 148.2, 143.2, 136.5, 134.6, 133.3, 128.6, 124.2, 123.4, 123.1, 108.7, 50.6, 26.6, 23.8; HR-ESI-MS (*m/z*): calcd. for $C_{15}H_{15}N_2O$ $[M + H]^+$, 239.1179, found 239.1181.

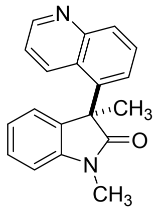

3q

(±)-1,3-dimethyl-3-(quinolin-5-yl)indolin-2-one (3q)

Prepared according to the general procedure using 5-bromoquinoline (20.8 mg, 0. 1 mmol), **2a** (32.2 mg, 0.2 mmol), $CsOH{\cdot}H_2O$ (50.4 mg, 0.3 mmol), and CH_3CN (1 mL). Time of irradiation: 8 hours. The crude mixture was purified by silica gel chromatography (acetone/petroleum ether 1:3) to afford the product **3q** as a colorless oil (X = Br, 22.2 mg, 77% yield). ^{1}H NMR (400 MHz, $CDCl_3$): δ 8.76 (dd, *J* = 4.1, 1.2, 1H), 8.10 (d, *J* = 8.4, 1H), 7.90 (d, *J* = 7.2, 1H), 7.83 – 7.73 (m, 1H), 7.33 (t, *J* = 7.7, 1H), 7.25 (d, *J* = 8.4, 1H), 7.12 – 7.03 (m, 2H), 6.96 (t, *J* = 7.5, 1H), 6.84 (d, *J* = 7.4, 1H), 3.44 (s, 3H), 1.91 (s, 3H); ^{13}C NMR (100 MHz, $CDCl_3$): δ 180.0, 149.5, 149.1, 142.0, 136.4, 135.7, 132.0, 130.5, 128.7, 128.3, 126.8, 126.7, 123.4, 123.1, 121.1, 108.9, 52.3, 26.8, 26.7; HR-ESI-MS (*m/z*): calcd. for $C_{19}H_{17}N_2O$ $[M + H]^+$, 289.1335, found 289.1344.

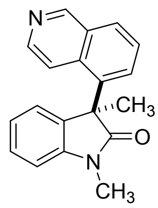

3r

(±)-3-(isoquinolin-5-yl)-1,3-dimethylindolin-2-one (3r)

Prepared according to the general procedure using 5-bromoisoquinoline (20.8 mg, 0. 1 mmol), **2a** (32.2 mg, 0.2 mmol), $CsOH\cdot H_2O$ (50.4 mg, 0.3 mmol), and CH_3CN (1 mL). Time of irradiation: 8 hours. The crude mixture was purified by silica gel chromatography (acetone/petroleum ether 1:3) to afford the product **3r** as a colorless oil (X = Br, 21.3 mg, 74% yield). ^1H NMR (400 MHz, $CDCl_3$): δ 9.18 (s, 1H), 8.20 (d, *J* = 6.2, 1H), 8.05 (d, *J* = 7.3, 1H), 7.95 (d, *J* = 8.2, 1H), 7.69 (t, *J* = 7.8, 1H), 7.35 (t, *J* = 7.7, 1H), 7.08 (d, *J* = 7.8, 1H), 6.97 (t, *J* = 7.5, 1H), 6.82 (d, *J* = 7.3, 1H), 6.68 (d, *J* = 6.2, 1H), 3.45 (s, 3H), 1.91 (s, 3H); ^{13}C NMR (100 MHz, $CDCl_3$): δ 179.8, 153.5, 143.5, 142.2, 135.9, 134.7, 134.2, 130.3, 129.5, 128.6, 128.4, 126.7, 123.4, 123.0, 116.6, 108.9, 52.2, 26.8, 26.4; HR-ESI-MS (*m/z*): calcd. for $C_{19}H_{17}N_2O$ $[M + H]^+$, 289.1335, found 289.1343.

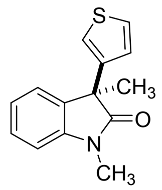

3s

(±)-1,3-dimethyl-3-(thiophen-3-yl)indolin-2-one (3s)

Prepared according to the general procedure using 3-iodothiophene (21.0 mg, 0. 1 mmol), **2a** (32.2 mg, 0.2 mmol), $CsOH\cdot H_2O$ (50.4 mg, 0.3 mmol), and CH_3CN (1 mL). Time of irradiation: 8 hours. The crude mixture was purified by silica gel chromatography (acetone/petroleum ether 1:5) to afford the product **3s** as a colorless oil (X = I, 15.8 mg, 65% yield). ^1H NMR (400 MHz, $CDCl_3$): δ 7.33 (t, *J* = 7.7, 1H), 7.30 – 7.24 (m, 2H), 7.15 – 7.03 (m, 3H), 6.90 (d, *J* = 7.8, 1H), 3.23 (s, 3H), 1.76 (s, 3H); ^{13}C NMR (100 MHz, $CDCl_3$): δ 178.7, 143.1, 141.6, 134.2, 128.3, 126.5, 126.1,

123.9, 122.8, 121.5, 108.3, 50.0, 26.5, 24.5; HR-ESI-MS (*m/z*): calcd. for $C_{14}H_{13}NOSNa$ [M + Na]$^+$, 266.0610, found 266.0612.

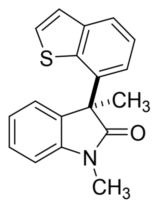

3t

(±)-3-(benzo[b]thiophen-7-yl)-1,3-dimethylindolin-2-one (3t)

Prepared according to the general procedure using 7-bromobenzothiophene (21.3 mg, 0. 1 mmol), **2a** (32.2 mg, 0.2 mmol), $CsOH·H_2O$ (50.4 mg, 0.3 mmol), and CH_3CN (1 mL). Time of irradiation: 8 hours. The crude mixture was purified by silica gel chromatography (acetone/petroleum ether 1:5) to afford the product **3t** as a colorless oil (X = Br, 16.1 mg, 55% yield). ^1H NMR (400 MHz, $CDCl_3$): δ 7.76 (d, *J* = 7.9, 1H), 7.64 (d, *J* = 7.5, 1H), 7.45 (t, *J* = 7.7, 1H), 7.36 (t, *J* = 7.5, 1H), 7.24 (d, *J* = 5.5, 1H), 7.17 (d, *J* = 5.5, 1H), 7.01 – 6.95 (m, 3H), 3.36 (s, 3H), 1.94 (s, 3H); ^{13}C NMR (100 MHz, $CDCl_3$): δ 178.5, 144.1, 140.7, 137.7, 134.2, 133.6, 128.6, 126.2, 124.5, 124.0, 123.9, 123.5, 123.2, 123.0, 108.4, 52.6, 26.6, 23.8; HR-ESI-MS (*m/z*): calcd. for $C_{18}H_{15}NOSNa$ [M + Na]$^+$, 316.0767, found 316.0772.

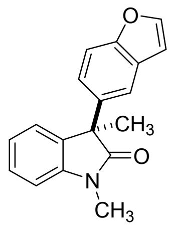

3u

(±)-3-(benzofuran-5-yl)-1,3-dimethylindolin-2-one (3u)

Prepared according to the general procedure using 5-bromobenzofuran (19.7 mg, 0. 1 mmol), **2a** (32.2 mg, 0.2 mmol), $CsOH·H_2O$ (50.4 mg, 0.3 mmol), and CH_3CN (1 mL). Time of irradiation: 8 hours. The crude mixture was purified by silica gel chromatography (acetone/petroleum ether 1:5) to afford the product **3u** as a colorless

oil (X = Br, 14.1 mg, 51% yield). ^{1}H NMR (400 MHz, $CDCl_3$): δ 7.57 (dd, *J* = 11.9, 1.9, 2H), 7.40 (d, *J* = 8.7, 1H), 7.34 (td, *J* = 7.7, 1.1, 1H), 7.24 – 7.17 (m, 2H), 7.11 (t, *J* = 7.5, 1H), 6.94 (d, *J* = 7.8, 1H), 6.70 (d, *J* = 1.3, 1H), 3.26 (s, 3H), 1.84 (s, 3H); ^{13}C NMR (100 MHz, $CDCl_3$): δ 179.8, 145.4, 143.2, 135.3, 128.1, 127.5, 124.2, 123.2, 122.8, 119.3, 111.3, 108.4, 106.8, 52.1, 26.5, 24.2; HR-ESI-MS (*m/z*): calcd. for $C_{18}H_{15}NO_2Na$ $[M + Na]^+$, 300.0995, found 300.1003.

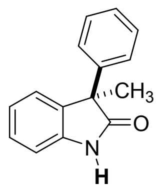

4a

(±)-3-methyl-3-phenylindolin-2-one (4a)

Prepared according to the general procedure using iodobenzene **1a** (20.4 mg, 0. 1 mmol), 3-methylindolin-2-one (29.4 mg, 0.2 mmol), $CsOH{\cdot}H_2O$ (50.4 mg, 0.3 mmol), and CH_3CN (1 mL). Time of irradiation: 6 hours. The crude mixture was purified by silica gel chromatography (acetone/petroleum ether 1:4) to afford the product **4a** as a colorless oil (15.2 mg, 68% yield). ^{1}H NMR (400 MHz, $CDCl_3$): δ 8.23 (s, 1H), 7.36 – 7.21 (m, 6H), 7.14 (d, *J* = 7.4, 1H), 7.06 (t, *J* = 7.5, 1H), 6.96 (d, *J* = 7.8, 1H), 1.82 (s, 3H); ^{13}C NMR (100 MHz, $CDCl_3$): δ 181.6, 140.5, 140.2, 135.5, 128.6, 128.1, 127.3, 126.6, 124.5, 122.8, 110.0, 52.6, 23.5; HR-ESI-MS (*m/z*): calcd. for $C_{15}H_{13}NONa$ $[M + Na]^+$, 246.0889, found 246.0888.

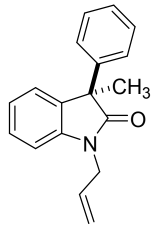

4b

(±)-1-allyl-3-methyl-3-phenylindolin-2-one (4b)

Prepared according to the general procedure using iodobenzene **1a** (20.4 mg, 0. 1

mmol), 1-allyl-3-methylindolin-2-one (37.4 mg, 0.2 mmol), $CsOH{\cdot}H_2O$ (50.4 mg, 0.3 mmol), and CH_3CN (1 mL). Time of irradiation: 6 hours. The crude mixture was purified by silica gel chromatography (acetone/petroleum ether 1:5) to afford the product **4b** as a colorless oil (21.3 mg, 81% yield). ^1H NMR (400 MHz, $CDCl_3$): δ 7.34 – 7.22 (m, 6H), 7.19 (d, *J* = 7.3, 1H), 7.08 (t, *J* = 7.5, 1H), 6.91 (d, *J* = 7.8, 1H), 5.90 – 5.78 (m, 1H), 5.27 – 5.15 (m, 2H), 4.39 – 4.33 (m, 2H), 1.81 (s, 3H); ^{13}C NMR (100 MHz, $CDCl_3$): δ 179.2, 142.4, 134.9, 131.5, 128.6, 128.0, 127.3, 126.6, 124.3, 122.8, 117.5, 109.2, 52.1, 42.4, 23.9; HR-ESI-MS (*m/z*): calcd. for $C_{18}H_{17}NONa$ $[M + Na]^+$, 286.1202, found 286.1197.

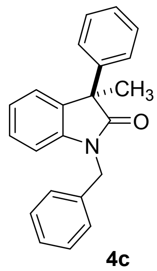

4c

(±)-1-benzyl-3-methyl-3-phenylindolin-2-one (4c)

Prepared according to the general procedure using iodobenzene **1a** (20.4 mg, 0. 1 mmol), 1-benzyl-3-methylindolin-2-one (47.4 mg, 0.2 mmol), $CsOH{\cdot}H_2O$ (50.4 mg, 0.3 mmol), and CH_3CN (1 mL). Time of irradiation: 6 hours. The crude mixture was purified by silica gel chromatography (acetone/petroleum ether 1:5) to afford the product **4c** as a colorless oil (22.5 mg, 72% yield). ^1H NMR (400 MHz, $CDCl_3$): δ 7.32 – 7.21 (m, 11H), 7.18 (d, *J* = 8.6, 2H), 7.05 (t, *J* = 7.5, 1H), 6.79 (d, *J* = 7.7, 1H), 4.94 (q, *J* = 15.7, 2H), 1.85 (s, 3H); ^{13}C NMR (100 MHz, $CDCl_3$): δ 179.6, 142.3, 140.8, 136.0, 135.0, 128.8, 128.6, 128.0, 127.6, 127.3, 127.2, 126.7, 124.2, 122.8, 109.4, 52.2, 43.9, 23.8; HR-ESI-MS (*m/z*): calcd. for $C_{22}H_{19}NONa$ $[M + Na]^+$, 336.1359, found 336.1364.

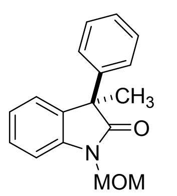

4d

(±)-1-(methoxymethyl)-3-methyl-3-phenylindolin-2-one (4d)

Prepared according to the general procedure using iodobenzene **1a** (20.4 mg, 0. 1 mmol), 1-(methoxymethyl)-3-methylindolin-2-one (38.2 mg, 0.2 mmol), $CsOH\cdot H_2O$ (50.4 mg, 0.3 mmol), and CH_3CN (1 mL). Time of irradiation: 6 hours. The crude mixture was purified by silica gel chromatography (acetone/petroleum ether 1:5) to afford the product **4d** as a colorless oil (20.3 mg, 76% yield). ^{1}H NMR (400 MHz, $CDCl_3$): δ 7.36 – 7.22 (m, 6H), 7.20 (d, J = 6.8, 1H), 7.14 – 7.11 (m, 2H), 5.20 – 5.12 (m, 2H), 3.31 (s, 3H), 1.83 (s, 3H); ^{13}C NMR (101 MHz, $CDCl_3$): δ 180.1, 141.4, 140.7, 134.4, 128.6, 128.3, 127.4, 126.6, 124.4, 123.4, 109.8, 71.5, 56.2, 52.5, 24.0; HR-ESI-MS (*m/z*): calcd. for $C_{17}H_{17}NO_2Na$ $[M + Na]^+$, 290.1151, found 290.1152.

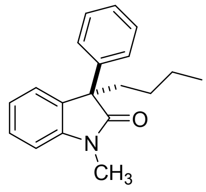

4e

(±)-3-butyl-1-methyl-3-phenylindolin-2-one (4e)

Prepared according to the general procedure using iodobenzene **1a** (20.4 mg, 0. 1 mmol), 3-butyl-1-methylindolin-2-one (40.6 mg, 0.2 mmol), $CsOH\cdot H_2O$ (50.4 mg, 0.3 mmol), and CH_3CN (1 mL). Time of irradiation: 6 hours. The crude mixture was purified by silica gel chromatography (acetone/petroleum ether 1:5) to afford the product **4e** as a colorless oil (22.9 mg, 82% yield). ^{1}H NMR (400 MHz, $CDCl_3$): δ 7.39 – 7.22 (m, 7H), 7.12 (t, J = 7.5, 1H), 6.90 (d, J = 6.8, 1H), 3.23 (s, 3H), 2.37 (td, J = 12.8, 4.5, 1H), 2.19 (td, J = 12.9, 4.1, 1H), 1.36 – 1.17 (m, 2H), 1.17 – 0.98 (m, 1H), 0.93 – 0.71 (m, 4H); ^{13}C NMR (100 MHz, $CDCl_3$): δ 178.7, 144.0, 140.4, 132.5, 128.5, 128.1, 127.2, 126.9, 124.8, 122.6, 108.2, 56.7, 37.8, 26.7, 26.4, 22.8, 13.8; HR-

ESI-MS (*m/z*): calcd. for $C_{19}H_{21}NONa$ [M + Na]$^+$, 302.1515, found 302.1519.

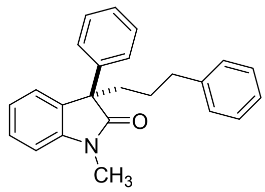

4f

(±)-1-methyl-3-phenyl-3-(3-phenylpropyl)indolin-2-one (4f)

Prepared according to the general procedure using iodobenzene **1a** (20.4 mg, 0. 1 mmol), 1-methyl-3-(3-phenylpropyl)indolin-2-one (53 mg, 0.2 mmol), CsOH·H_2O (50.4 mg, 0.3 mmol), and CH_3CN (1 mL). Time of irradiation: 6 hours. The crude mixture was purified by silica gel chromatography (acetone/petroleum ether 1:5) to afford the product **4f** as a colorless oil (27.3 mg, 80% yield). ^{1}H NMR (400 MHz, $CDCl_3$): δ 7.37 – 7.26 (m, 5H), 7.25 – 7.14 (m, 5H), 7.13 – 7.03 (m, 3H), 6.88 (d, *J* = 7.8, 1H), 3.21 (s, 3H), 2.61 (ddd, *J* = 14.9, 9.3, 5.9, 1H), 2.57 – 2.48 (m, 1H), 2.48 – 2.38 (m, 1H), 2.23 (td, *J* = 12.9, 4.0, 1H), 1.53 – 1.39 (m, 1H), 1.38 – 1.13 (m, 1H); ^{13}C NMR (100 MHz, $CDCl_3$): δ 178.6, 143.9, 141.8, 140.2, 132.1, 128.5, 128.4, 128.3, 128.2, 127.2, 126.9, 125.8, 124.8, 122.6, 108.3, 56.6, 37.6, 36.0, 26.4; HR-ESI-MS (*m/z*): calcd. for $C_{24}H_{23}NONa$ [M + Na]$^+$, 364.1672, found 364.1681.

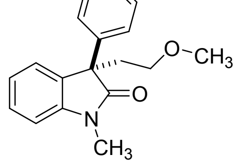

4g

(±)-3-(2-methoxyethyl)-1-methyl-3-phenylindolin-2-one (4g)

Prepared according to the general procedure using iodobenzene **1a** (20.4 mg, 0. 1 mmol), 3-(2-methoxyethyl)-1-methylindolin-2-one (41 mg, 0.2 mmol), CsOH·H_2O (50.4 mg, 0.3 mmol), and CH_3CN (1 mL). Time of irradiation: 8 hours. The crude mixture was purified by silica gel chromatography (acetone/petroleum ether 1:5) to afford the product **4g** as a colorless oil (22.5 mg, 80% yield). ^{1}H NMR (400 MHz,

CDCl₃): δ 7.38 – 7.21 (m, 7H), 7.12 (t, *J* = 7.5, 1H), 6.91 (d, *J* = 7.8, 1H), 3.22 (s, 3H), 3.19 – 3.12 (m, 2H), 3.11 (s, 3H), 2.84 (dt, *J* = 13.5, 7.9, 1H), 2.40 (ddd, *J* = 13.3, 7.4, 4.6, 1H); ^{13}C NMR (100 MHz, $CDCl_3$): δ 178.5, 144.1, 140.2, 131.5, 128.6, 128.3, 127.3, 126.7, 125.0, 122.5, 108.3, 68.9, 58.6, 54.5, 37.0, 26.5; HR-ESI-MS (*m/z*): calcd. for $C_{18}H_{19}NO_2Na$ $[M + Na]^+$, 304.1308, found 304.1311.

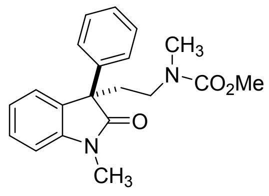

4h

Methyl (±)-methyl(2-(1-methyl-2-oxo-3-phenylindolin-3-yl)ethyl)carbamate (4h)

Prepared according to the general procedure using iodobenzene **1a** (20.4 mg, 0. 1 mmol), methyl methyl(2-(1-methyl-2-oxoindolin-3-yl)ethyl)carbamate (52.4 mg, 0.2 mmol), $CsOH·H_2O$ (50.4 mg, 0.3 mmol), and CH_3CN (1 mL). Time of irradiation: 8 hours. The crude mixture was purified by silica gel chromatography (acetone/petroleum ether 1:5) to afford the product **4h** as a colorless oil (24.7 mg, 73% yield). ^{1}H NMR (400 MHz, $CDCl_3$): (rotameric mixture)[4] δ = 7.44 – 7.20 (m, 7H), 7.15 (t, *J* = 7.4, 1H), 6.92 (d, *J* = 6.7, 1H), 3.60 (brs, 1.4H), 3.58 (brs, 1.6H), 3.23 (s, 3H), 3.10 – 2.90 (m, 2H), 2.81 (brs, 1.6H), 2.78 (brs, 1.4H), 2.71 – 2.54 (m, 1H), 2.54 – 2.30 (m, 1H); ^{13}C NMR (100 MHz, $CDCl_3$): (rotameric mixture) δ 177.8, 156.6, 143.7, 139.7, 131.3, 128.6, 128.5, 127.5, 126.7, 124.7, 122.8, 108.5, 54.9, 52.6, 45.5, 45.0, 35.3, 34.7, 34.1, 26.5; HR-ESI-MS (*m/z*): calcd. for $C_{20}H_{22}N_2O_3Na$ $[M + Na]^+$, 361.1523, found 361.1526.

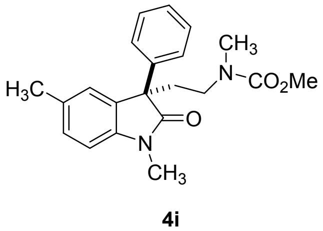

4i

Methyl (±)-(2-(1,5-dimethyl-2-oxo-3-phenylindolin-3-yl)ethyl)(methyl)carbamate

(4i)

Prepared according to the general procedure using iodobenzene **1a** (20.4 mg, 0. 1 mmol), methyl (2-(1,5-dimethyl-2-oxoindolin-3-yl)ethyl)(methyl)carbamate (55.2 mg, 0.2 mmol), $CsOH·H_2O$ (50.4 mg, 0.3 mmol), and CH_3CN (1 mL). Time of irradiation: 8 hours. The crude mixture was purified by silica gel chromatography (acetone/petroleum ether 1:5) to afford the product **4i** as a colorless oil (26.4 mg, 75% yield). ^{1}H NMR (600 MHz, $CDCl_3$): (rotameric mixture)[1] δ 7.44 – 7.19 (m, 5H), 7.19 – 7.01 (m, 2H), 6.81 – 6.77 (m, 1H), 3.60 (brs, 3H), 3.21 (brs, 3H), 3.06-2.97 (m, 2H), 2.81 (brs, 1.5H), 2.78 (brs, 1.5H), 2.68-2.59 (m, 1H), 2.54 – 2.27 (m, 4H); ^{13}C NMR (150 MHz, $CDCl_3$): (rotameric mixture) δ 156.7, 147.2, 141.3, 139.9, 132.3, 131.5, 128.6, 127.4, 126.7, 125.5, 108.1, 52.5, 45.0, 35.2, 34.5, 26.5, 21.2; HR-ESI-MS (*m/z*): calcd. for $C_{21}H_{24}N_2O_3Na$ $[M + Na]^+$, 375.1679, found 375.1682.

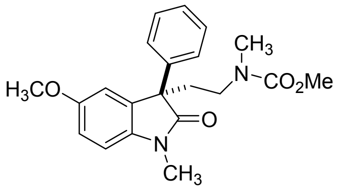

4j

Methyl (±)-(2-(5-methoxy-1-methyl-2-oxo-3-phenylindolin-3-yl)ethyl)(methyl)carbamate (4j)

Prepared according to the general procedure using iodobenzene **1a** (20.4 mg, 0. 1 mmol), methyl (2-(5-methoxy-1-methyl-2-oxoindolin-3-yl)ethyl)(methyl)carbamate (58.4 mg, 0.2 mmol), $CsOH·H_2O$ (50.4 mg, 0.3 mmol), and CH_3CN (1 mL). Time of irradiation: 8 hours. The crude mixture was purified by silica gel chromatography (acetone/petroleum ether 1:5) to afford the product **4j** as a colorless oil (23.2 mg, 68% yield). ^{1}H NMR (400 MHz, $CDCl_3$): (rotameric mixture) δ 7.46 – 7.18 (m, 5H), 6.92-6.85 (m, 3H), 3.81 (brs, 3H), 3.61 (brs, 3H), 3.20 (brs, 3H), 3.04 – 2.94 (m, 2H), 2.82 (brs, 1.8H), 2.78 (brs, 1.2H),2.73 – 2.60 (m, 1H), 2.50 – 2.30 (m, 1H); ^{13}C NMR (100 MHz, $CDCl_3$): (rotameric mixture) δ 177.5, 156.6, 156.2, 139.7, 137.3, 132.8, 128.6, 127.4, 126.7, 112.6, 112.3, 108.7, 55.9, 55.3, 52.6, 45.5, 45.0, 35.2, 34.5, 34.1, 26.5;

HR-ESI-MS (*m/z*): calcd. for $C_{21}H_{24}N_2O_4Na$ $[M + Na]^+$, 391.1628, found 391.1635.

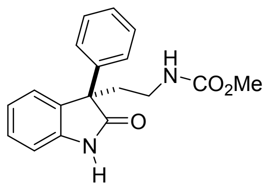

4k

Methyl (±)-(2-(2-oxo-3-phenylindolin-3-yl)ethyl)carbamate (4k)

Prepared according to the general procedure using iodobenzene **1a** (20.4 mg, 0. 1 mmol), methyl (2-(2-oxoindolin-3-yl)ethyl)carbamate (46.8 mg, 0.2 mmol), $CsOH{\cdot}H_2O$ (50.4 mg, 0.3 mmol), and DMSO (1 mL). Time of irradiation: 10 hours. The crude mixture was purified by silica gel chromatography (acetone/petroleum ether 1:3) to afford the product **4k** as a colorless oil (14.3 mg, 46% yield). ^{1}H NMR (600 MHz, $CDCl_3$): δ 8.16 (s, 1H), 7.36 (d, *J* = 7.5, 2H), 7.33 – 7.28 (m, 3H), 7.28 – 7.23 (m, 2H), 7.11 (t, *J* = 7.4, 1H), 6.95 (d, *J* = 7.6, 1H), 4.77 (s, 1H), 3.57 (s, 3H), 3.12 – 3.02 (m, 2H), 2.71 – 2.64 (m, 1H), 2.49 – 2.43 (m, 1H); ^{13}C NMR (150 MHz, $CDCl_3$): δ 180.8, 156.8, 140.8, 139.6, 132.1, 128.7, 128.5, 127.5, 126.7, 125.1, 123.0, 110.4, 55.7, 52.0, 37.5, 37.1; HR-ESI-MS (*m/z*): calcd. for $C_{18}H_{18}N_2O_3Na$ $[M + Na]^+$, 333.1210, found 333.1211.

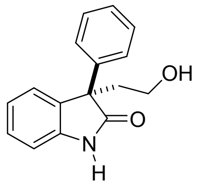

4l

(±)-3-(2-hydroxyethyl)-3-phenylindolin-2-one (4l)

Prepared according to the general procedure using iodobenzene **1a** (20.4 mg, 0. 1 mmol), 3-(2-hydroxyethyl)indolin-2-one (35.4 mg, 0.2 mmol), $CsOH{\cdot}H_2O$ (50.4 mg, 0.3 mmol), and DMSO (1 mL). Time of irradiation: 10 hours. The crude mixture was purified by silica gel chromatography (acetone/petroleum ether 1:2.5) to afford the product **4l** as a colorless oil (12.1 mg, 48% yield). ^{1}H NMR (600 MHz, $CDCl_3$): δ

9.02 (s, 1H), 7.36 – 7.32 (m, 2H), 7.31 – 7.27 (m, 2H), 7.24 (d, *J* = 8.3, 1H), 7.20 (td, *J* = 7.7, 1.0, 1H), 7.15 (d, *J* = 7.4, 1H), 7.08-7.04 (m, 1H), 6.89 (d, *J* = 7.7, 1H), 3.58 – 3.52 (m, 1H), 3.46 – 3.40 (m, 1H), 2.84 – 2.80 (m, 1H), 2.42 – 2.38 (m, 1H); ^{13}C NMR (150 MHz, $CDCl_3$): δ 181.8, 141.2, 140.1, 132.5, 128.7, 128.4, 127.4, 126.7, 124.9, 122.7, 110.5, 59.3, 55.5, 39.8; HR-ESI-MS (*m/z*): calcd. for $C_{16}H_{16}NO_2$ [M + H]$^+$, 254.1176, found 254.1175.

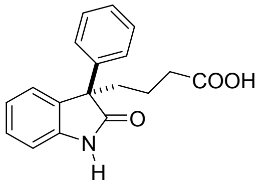

4m

(±)- 4-(2-oxo-3-phenylindolin-3-yl)butanoic acid (4m)

Prepared according to the general procedure using iodobenzene **1a** (20.4 mg, 0. 1 mmol), 4-(2-oxoindolin-3-yl)butanoic acid (43.8 mg, 0.2 mmol), $CsOH{\cdot}H_2O$ (67.2 mg, 0.4 mmol), and DMSO (1 mL). Time of irradiation: 10 hours. The crude mixture was purified by silica gel chromatography (acetone/petroleum ether 1:2) to afford the product **4m** as a colorless oil (11.5 mg, 39% yield). ^{1}H NMR (600 MHz, $CDCl_3$): δ 8.68 (s, 1H), 7.36 – 7.33 (m, 2H), 7.32 – 7.28 (m, 2H), 7.27 – 7.22 (m, 2H), 7.20 (d, *J* = 7.4, 1H), 7.10 (d, *J* = 7.5, 1H), 6.97 (d, *J* = 7.7, 1H), 2.47 – 2.41 (m, 1H), 2.35 – 2.25 (m, 3H), 1.57 – 1.53 (m, 1H), 1.36 – 1.32 (m, 1H); ^{13}C NMR (151 MHz, $CDCl_3$): δ 140.8, 139.6, 132.4, 128.6, 128.4, 127.5, 126.8, 125.1, 122.9, 110.3, 57.0, 36.8, 33.7, 20.0; HR-ESI-MS (*m/z*): calcd. for $C_{18}H_{17}NO_3Na$ [M + Na]$^+$, 318.1101, found 318.1102.

References

[1] Liu, B.; Lim, C.-H.; Miyake, G. M., *J. Am. Chem. Soc.* **2017**, *139*, 13616-13619.

[2] Montalti, M. Credi, A.; Prodi, L.; Gandolfi, M. *Handbook of Photochemistry*, 3rd ed.; CRC/Taylor & Francis: Boca Raton, FL, 2006.

[3] Demas, J.; Bowman, W.; Zalewski, E.; Velapoldi, R. *J. Phys. Chem.* **1981**, *85*, 2766-2771.

[4] De, S.; Das, M. K.; Bhunia, S.; Bisai, A. r. *Org. Lett.* **2015**, *17*, 5922-5925.

NMR Spectral Data

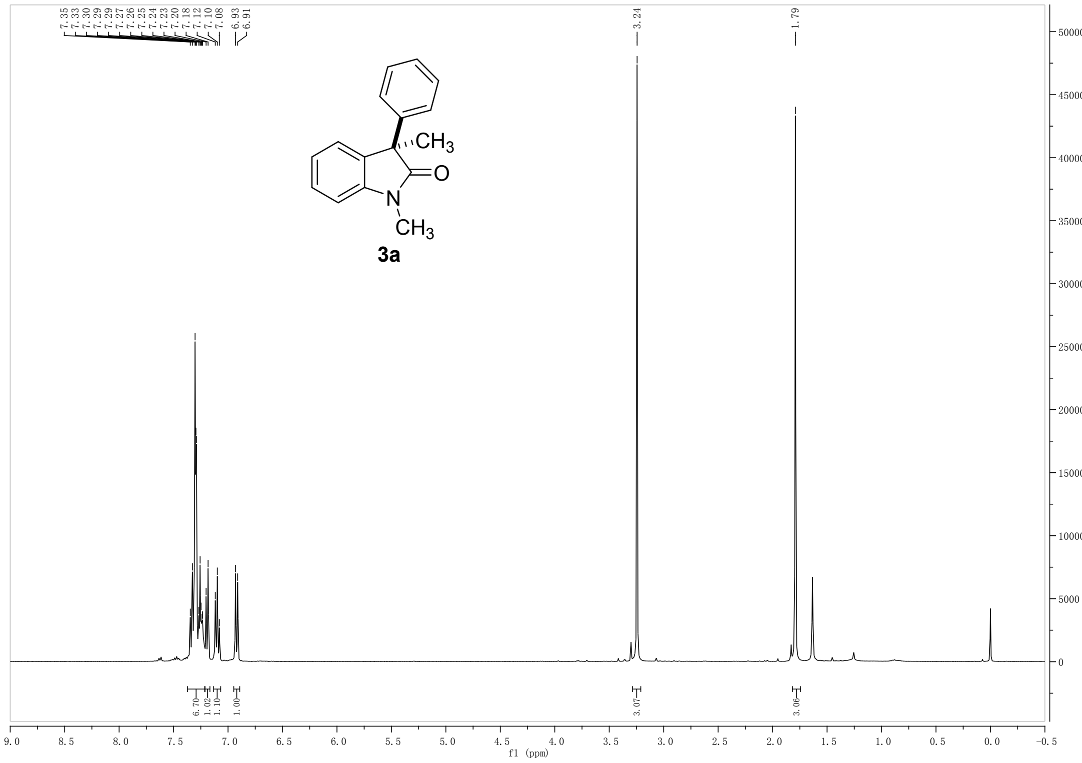

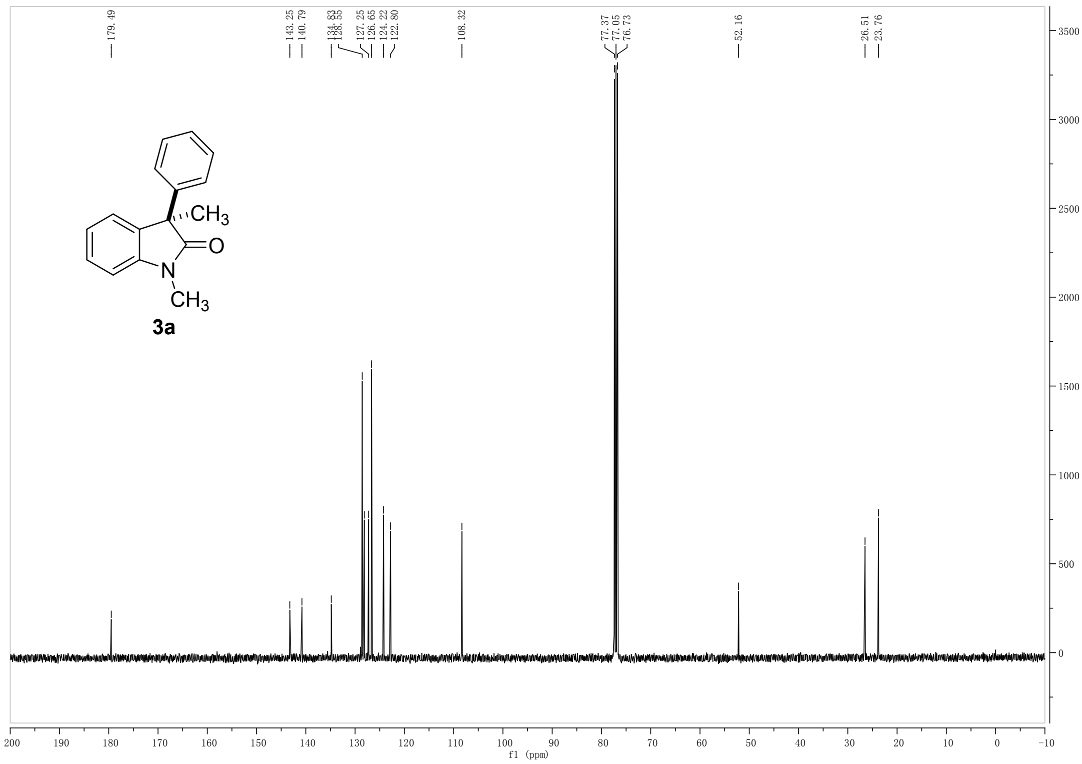

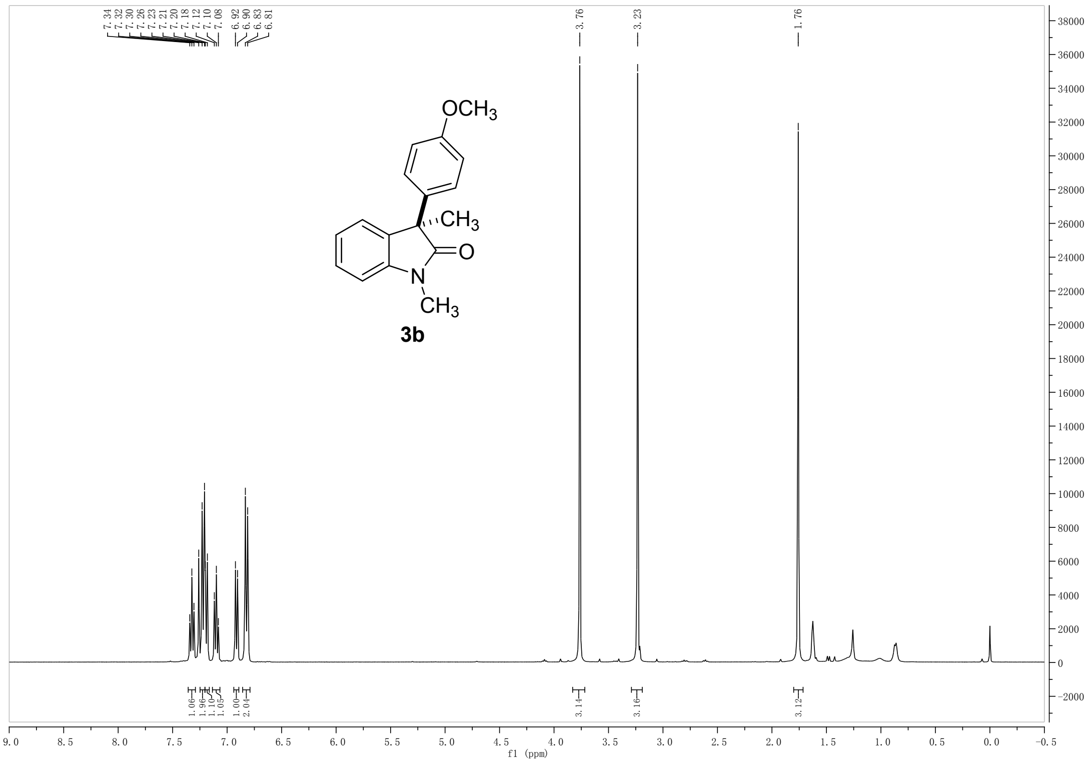

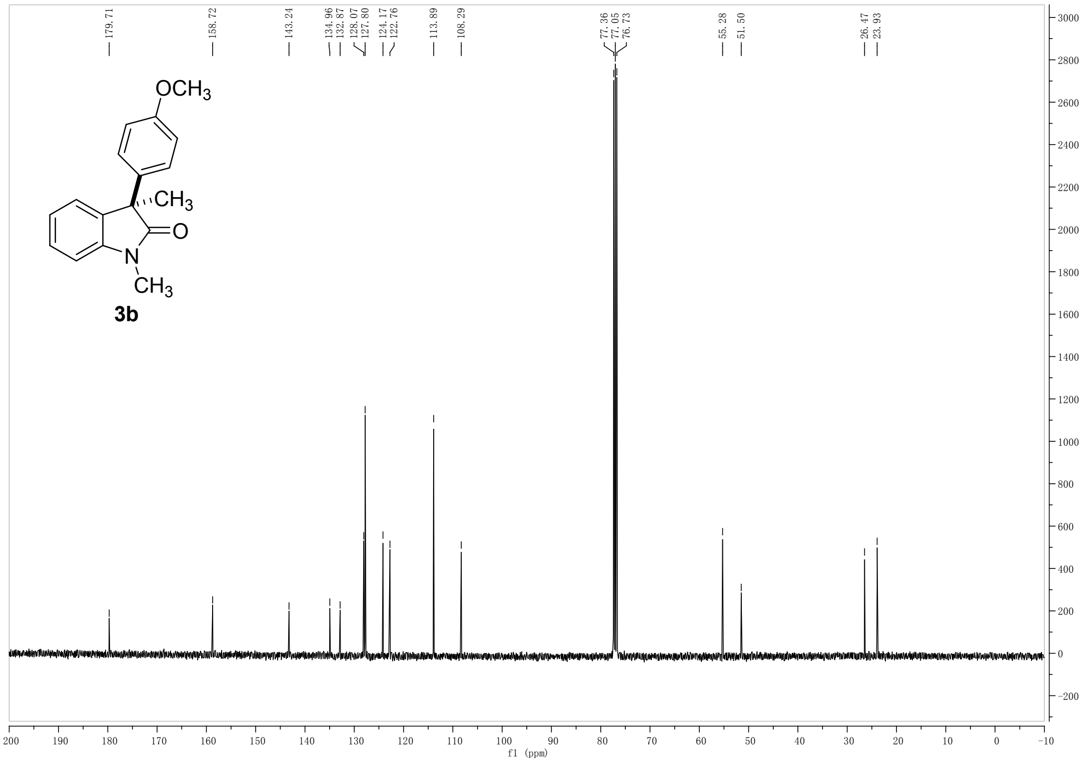

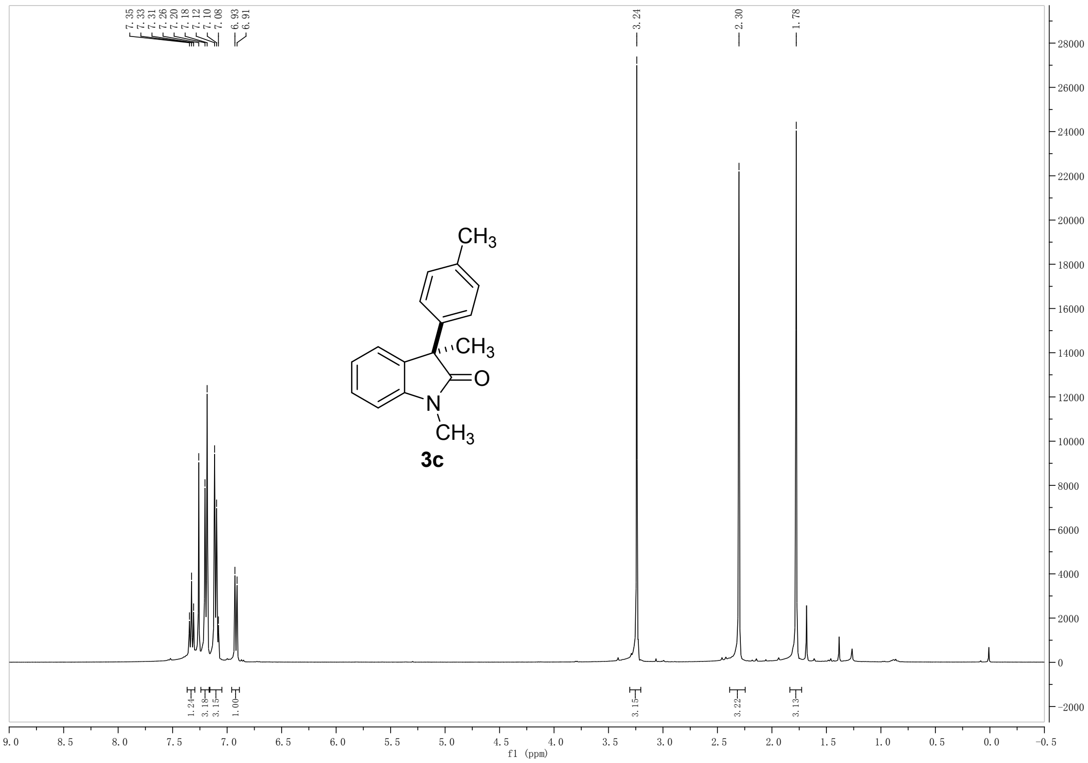
7.35
7.33
7.31
7.26
7.20
7.18
7.12
7.10
7.08
6.93
6.91
3.24
2.30
1.78
CH3
CH3
O
N
CH3
3c
1.24
3.18
3.15
1.00
3.15
3.22
3.13
f1 (ppm)

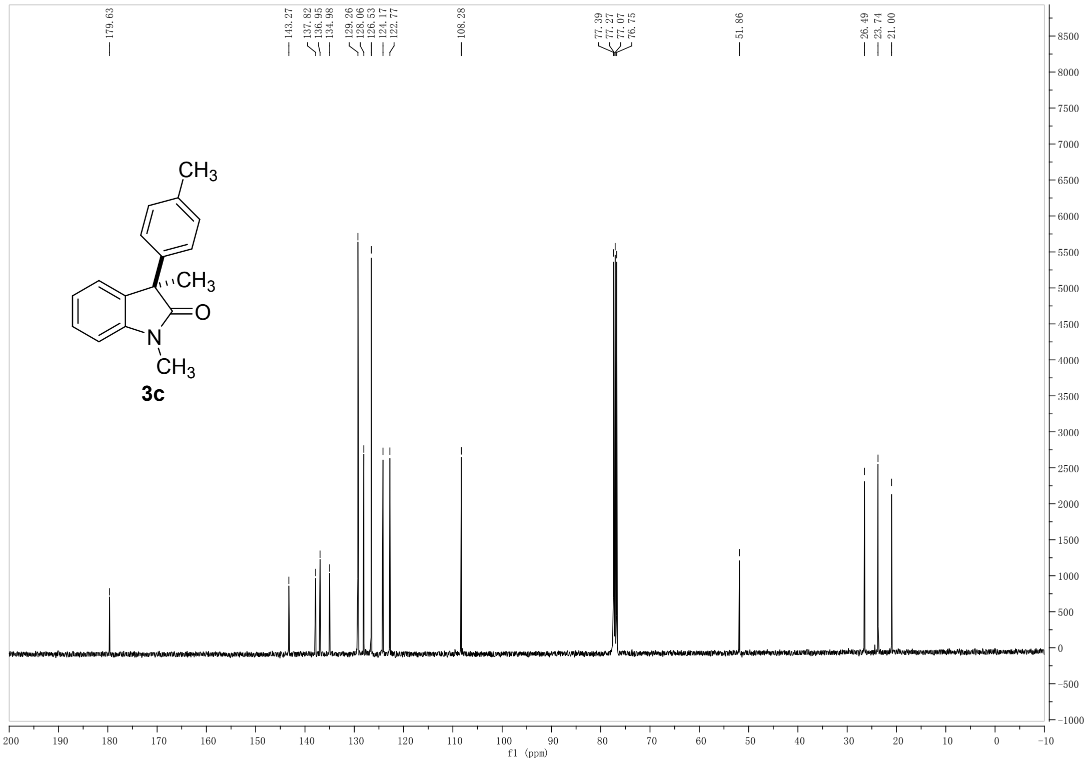
179.63
143.27
137.82
136.95
134.98
129.26
128.06
126.53
124.17
122.77
108.28
77.39
77.27
77.07
76.75
51.86
26.49
23.74
21.00
CH3
CH3
O
N
CH3
3c
f1 (ppm)

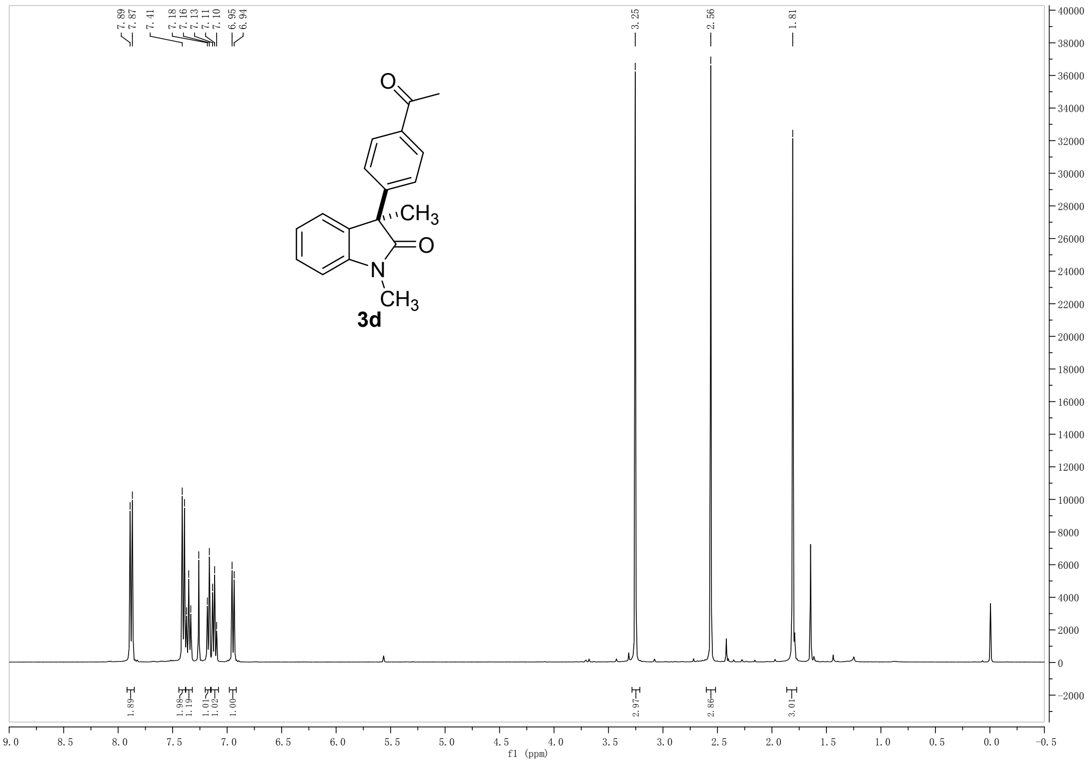
7.89
7.87
7.41
7.18
7.16
7.13
7.11
7.10
6.95
6.94
3.25
2.56
1.81
CH3
O
N
CH3
3d
1.89
1.98
1.19
1.01
1.02
1.00
2.97
2.86
3.01
f1 (ppm)

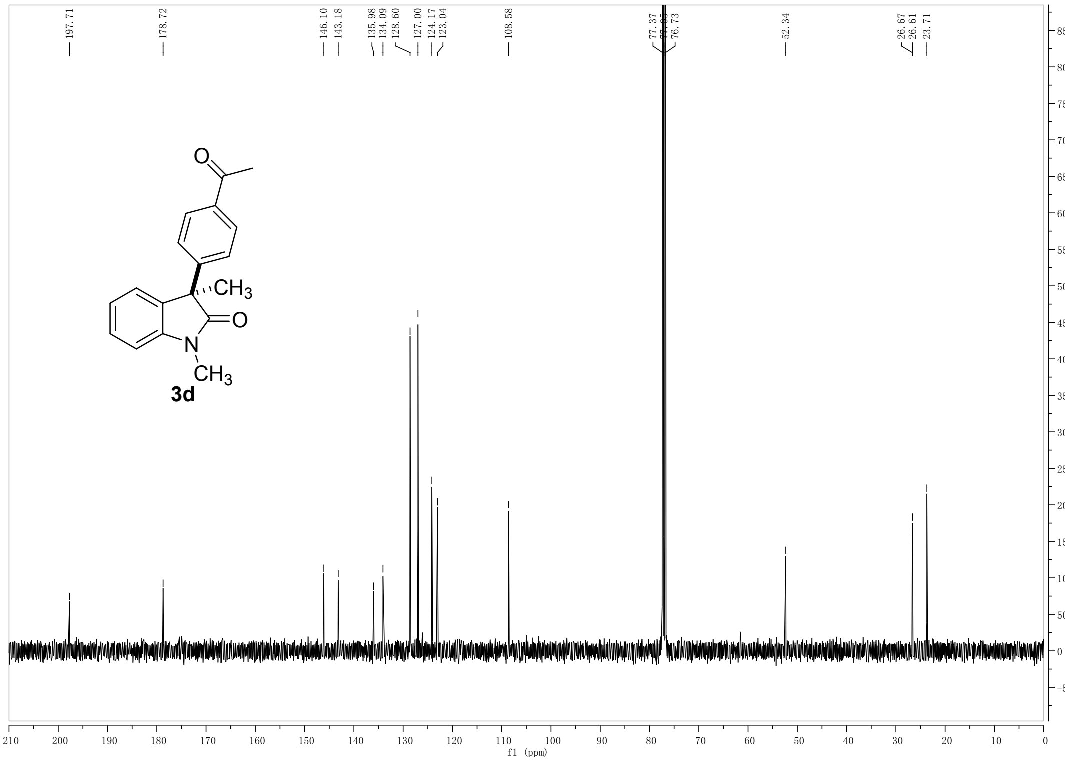
197.71
178.72
146.10
143.18
135.98
134.09
128.60
127.00
124.17
123.04
108.58
77.37
76.73
52.34
26.67
26.61
23.71
CH3
O
N
CH3
3d
f1 (ppm)

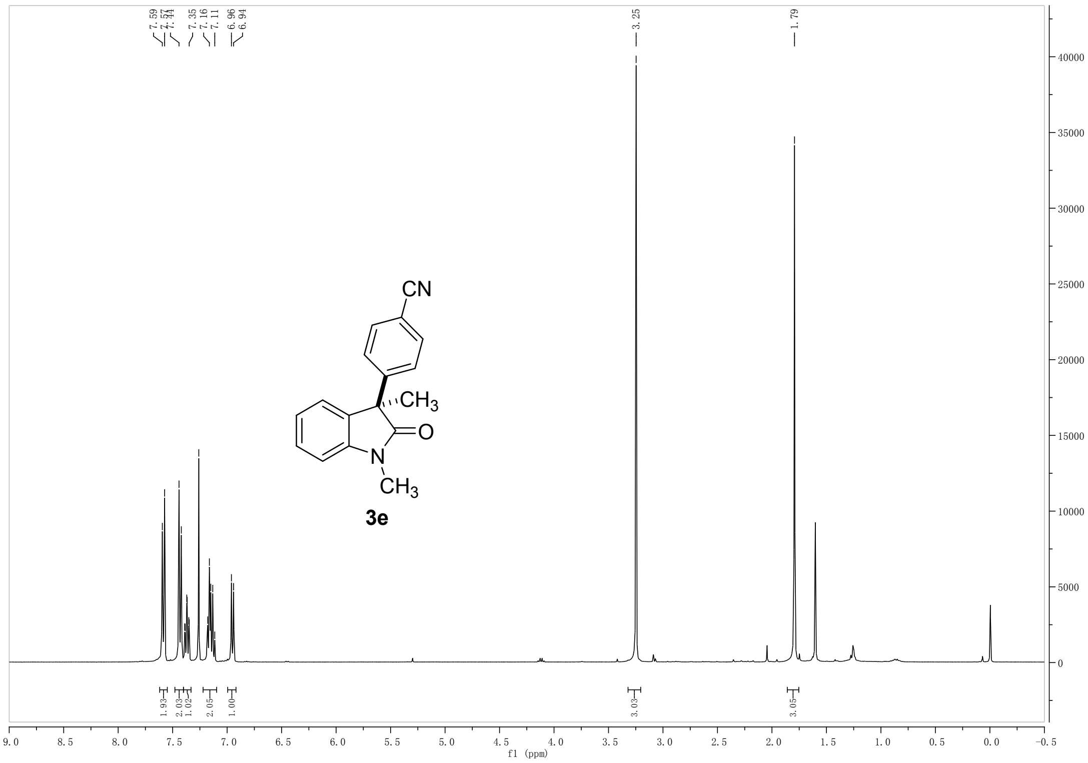
7.59
7.57
7.44
7.35
7.16
7.11
6.96
6.94
3.25
1.79
1.93
2.03
1.02
2.05
1.00
3.03
3.05
CN
CH3
O
N
CH3
3e
f1 (ppm)

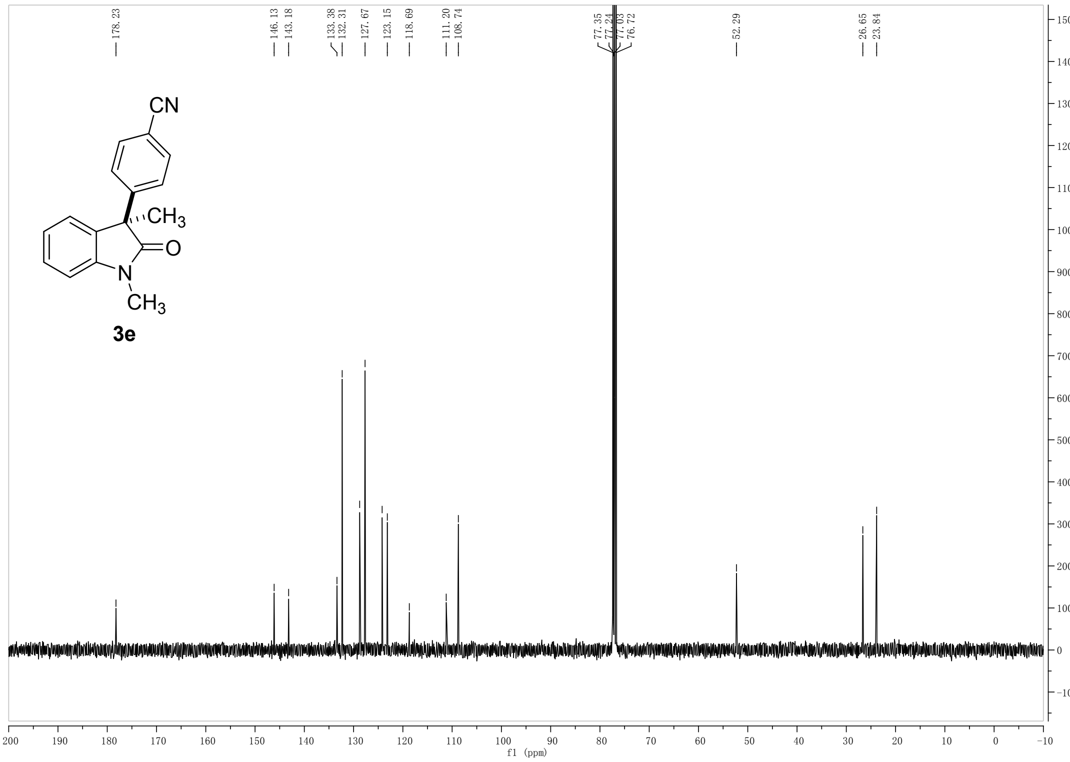
178.23
146.13
143.18
133.38
132.31
127.67
123.15
118.69
111.20
108.74
77.35
76.72
52.29
26.65
23.84
CN
CH3
O
N
CH3
3e
f1 (ppm)

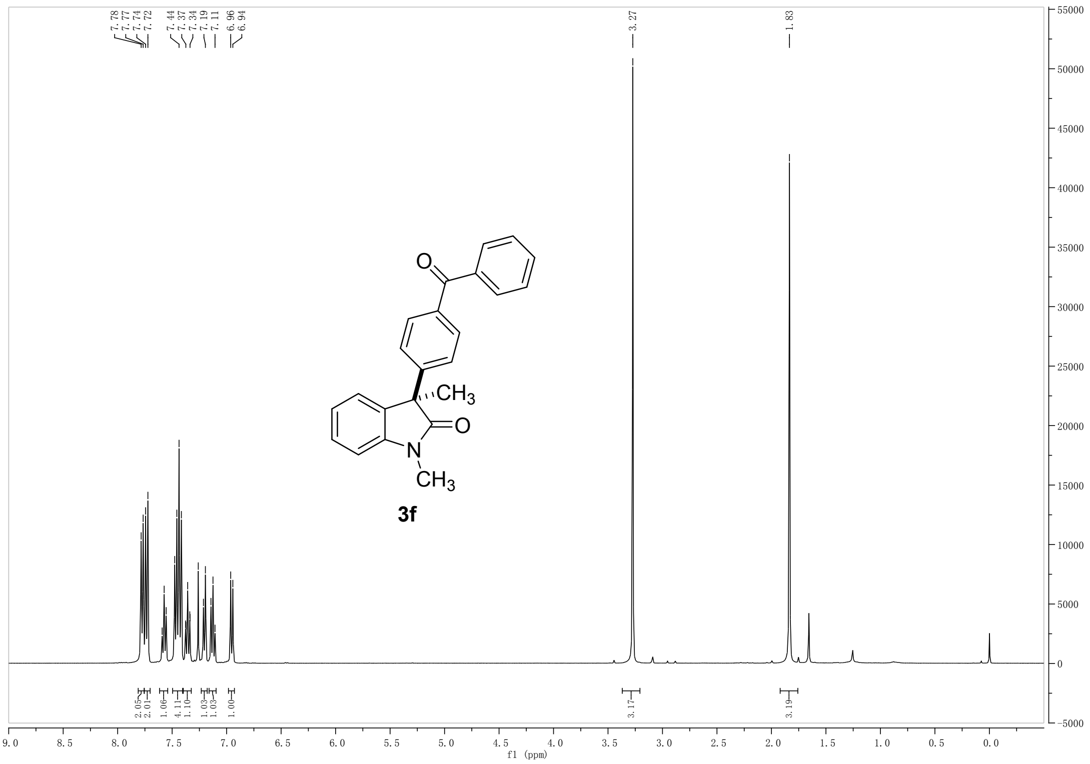
7.78
7.77
7.74
7.72
7.44
7.37
7.34
7.19
7.11
6.96
6.94
3.27
1.83
2.05
2.01
1.06
4.11
1.10
1.03
1.03
1.00
3.17
3.19
CH3
O
N
CH3
3f
f1 (ppm)

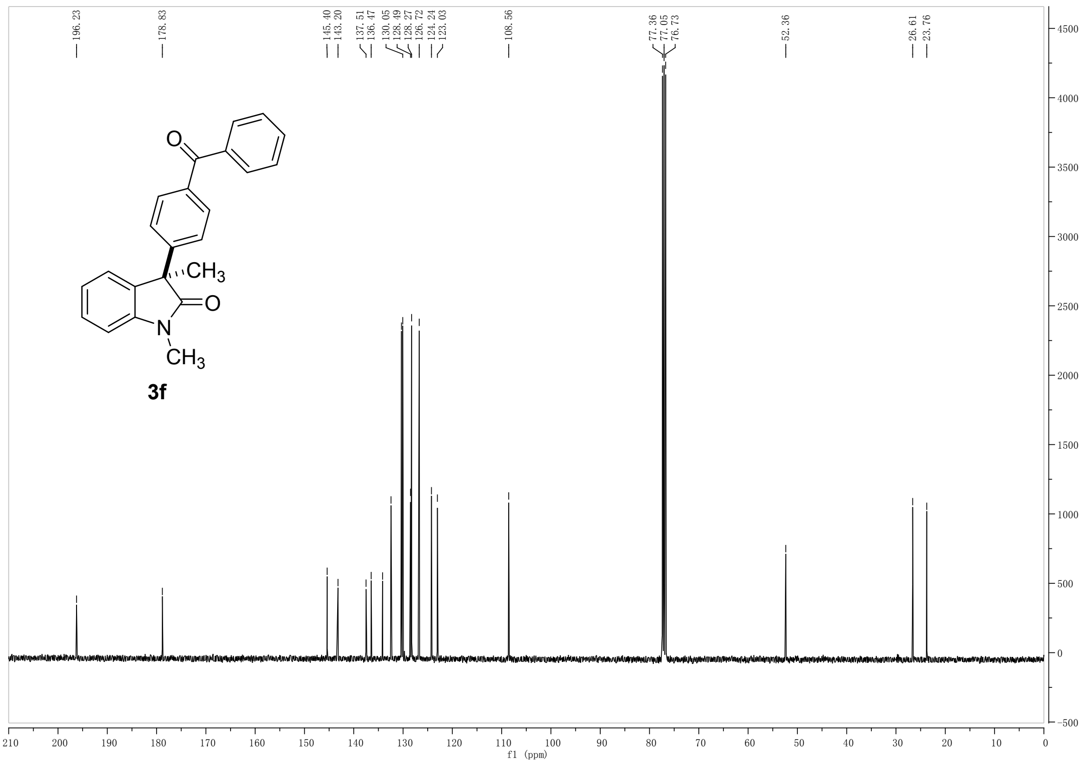
196.23
178.83
145.40
143.20
137.51
136.47
130.05
128.49
128.27
126.72
124.24
123.03
108.56
77.36
77.05
76.73
52.36
26.61
23.76
CH3
O
N
CH3
3f
f1 (ppm)

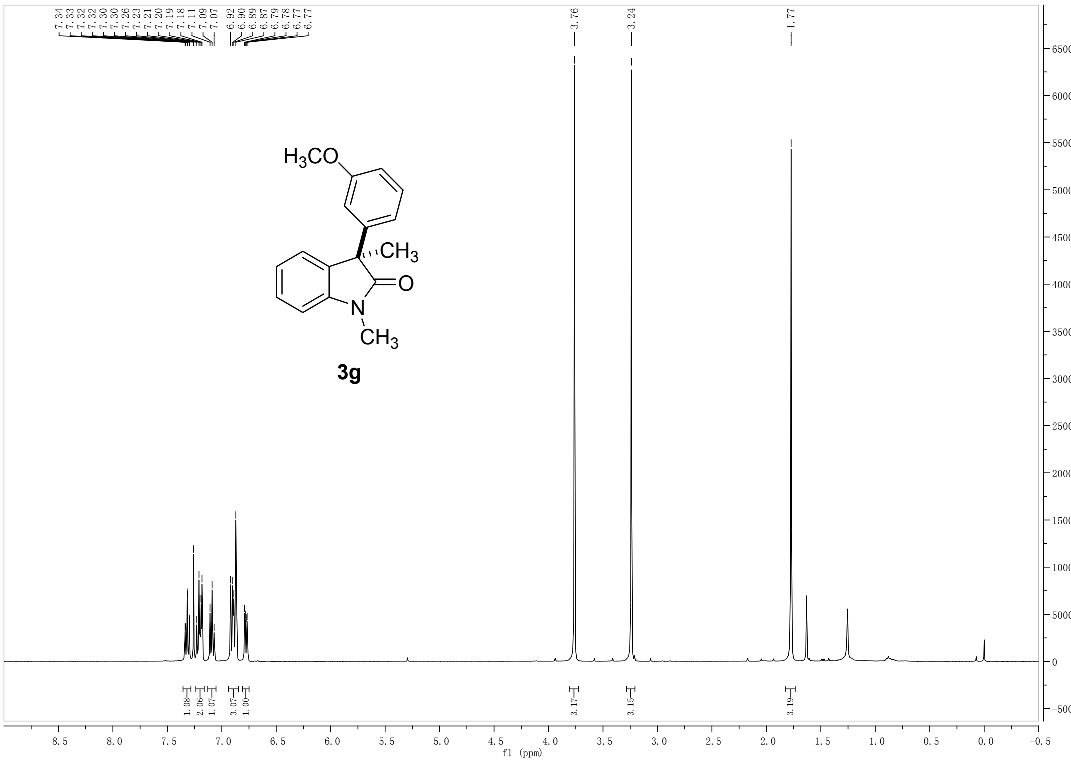
7.34
7.33
7.32
7.32
7.30
7.30
7.26
7.23
7.21
7.20
7.19
7.18
7.11
7.09
7.07
6.92
6.90
6.89
6.87
6.79
6.78
6.77
6.77
3.76
3.24
1.77
H3CO
CH3
O
N
CH3
3g
1.08
2.06
1.07
3.07
1.00
3.17
3.15
3.19
f1 (ppm)

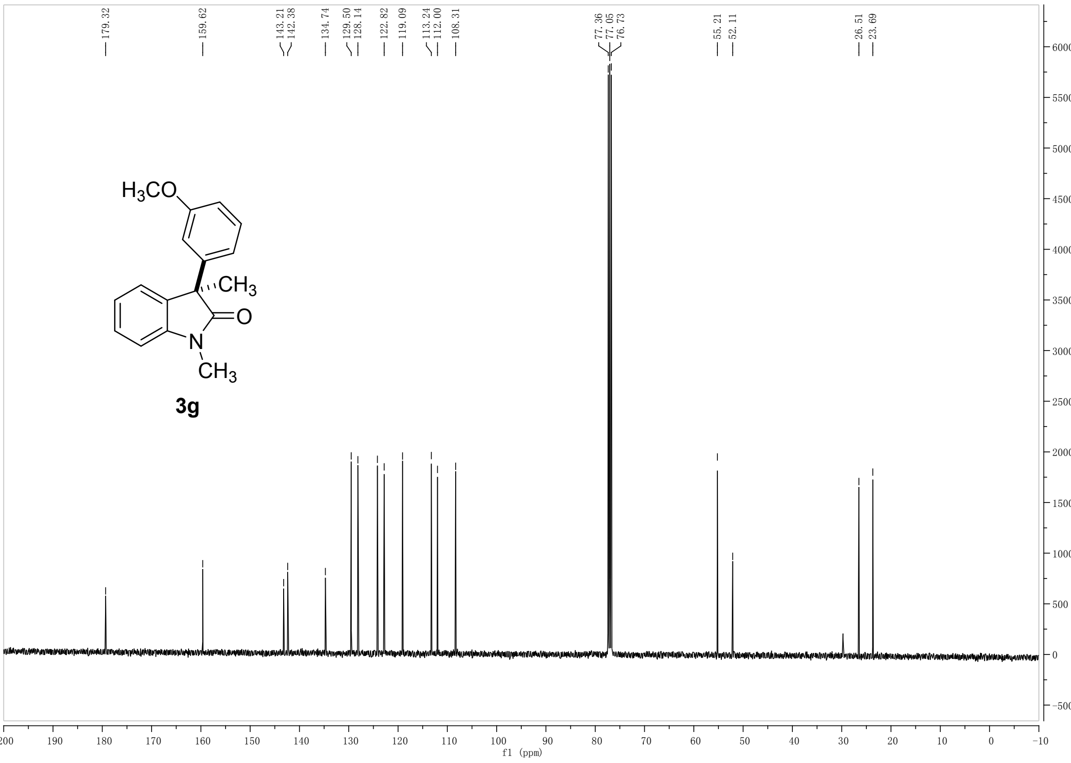
179.32
159.62
143.21
142.38
134.74
129.50
128.14
122.82
119.09
113.24
112.00
108.31
77.36
77.05
76.73
55.21
52.11
26.51
23.69
H3CO
CH3
O
N
CH3
3g
f1 (ppm)

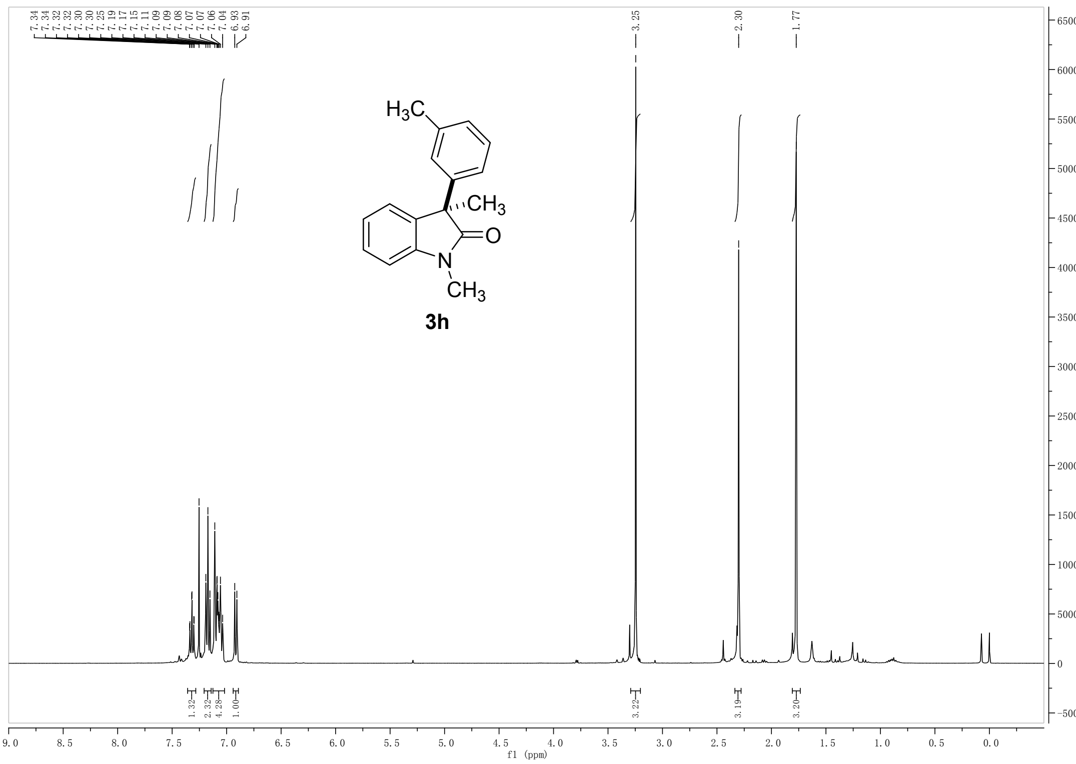

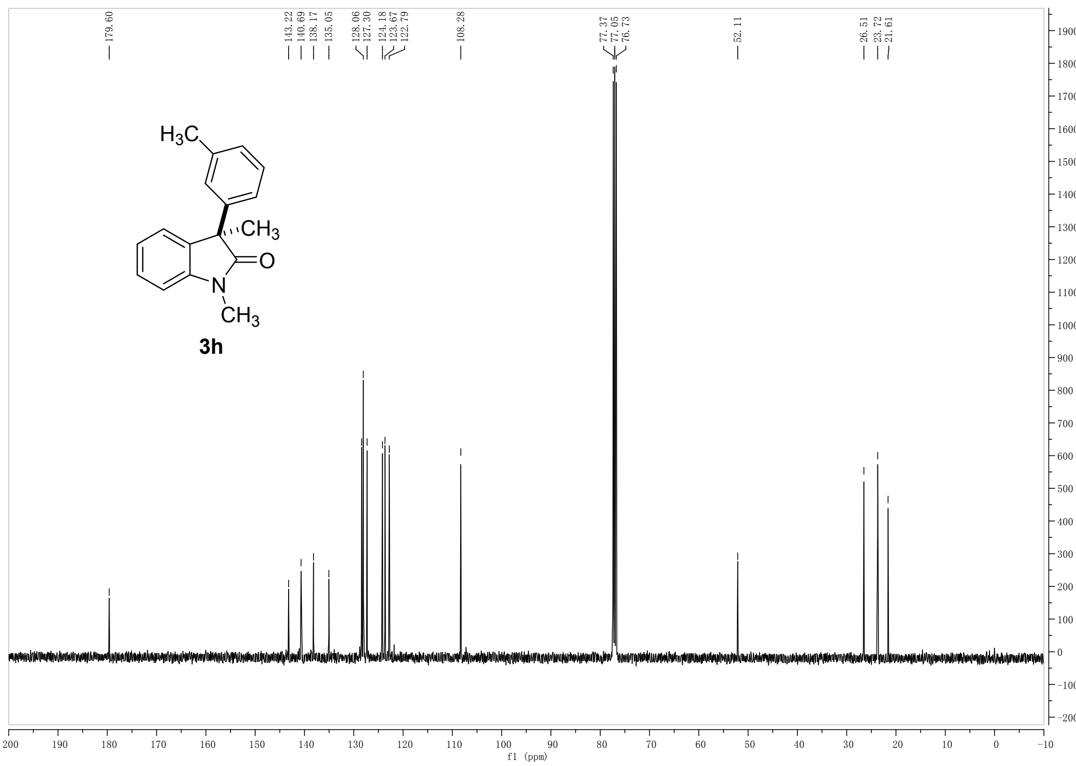

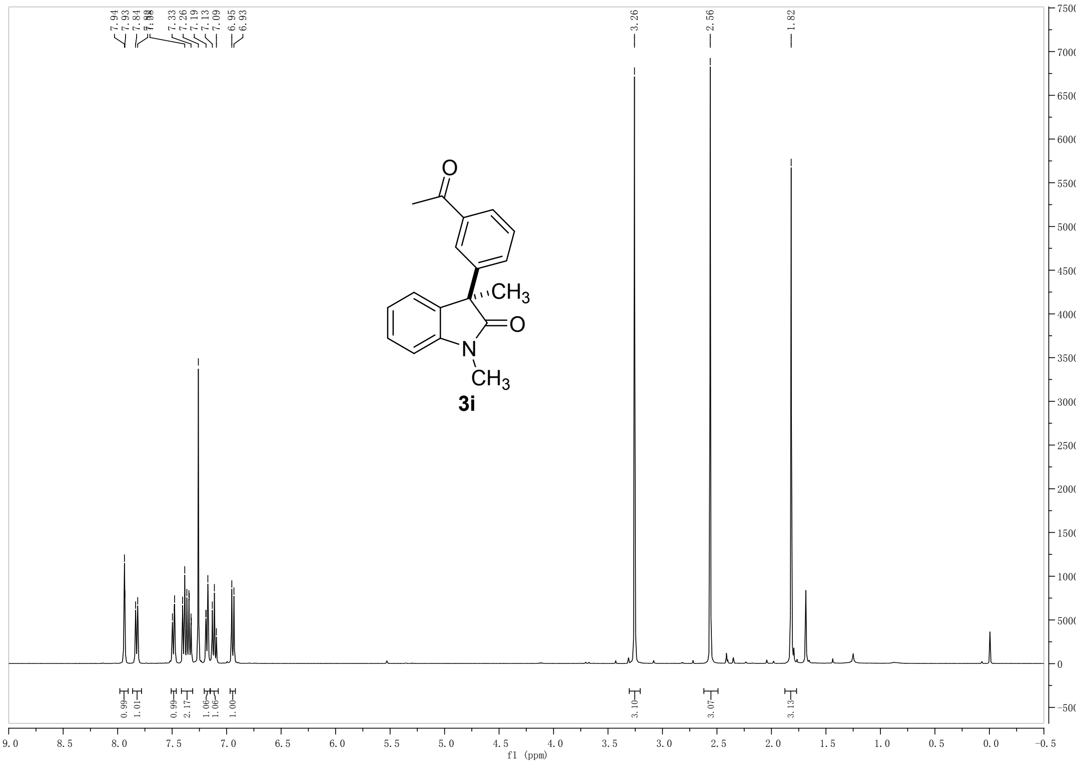
7.94
7.93
7.84
7.33
7.26
7.19
7.13
7.09
6.95
6.93
3.26
2.56
1.82
0.99
1.01
0.99
2.17
1.06
1.06
1.00
3.10
3.07
3.13
3i
f1 (ppm)

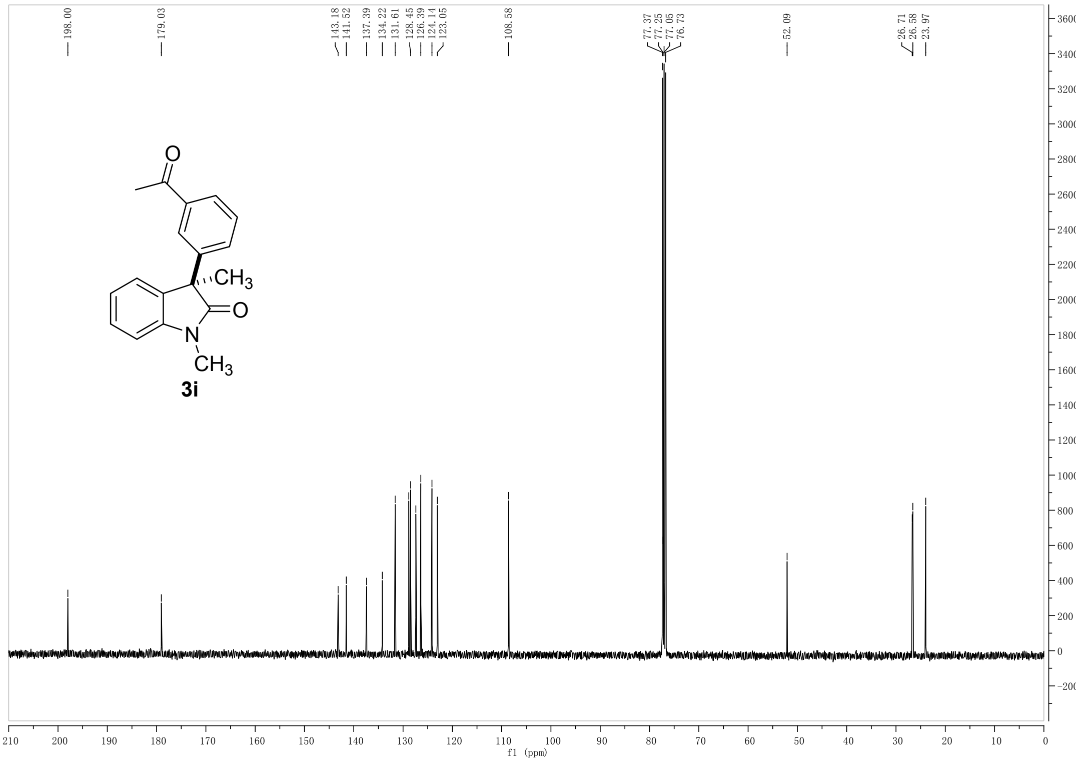
198.00
179.03
143.18
141.52
137.39
134.22
131.61
128.45
126.39
124.14
123.05
108.58
77.37
77.25
77.05
76.73
52.09
26.71
26.58
23.97
3i
f1 (ppm)

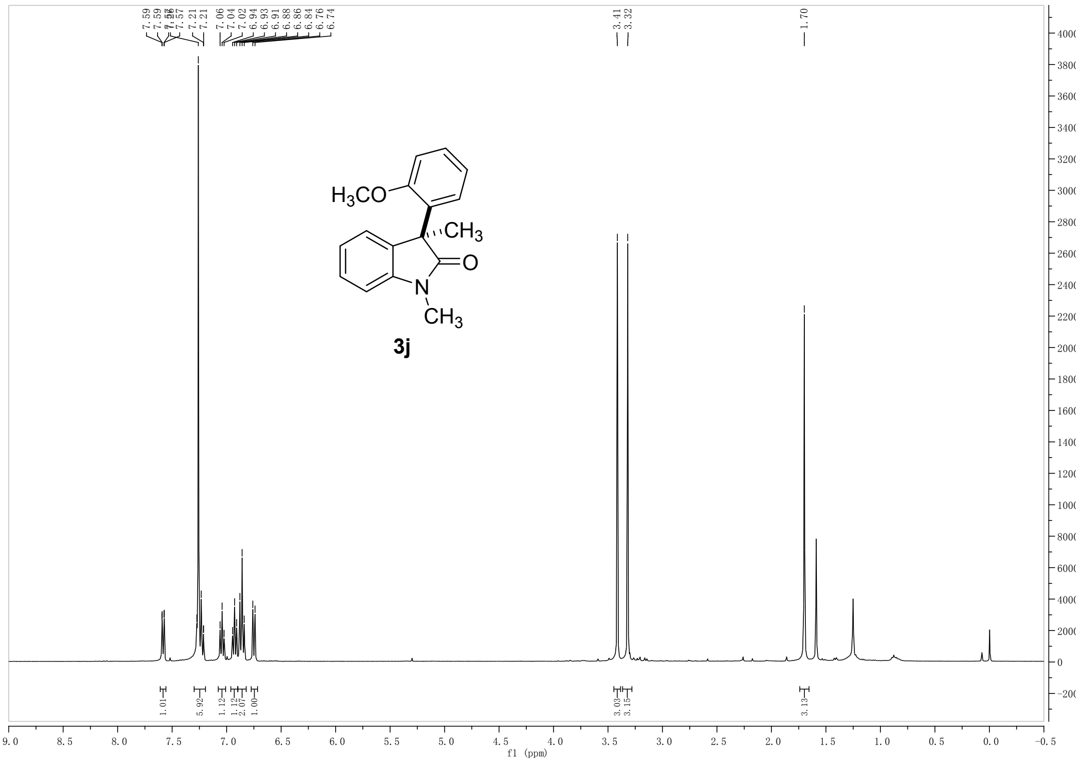

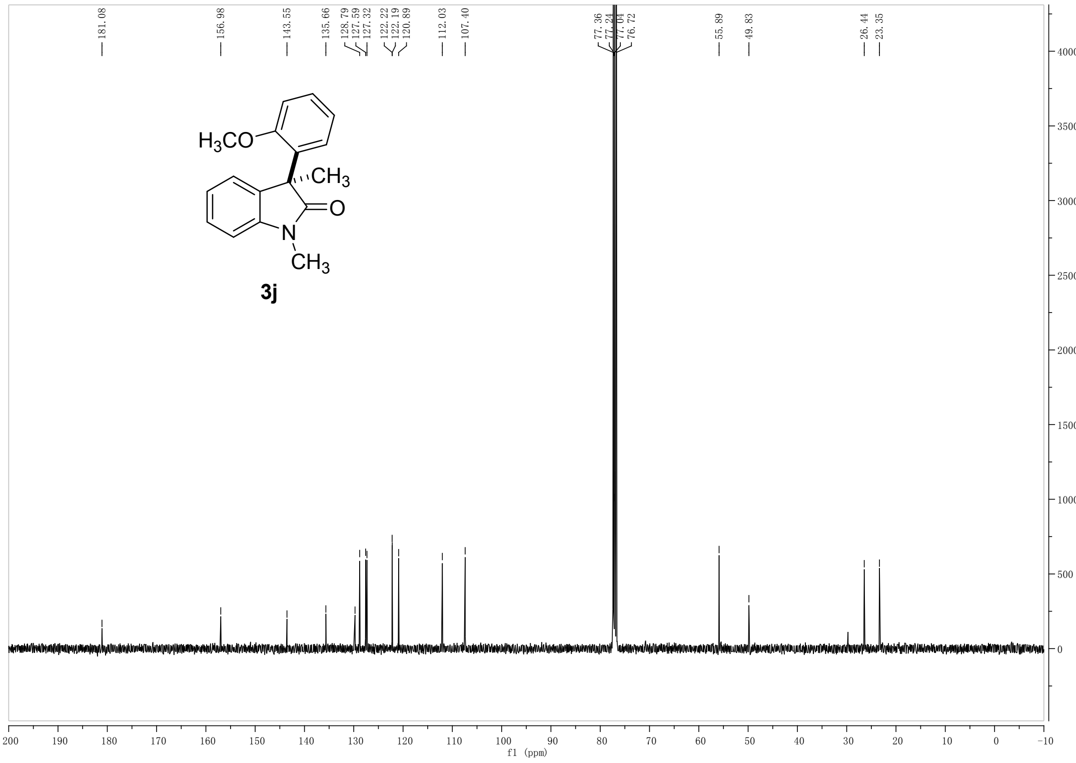

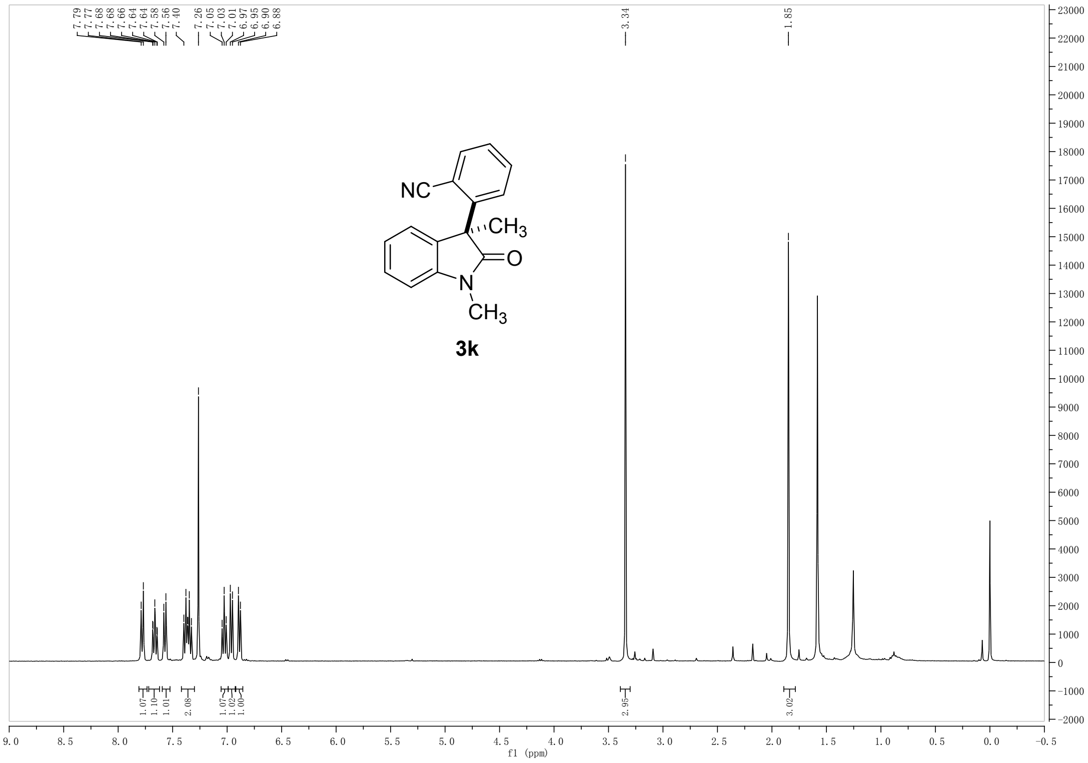
7.79
7.77
7.68
7.66
7.64
7.58
7.56
7.40
7.26
7.05
7.03
7.01
6.97
6.95
6.90
6.88
3.34
1.85
NC
CH3
O
N
CH3
3k
1.07
1.10
1.01
2.08
1.07
1.02
1.00
2.95
3.02
f1 (ppm)

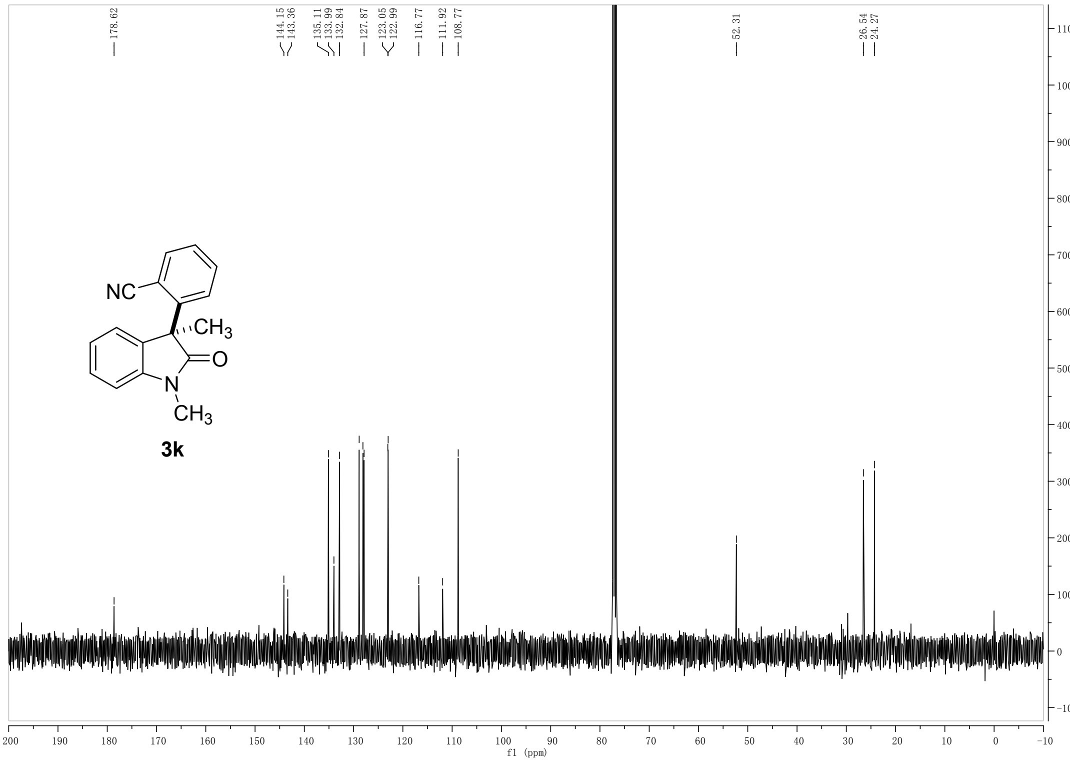
178.62
144.15
143.36
135.11
133.99
132.84
127.87
123.05
122.99
116.77
111.92
108.77
52.31
26.54
24.27
NC
CH3
O
N
CH3
3k
f1 (ppm)

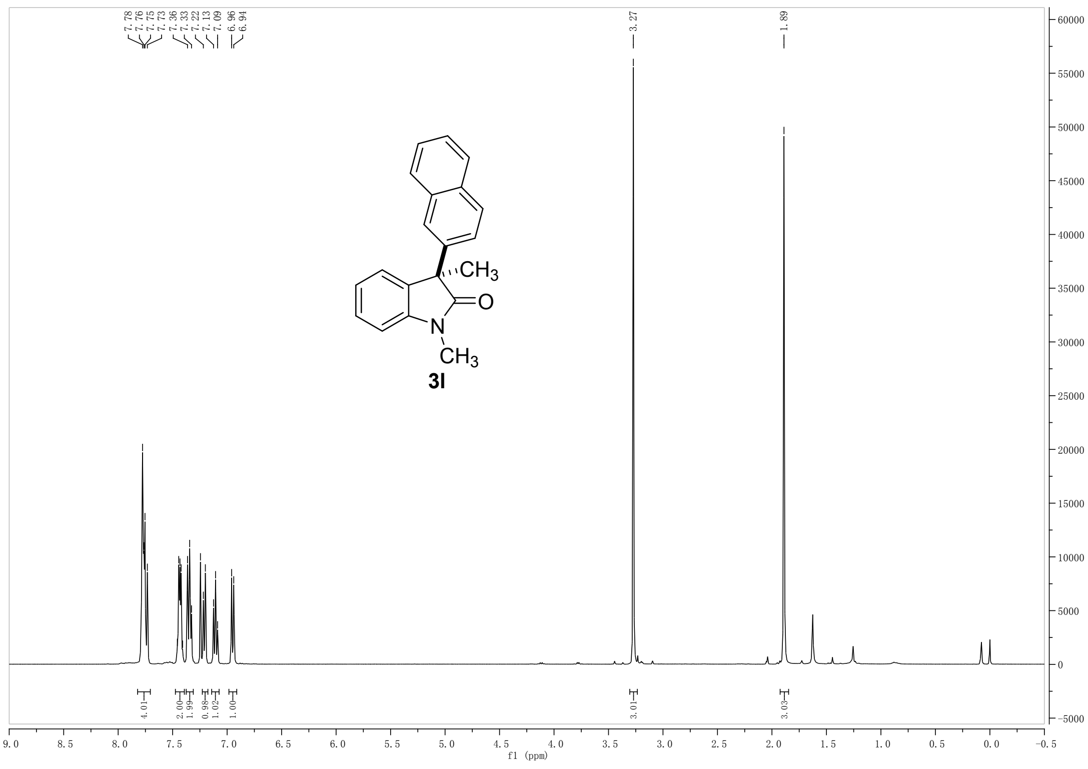
7.78
7.76
7.75
7.73
7.36
7.33
7.22
7.13
7.09
6.96
6.94
3.27
1.89
CH3
O
N
CH3
3l
4.01
2.00
1.99
0.98
1.02
1.00
3.01
3.03
f1 (ppm)

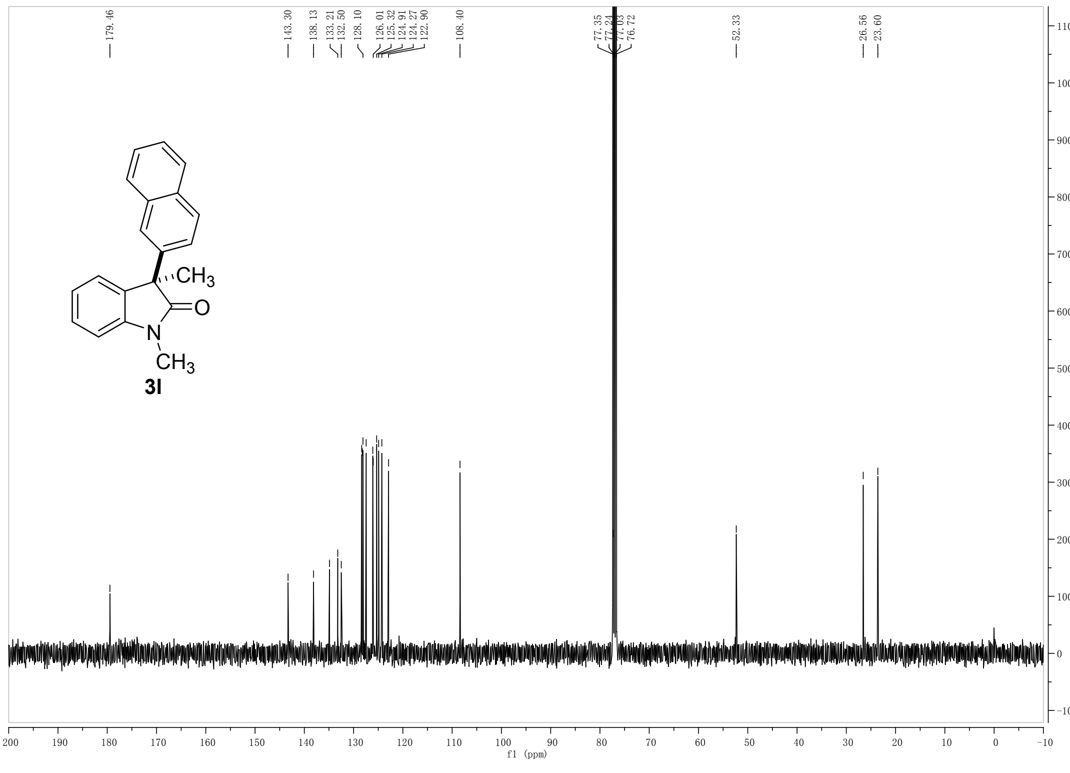
179.46
143.30
138.13
133.21
132.50
128.10
126.01
125.32
124.91
124.27
122.90
108.40
77.35
76.72
52.33
26.56
23.60
CH3
O
N
CH3
3l
f1 (ppm)

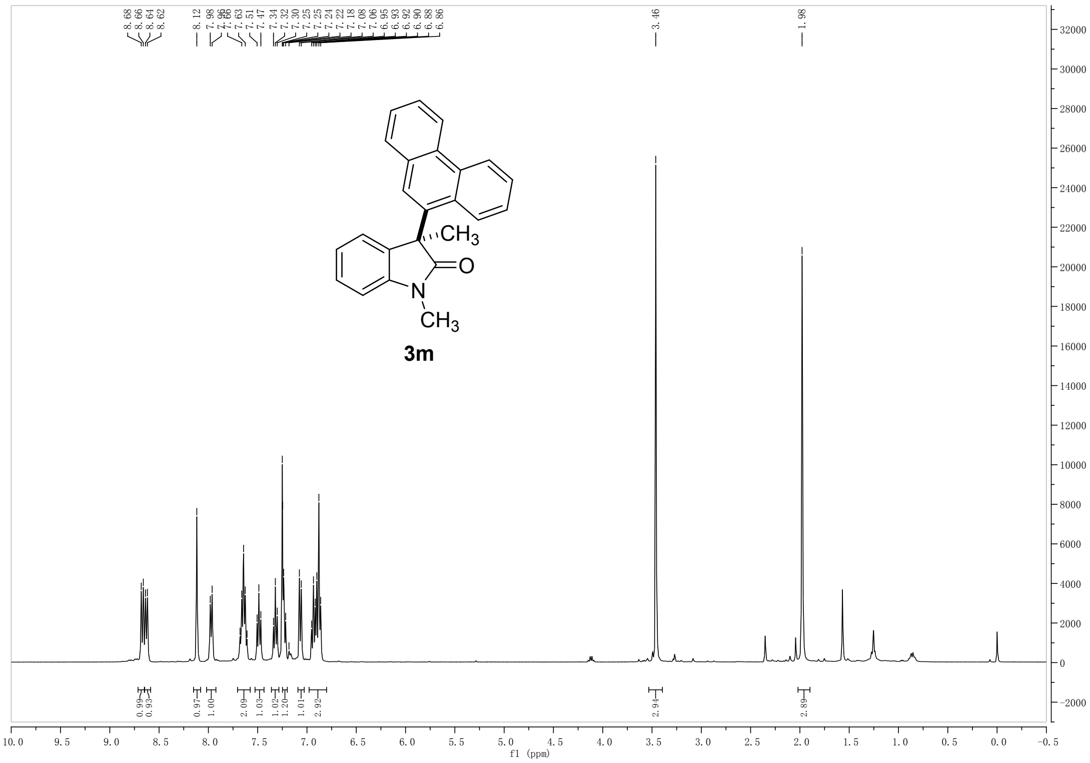
3m
f1 (ppm)

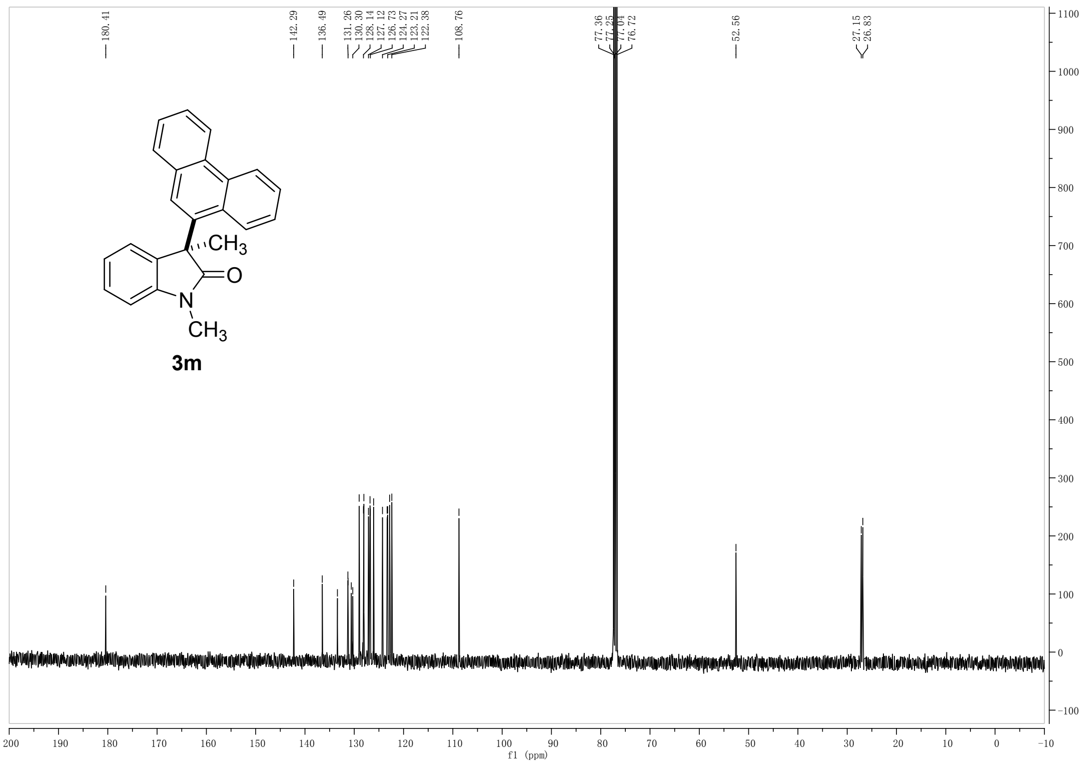
3m
f1 (ppm)

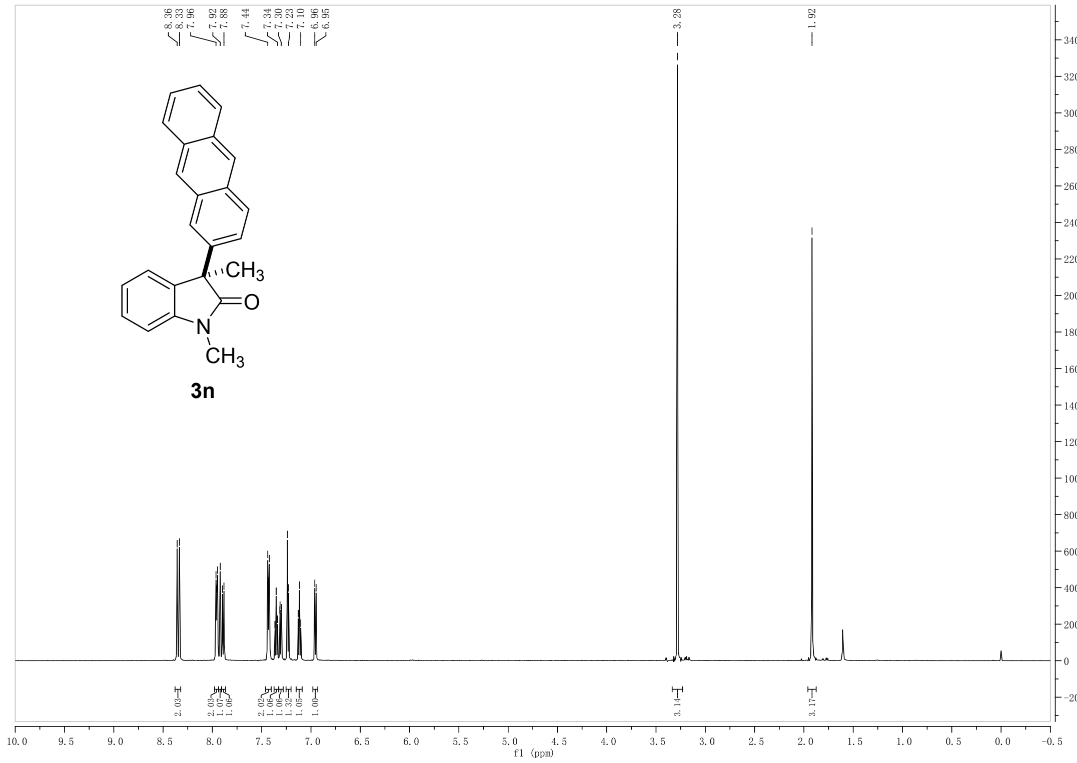

3n

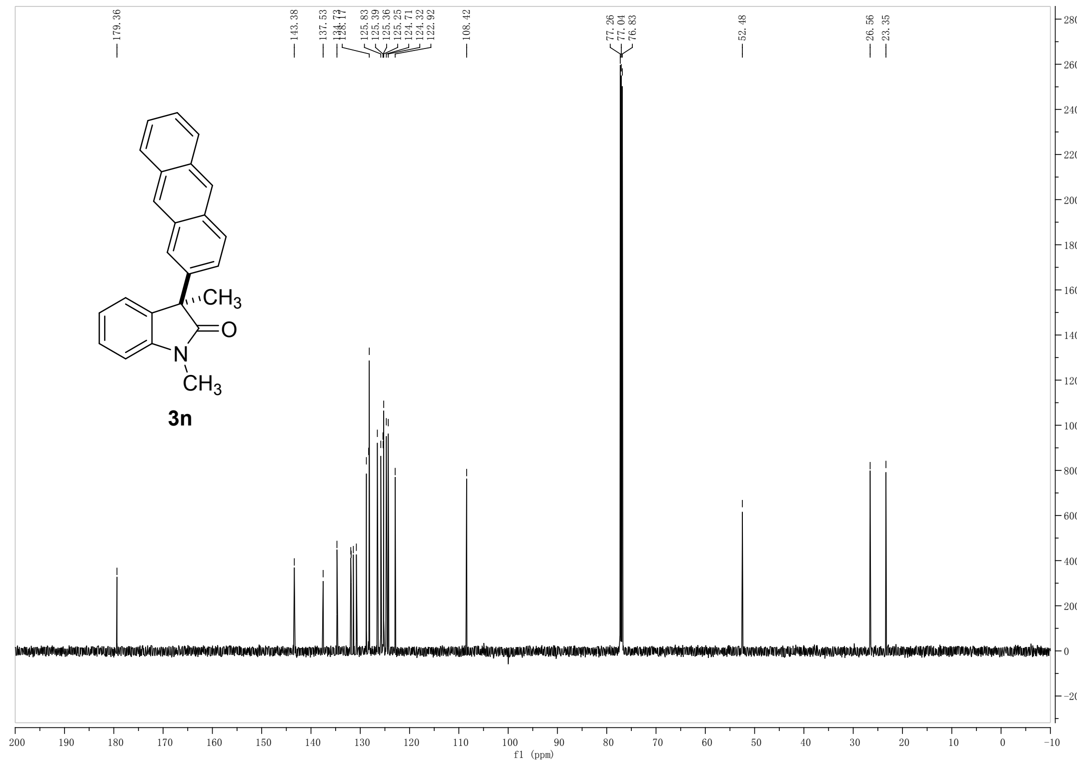

3n

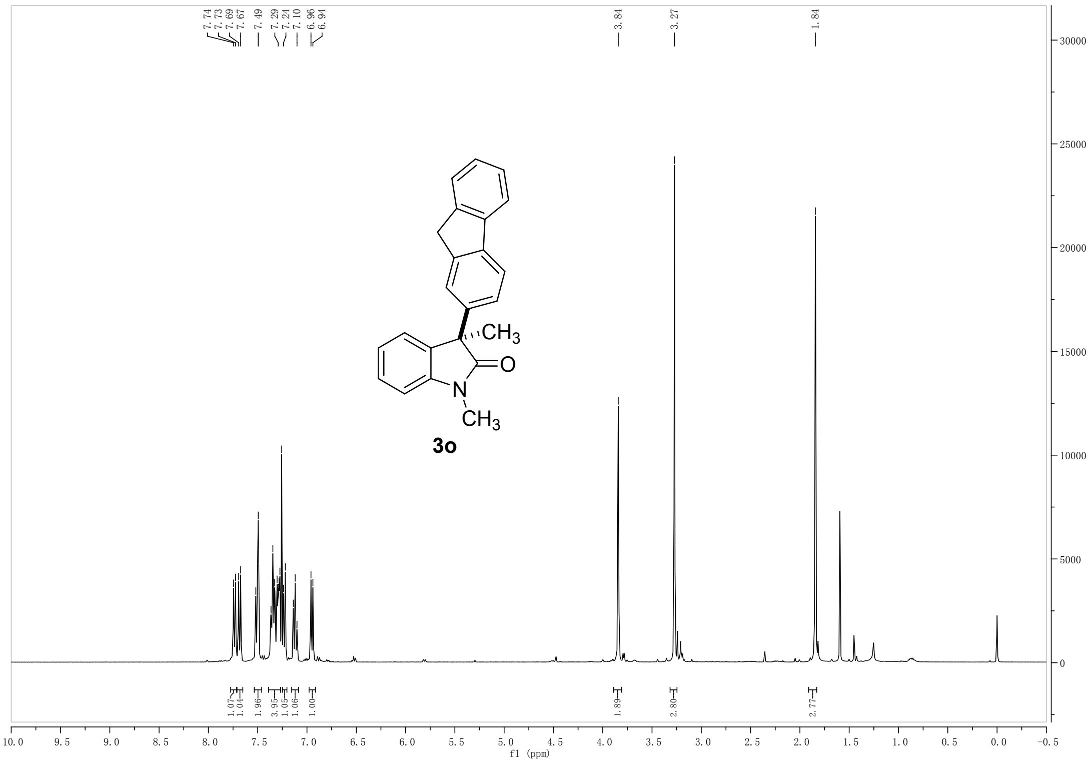

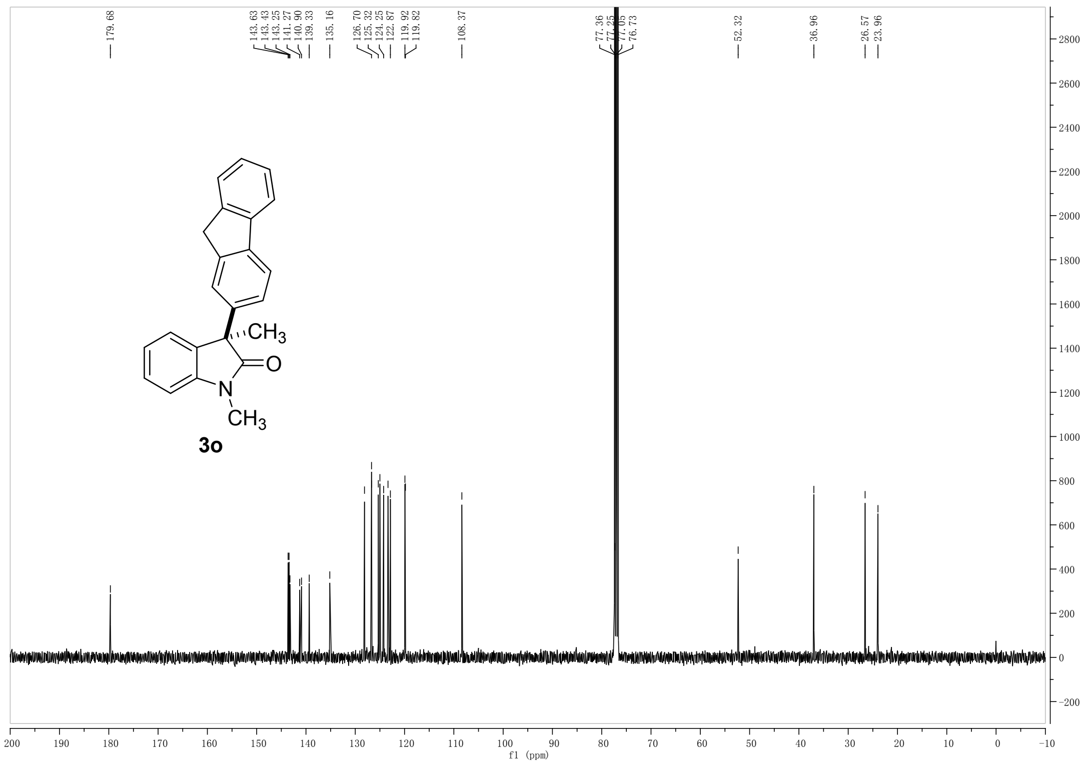

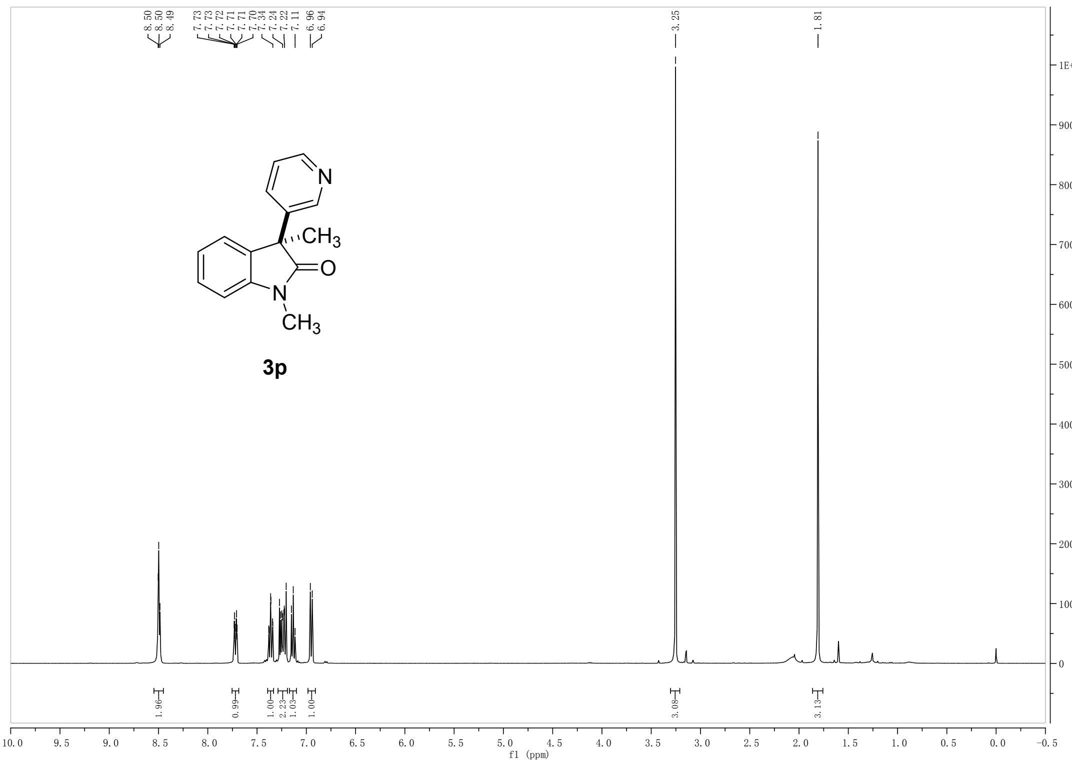

8.50
8.50
8.49
7.73
7.73
7.72
7.71
7.71
7.70
7.34
7.24
7.22
7.11
6.96
6.94
3.25
1.81
N
CH3
O
N
CH3
3p
1.96
0.99
1.00
2.23
1.03
1.00
3.08
3.13
f1 (ppm)

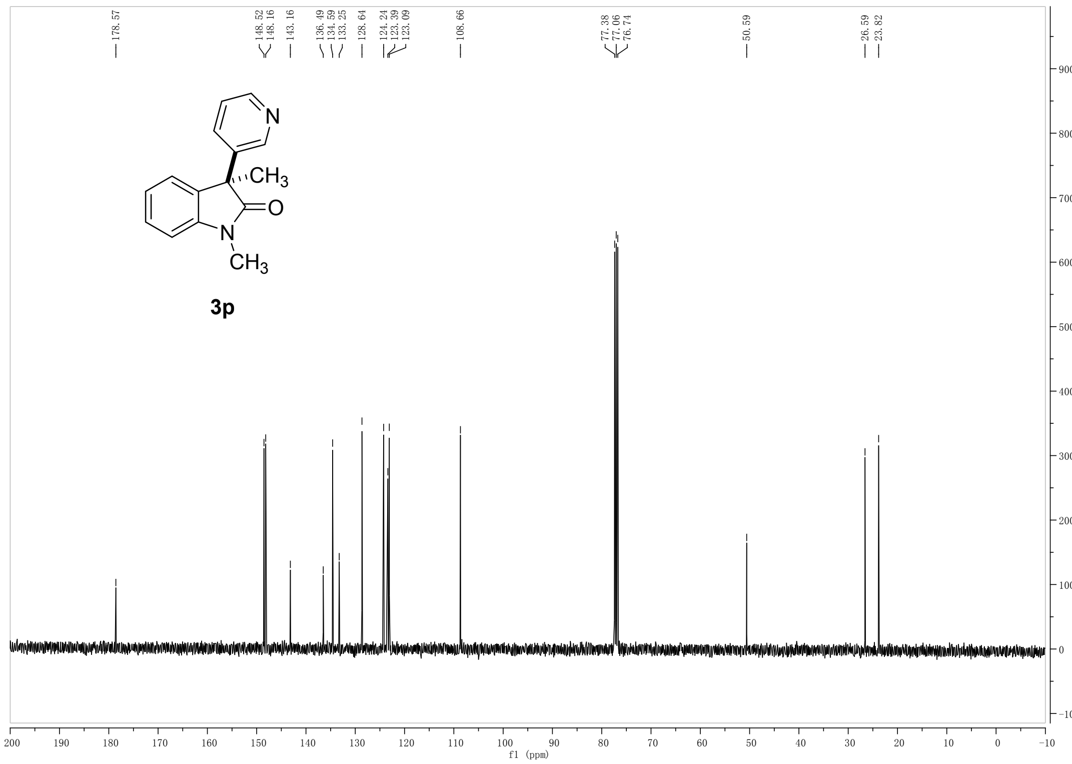

178.57
148.52
148.16
143.16
136.49
134.59
133.25
128.64
124.24
123.39
123.09
108.66
77.38
77.06
76.74
50.59
26.59
23.82
N
CH3
O
N
CH3
3p
f1 (ppm)

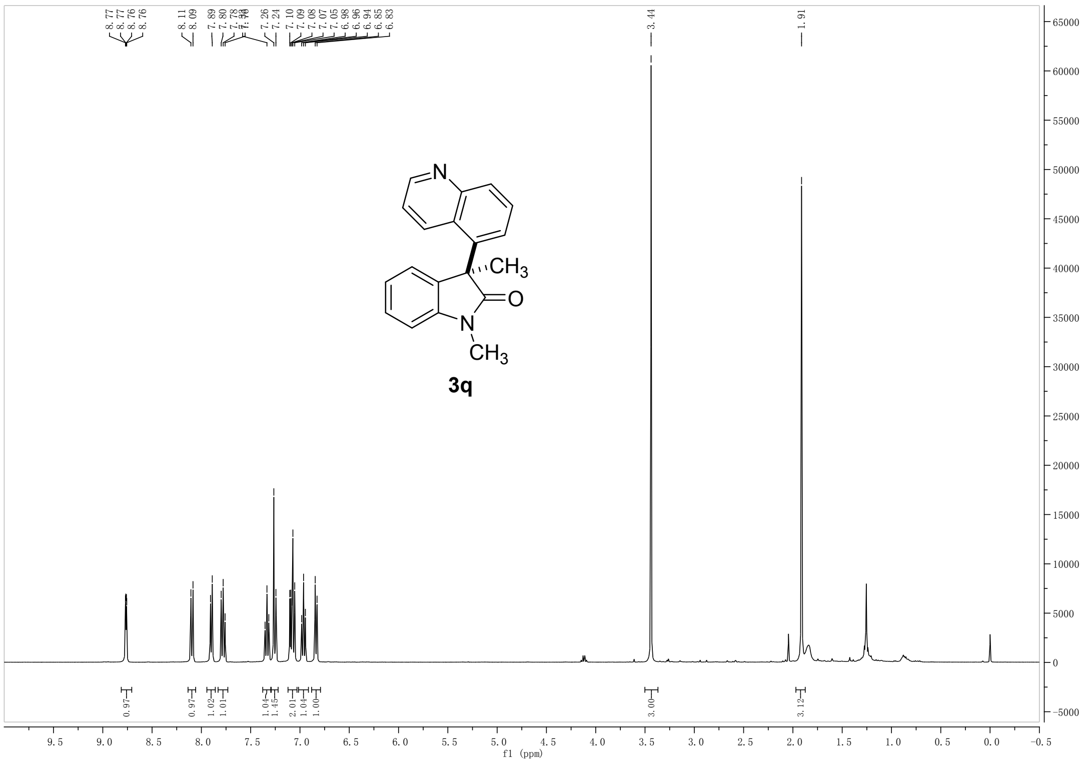
3q

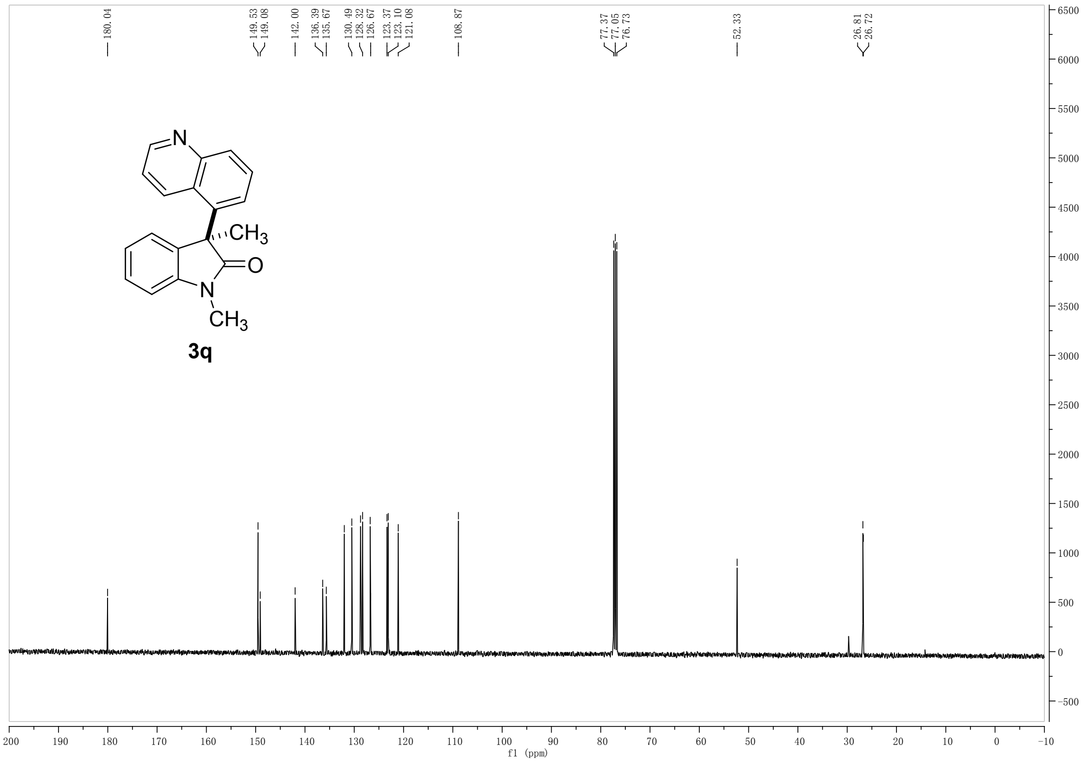
3q

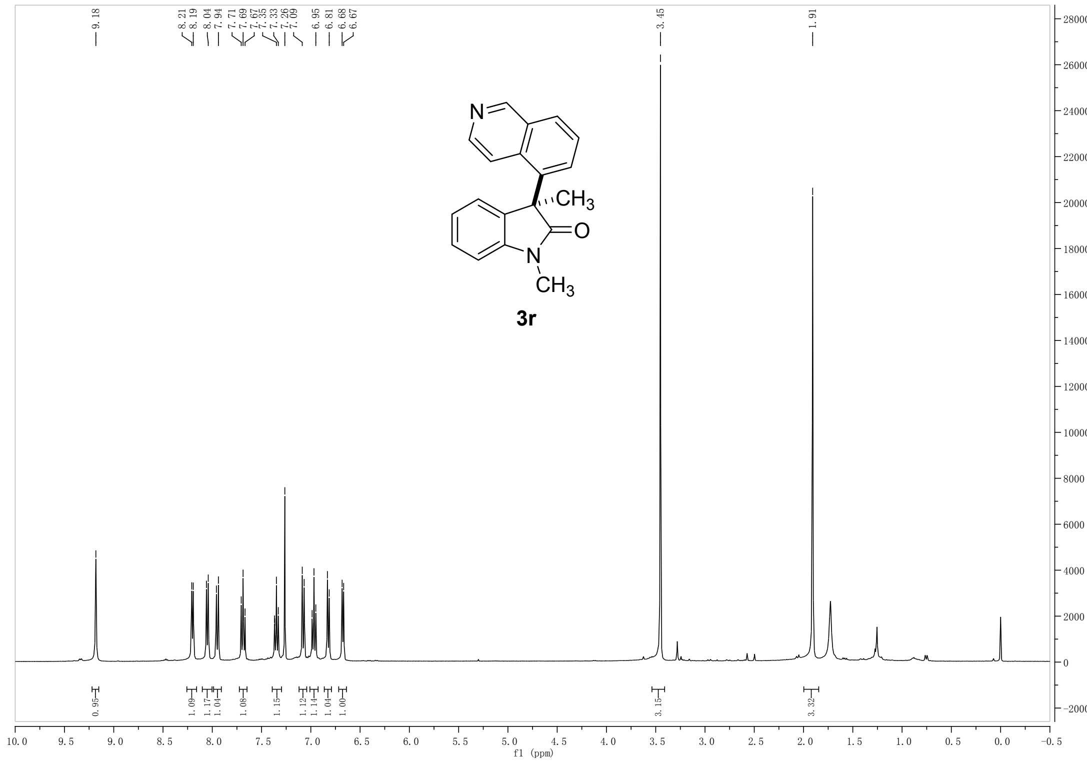

9.18
8.21
8.19
8.04
7.94
7.71
7.69
7.67
7.35
7.33
7.26
7.09
6.95
6.81
6.68
6.67
3.45
1.91
0.95
1.09
1.17
1.04
1.08
1.15
1.12
1.14
1.04
1.00
3.15
3.32
N
CH3
O
N
CH3
3r
f1 (ppm)

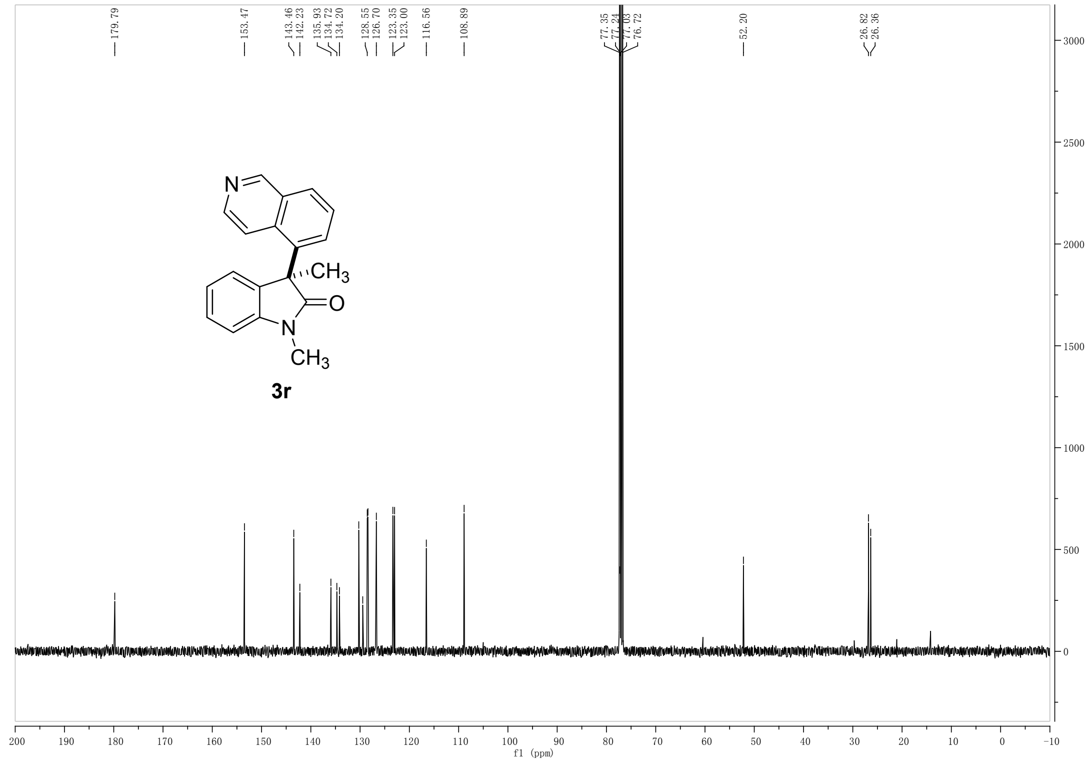

179.79
153.47
143.46
142.23
135.93
134.72
134.20
128.55
126.70
123.35
123.00
116.56
108.89
77.35
76.72
52.20
26.82
26.36
N
CH3
O
N
CH3
3r
f1 (ppm)

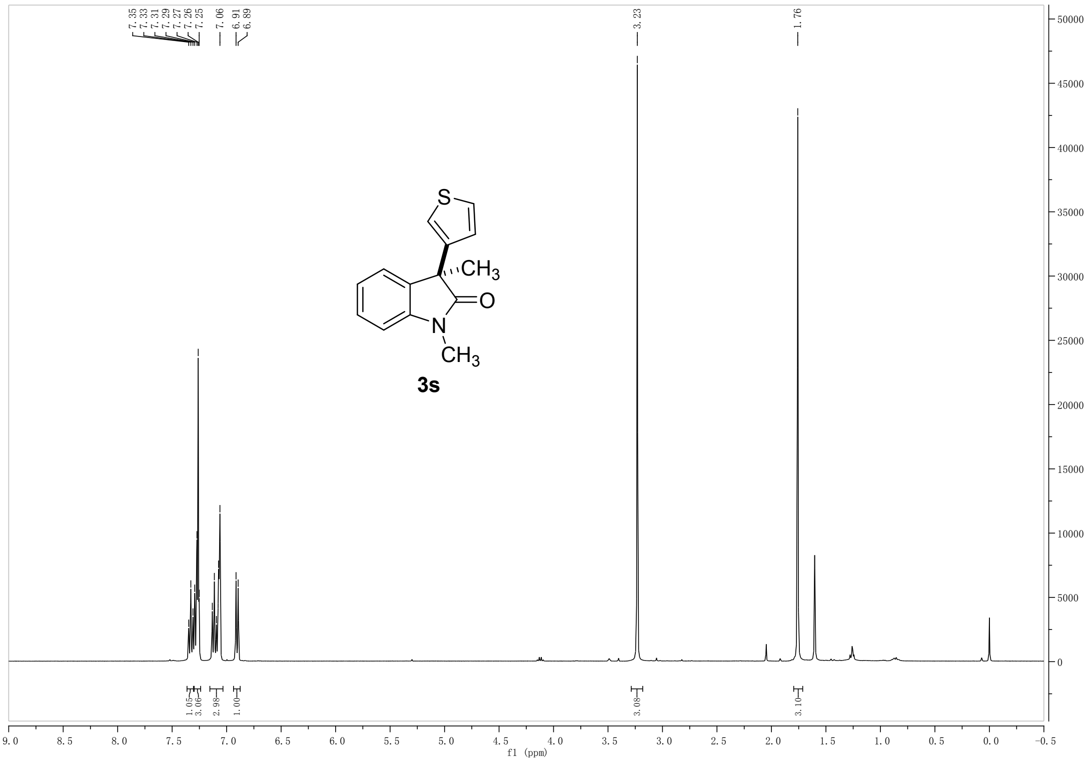

7.35
7.33
7.31
7.29
7.27
7.26
7.25
7.06
6.91
6.89
3.23
1.76
3s
f1 (ppm)

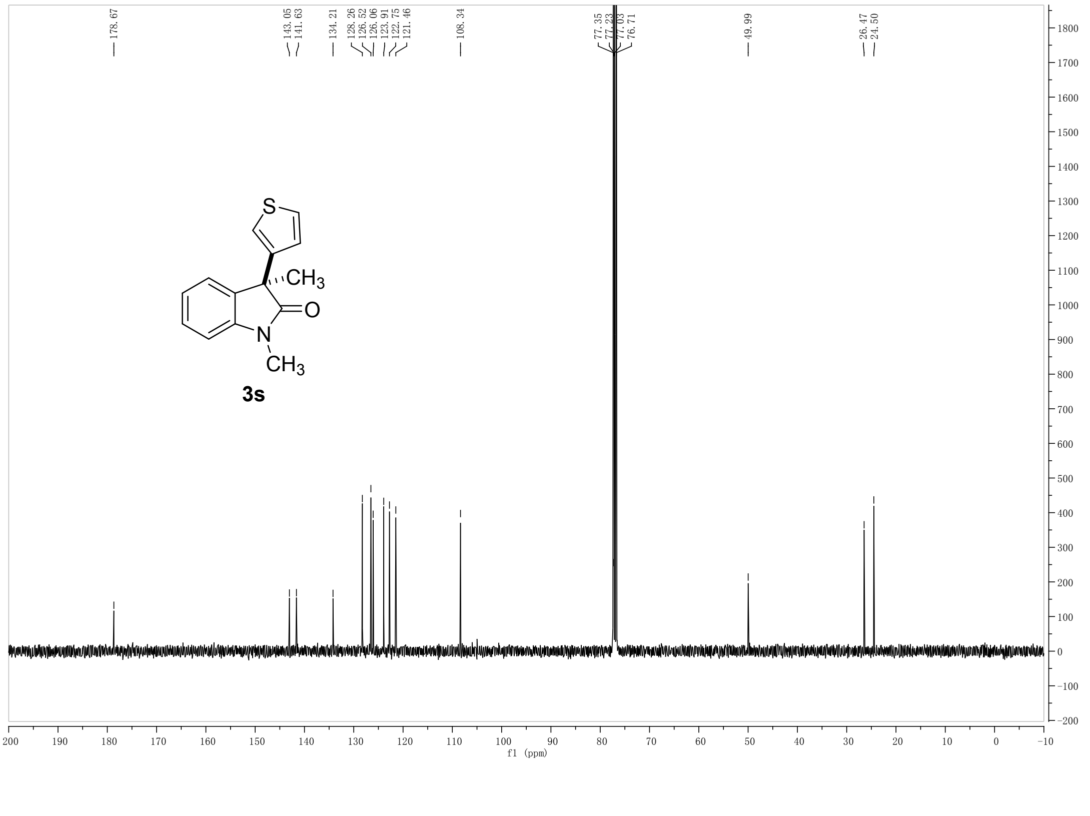

178.67
143.05
141.63
134.21
128.26
126.52
126.06
123.91
122.75
121.46
108.34
77.35
76.71
49.99
26.47
24.50
3s
f1 (ppm)

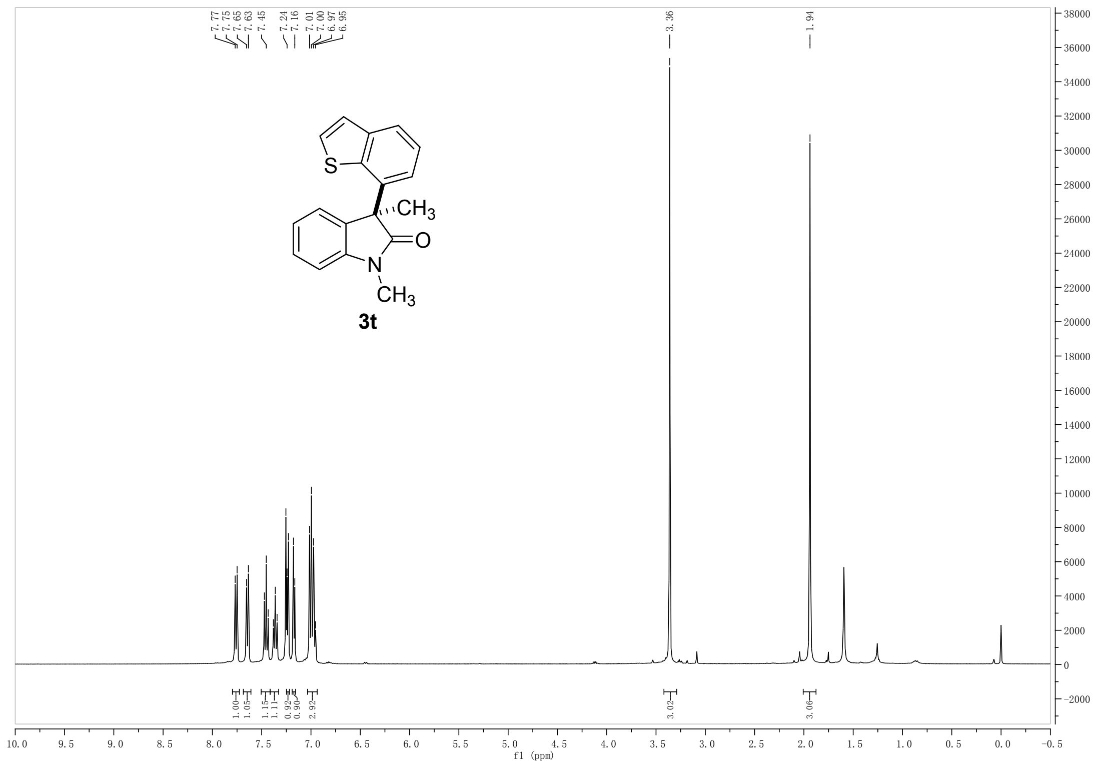
7.77
7.75
7.65
7.63
7.45
7.24
7.16
7.01
7.00
6.97
6.95
3.36
1.94
3t
f1 (ppm)

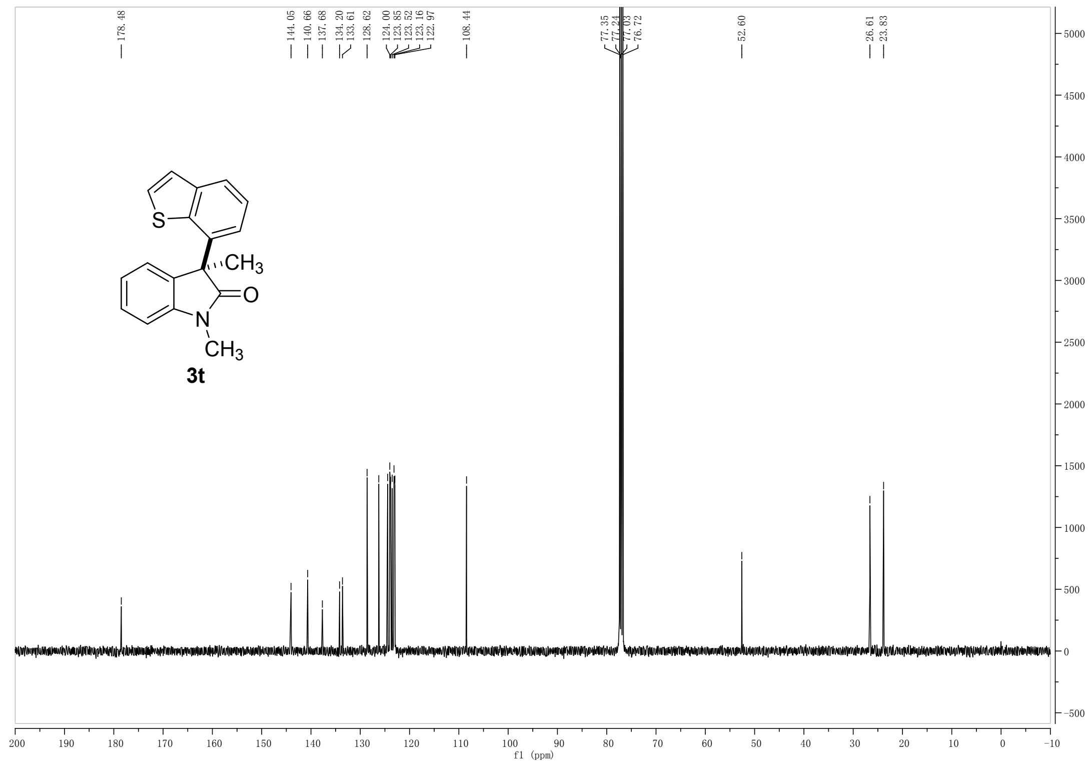
178.48
144.05
140.66
137.68
134.20
133.61
128.62
124.00
123.85
123.52
123.16
122.97
108.44
77.35
76.72
52.60
26.61
23.83
3t
f1 (ppm)

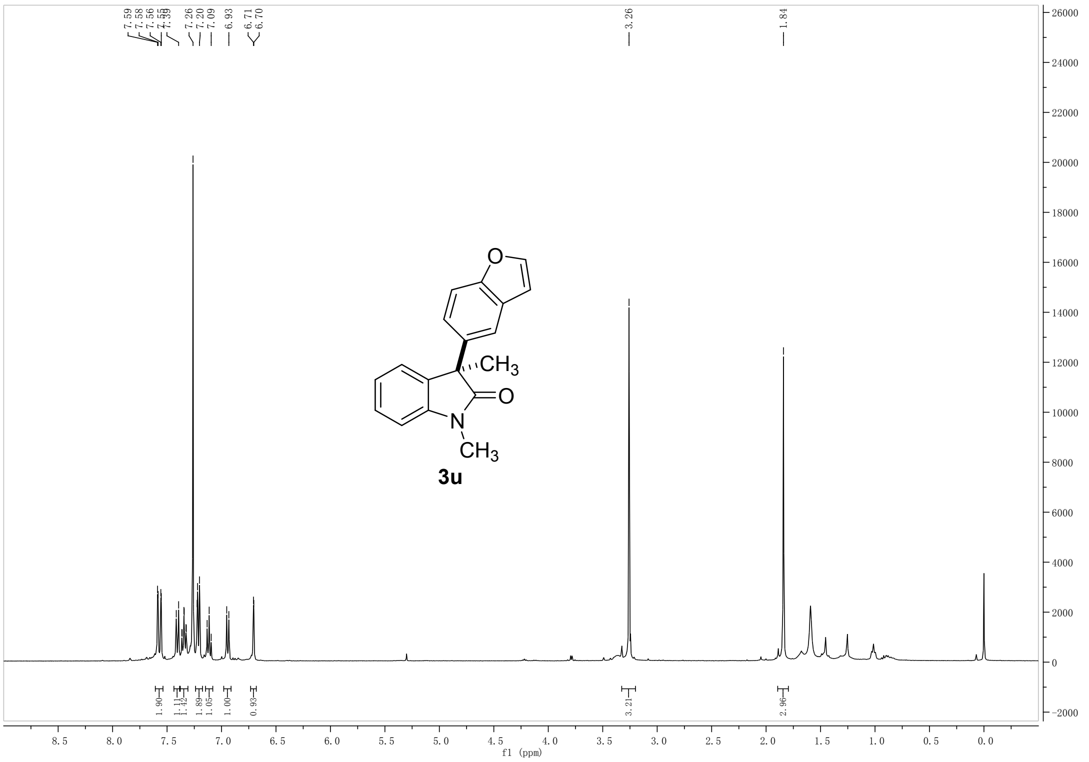
7.59
7.58
7.56
7.55
7.39
7.26
7.20
7.09
6.93
6.71
6.70
3.26
1.84
1.90
1.11
1.42
1.89
1.05
1.00
0.93
3.21
2.96
O
CH3
O
N
CH3
3u
f1 (ppm)

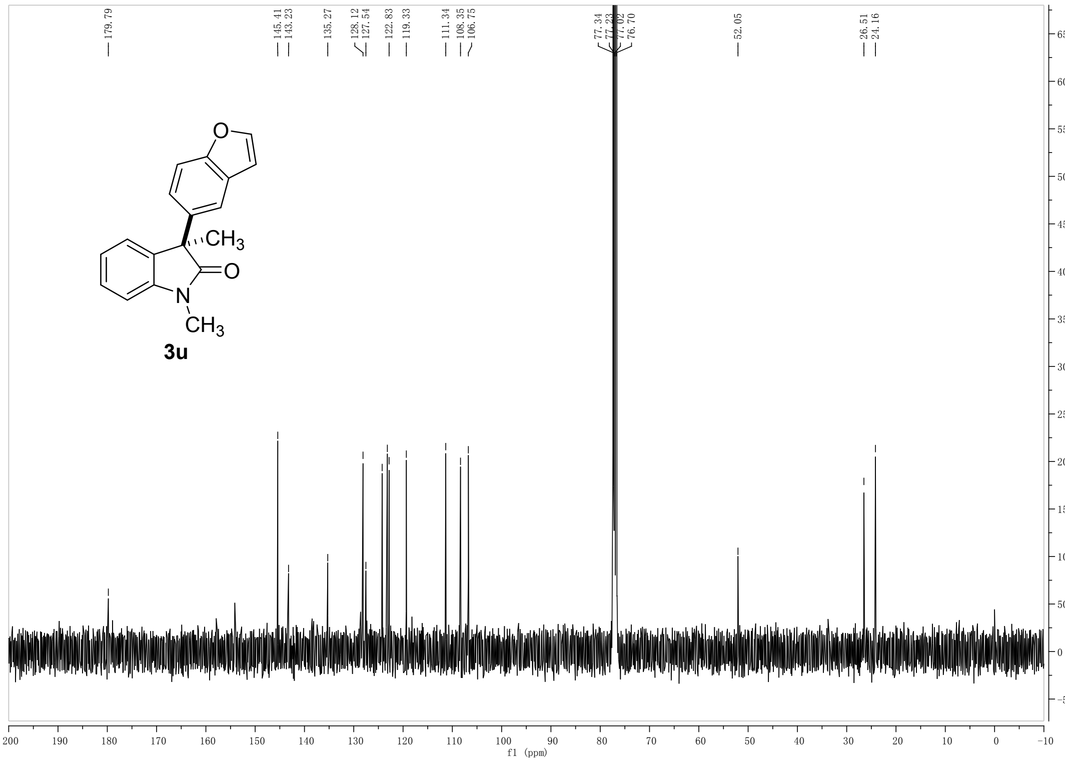
179.79
145.41
143.23
135.27
128.12
127.54
122.83
119.33
111.34
108.35
106.75
77.34
76.70
52.05
26.51
24.16
O
CH3
O
N
CH3
3u
f1 (ppm)

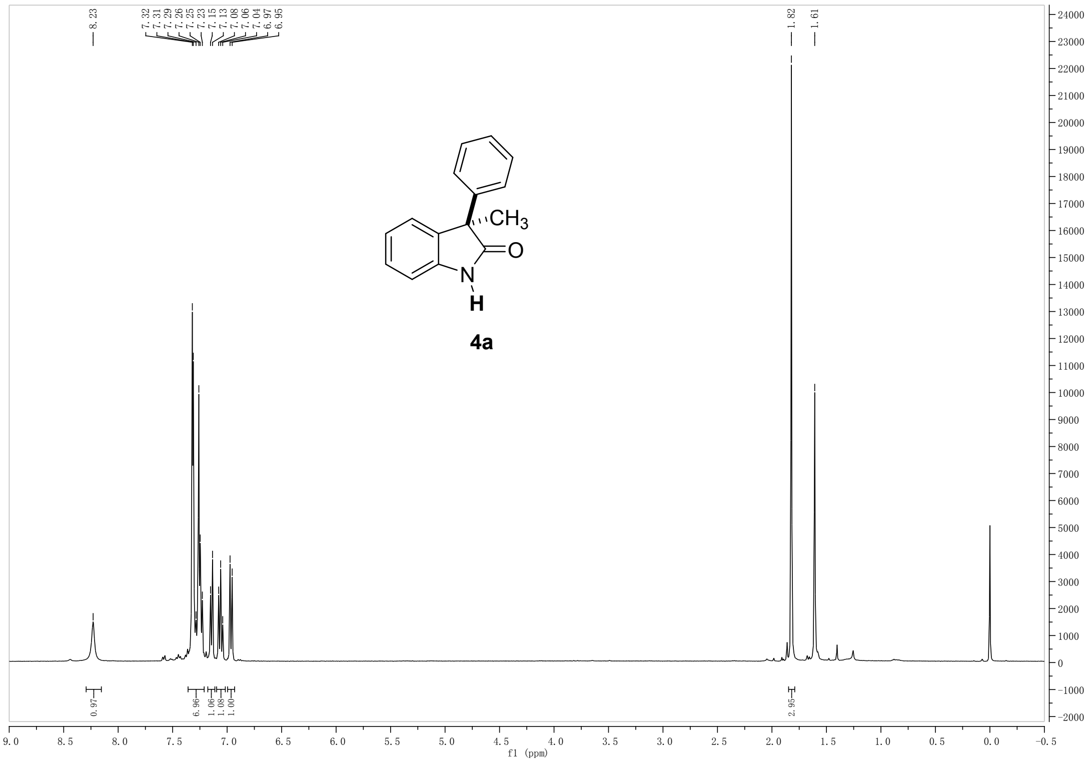
8.23
7.32
7.31
7.29
7.26
7.25
7.23
7.15
7.13
7.08
7.06
7.04
6.97
6.95
1.82
1.61
4a
0.97
6.96
1.06
1.08
1.00
2.95
f1 (ppm)

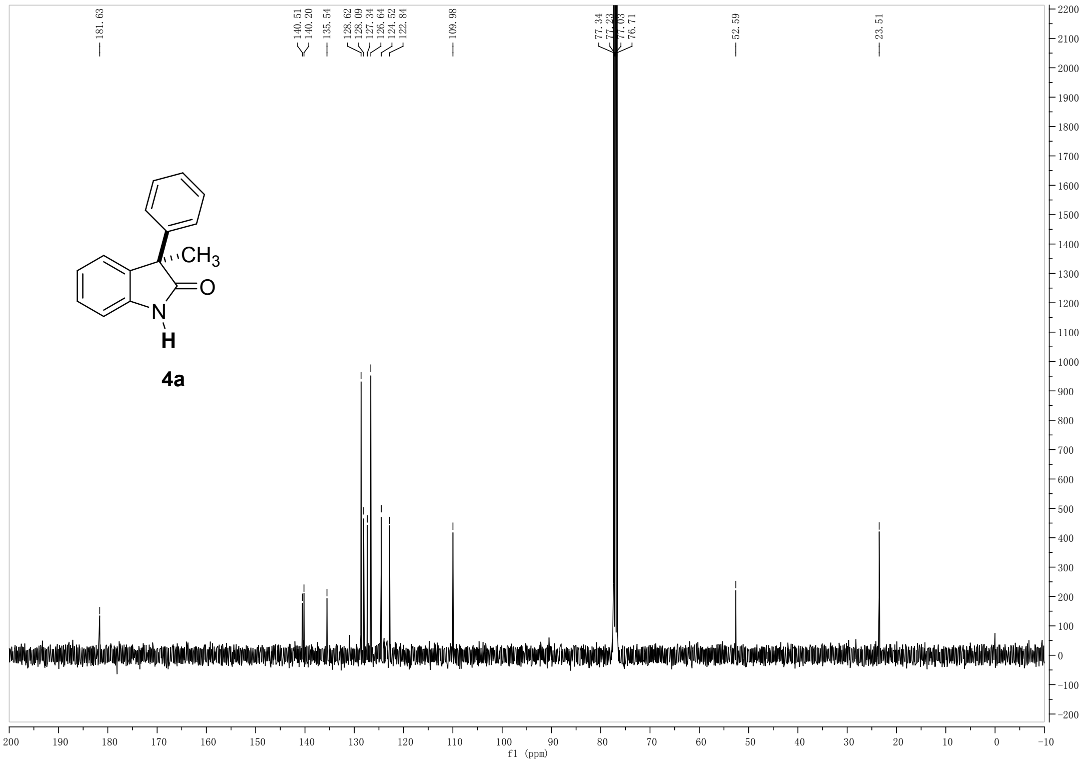
181.63
140.51
140.20
135.54
128.62
128.09
127.34
126.64
124.52
122.84
109.98
77.34
76.71
52.59
23.51
4a
f1 (ppm)

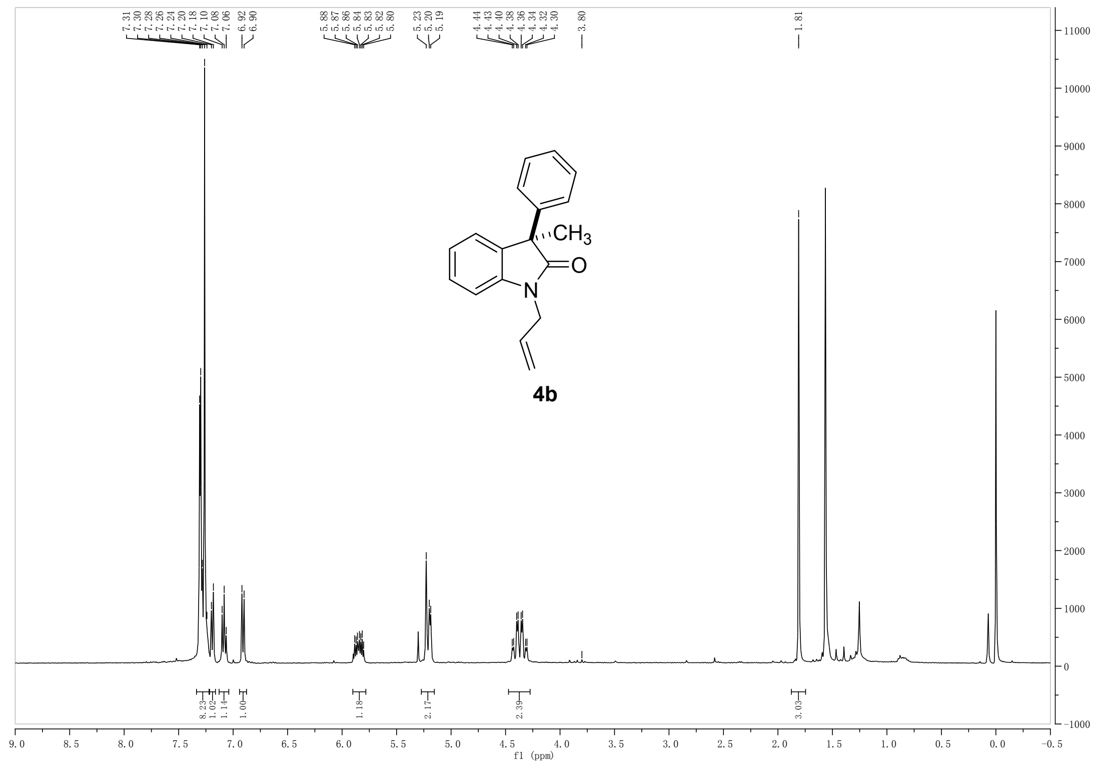

7.31
7.30
7.28
7.26
7.24
7.20
7.18
7.10
7.08
7.06
6.92
6.90
5.88
5.87
5.86
5.84
5.83
5.82
5.80
5.23
5.20
5.19
4.44
4.43
4.40
4.38
4.36
4.34
4.32
4.30
3.80
1.81
CH3
O
N
4b
8.23
1.02
1.14
1.00
1.18
2.17
2.39
3.03
f1 (ppm)

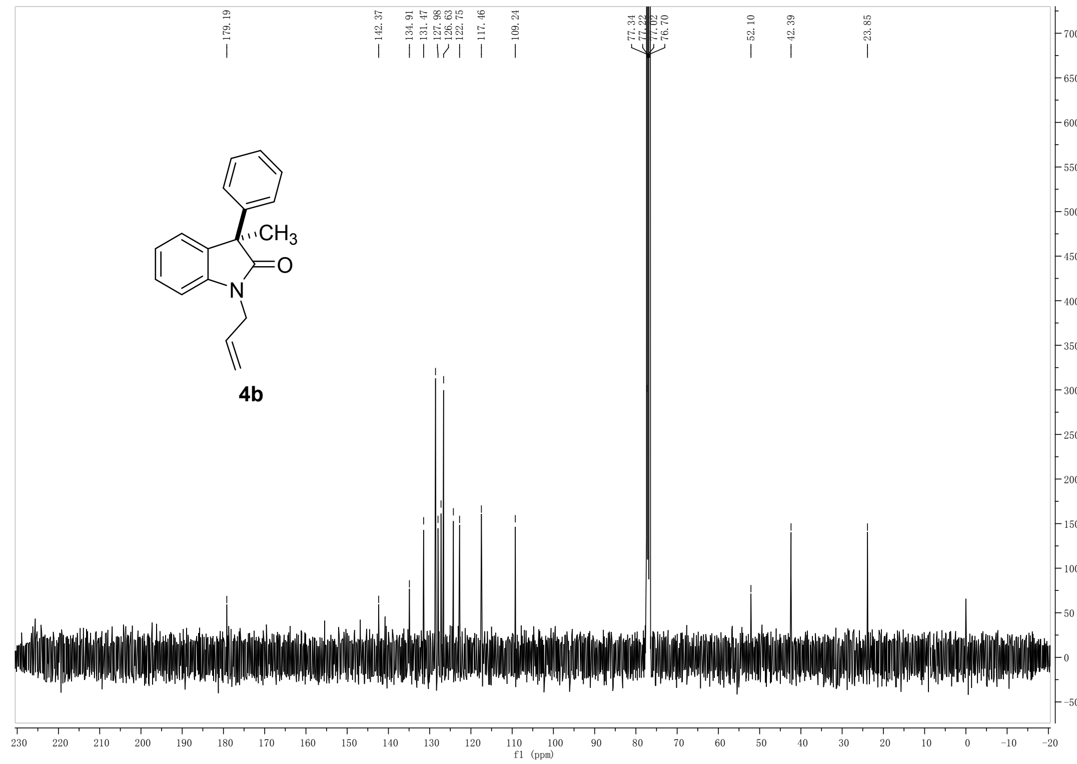

179.19
142.37
134.91
131.47
127.98
126.63
122.75
117.46
109.24
77.34
77.02
76.70
52.10
42.39
23.85
CH3
O
N
4b
f1 (ppm)

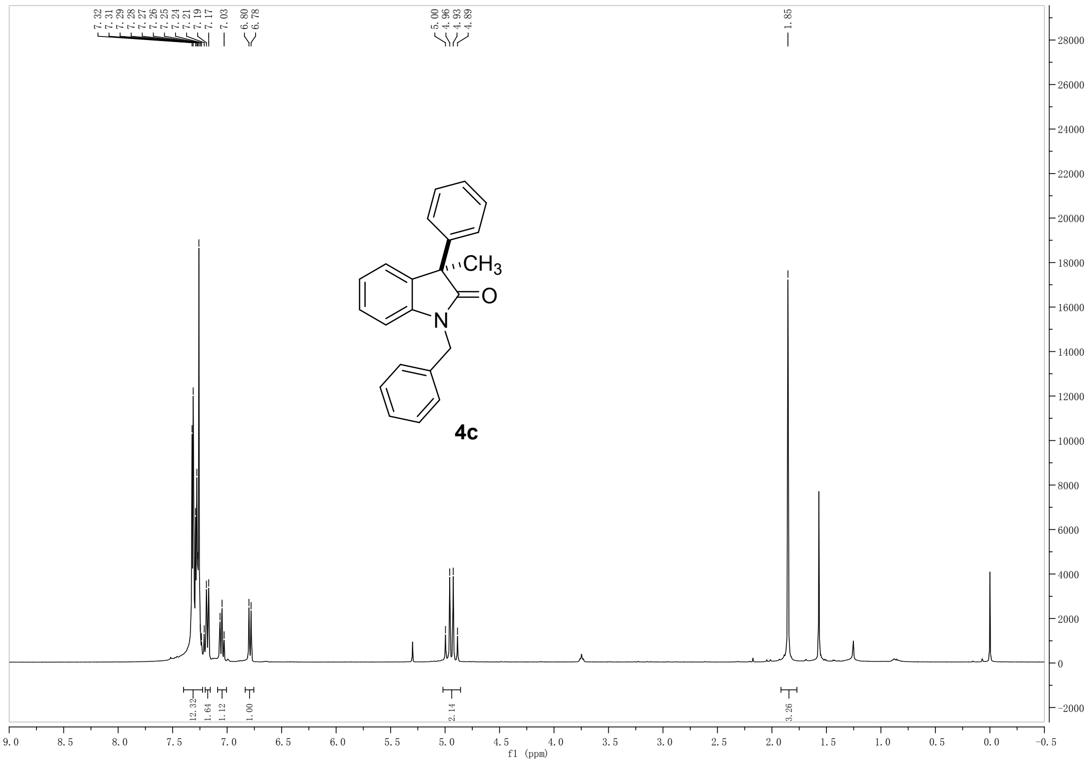
7.32
7.31
7.29
7.28
7.27
7.26
7.25
7.24
7.21
7.19
7.17
7.03
6.80
6.78
5.00
4.96
4.93
4.89
1.85
CH3
O
N
4c
12.32
1.64
1.12
1.00
2.14
3.26
f1 (ppm)

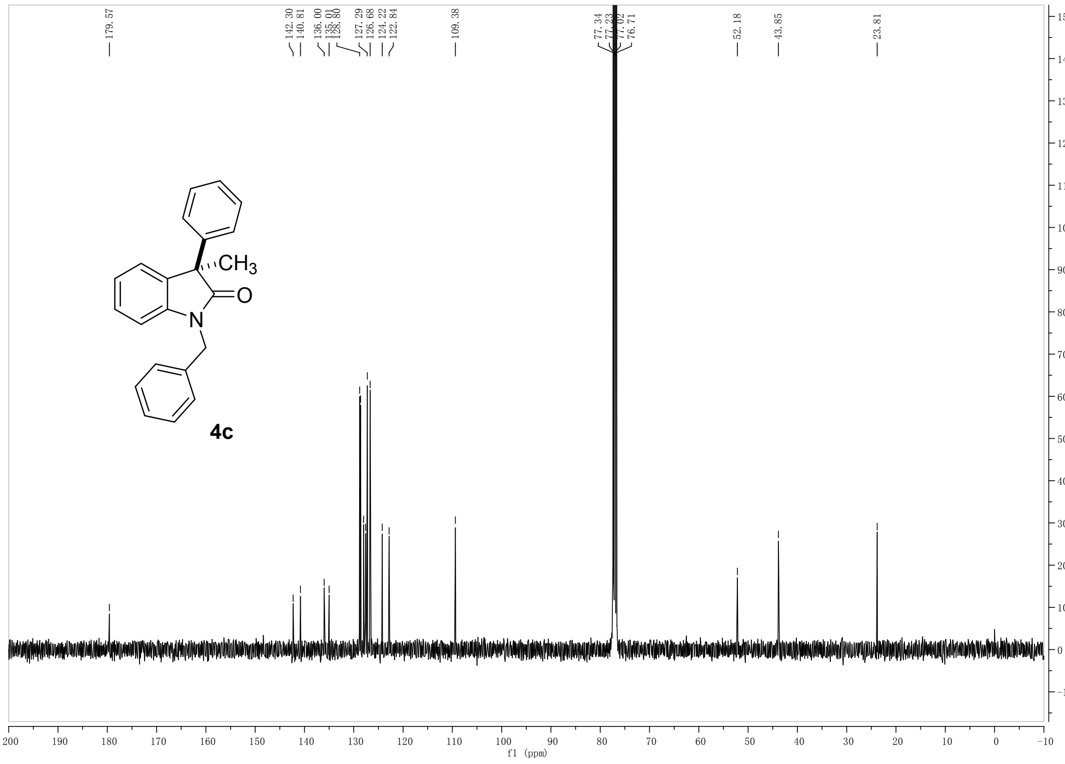
179.57
142.30
140.81
136.00
135.01
128.80
127.29
126.68
124.22
122.84
109.38
77.34
76.71
52.18
43.85
23.81
CH3
O
N
4c
f1 (ppm)

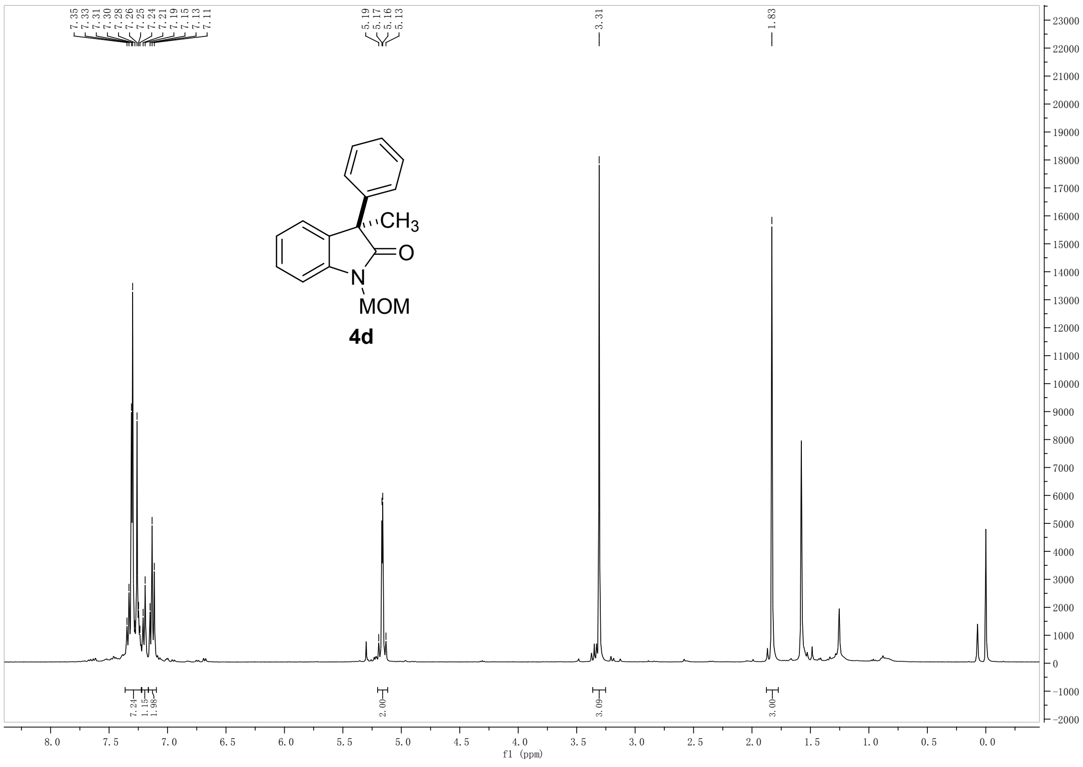

MOM
4d
f1 (ppm)

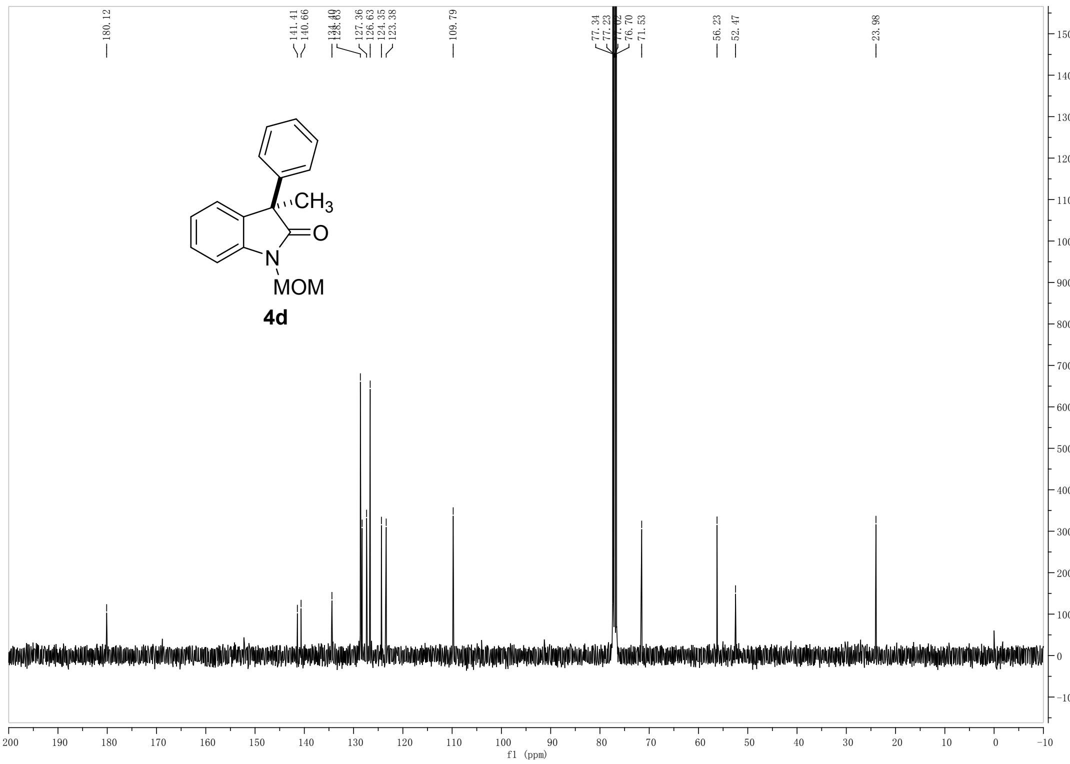

MOM
4d
f1 (ppm)

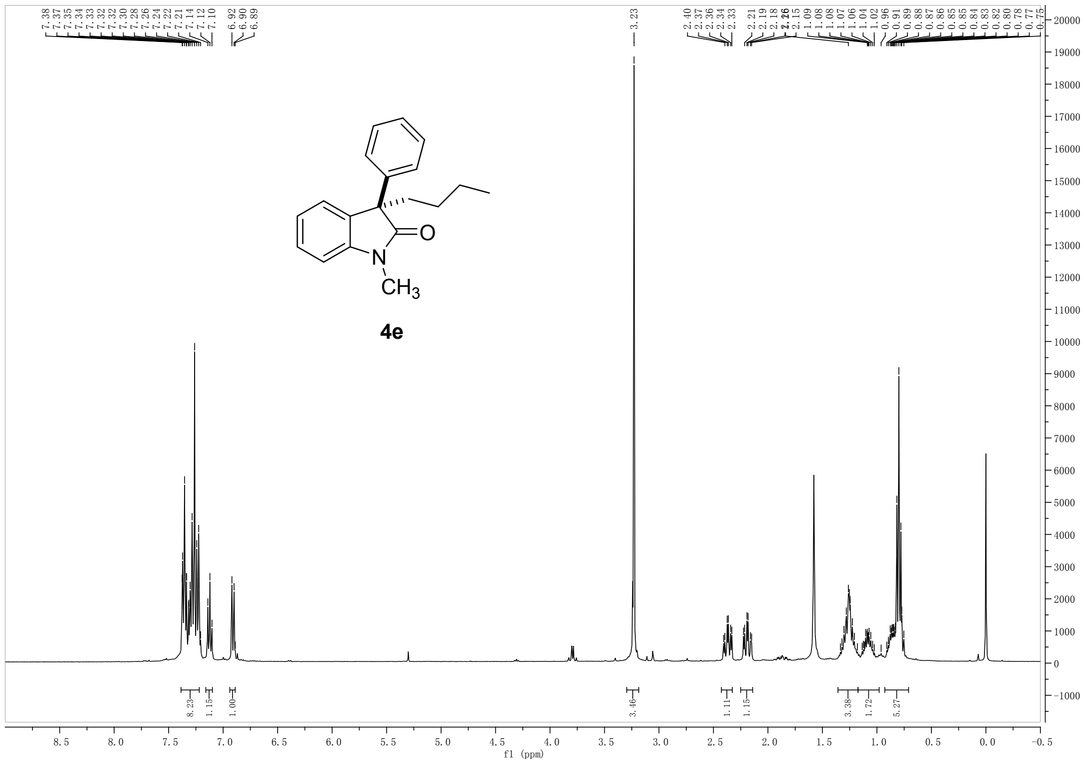
O
N
CH3
4e
f1 (ppm)

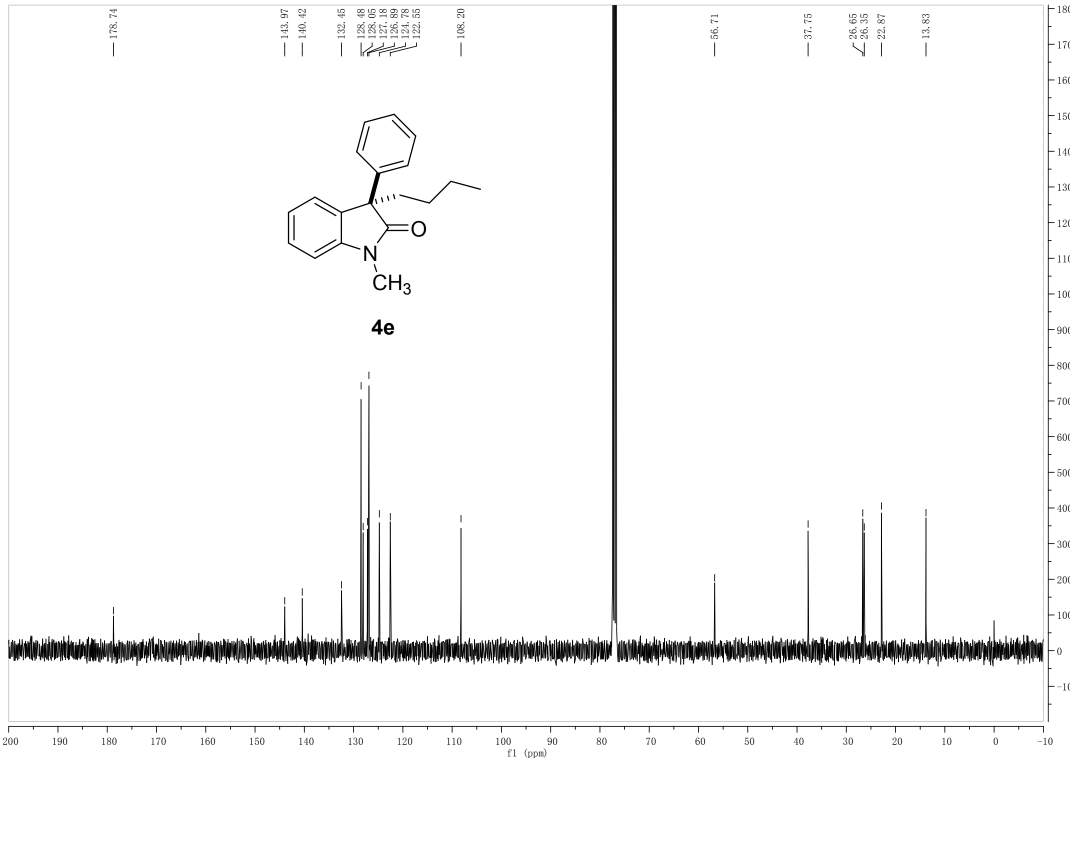
178.74
143.97
140.42
132.45
128.48
128.05
127.18
126.89
124.78
122.55
108.20
56.71
37.75
26.65
26.35
22.87
13.83
O
N
CH3
4e
f1 (ppm)

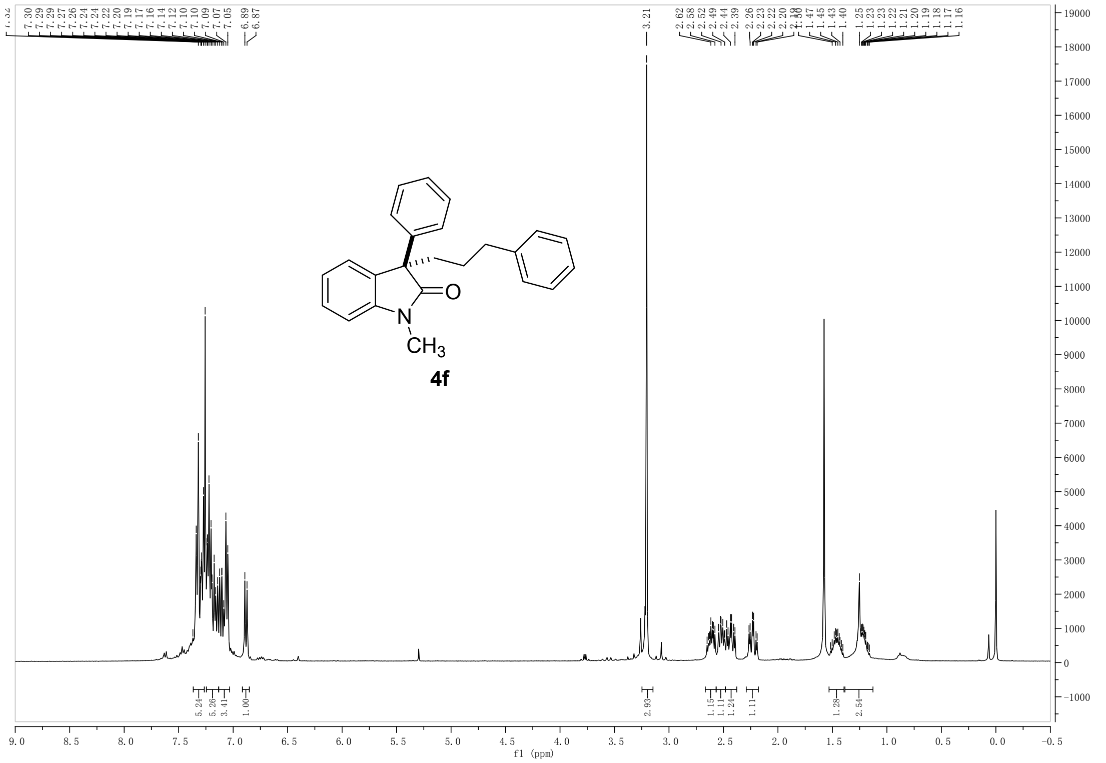

O
N
CH3
4f
f1 (ppm)

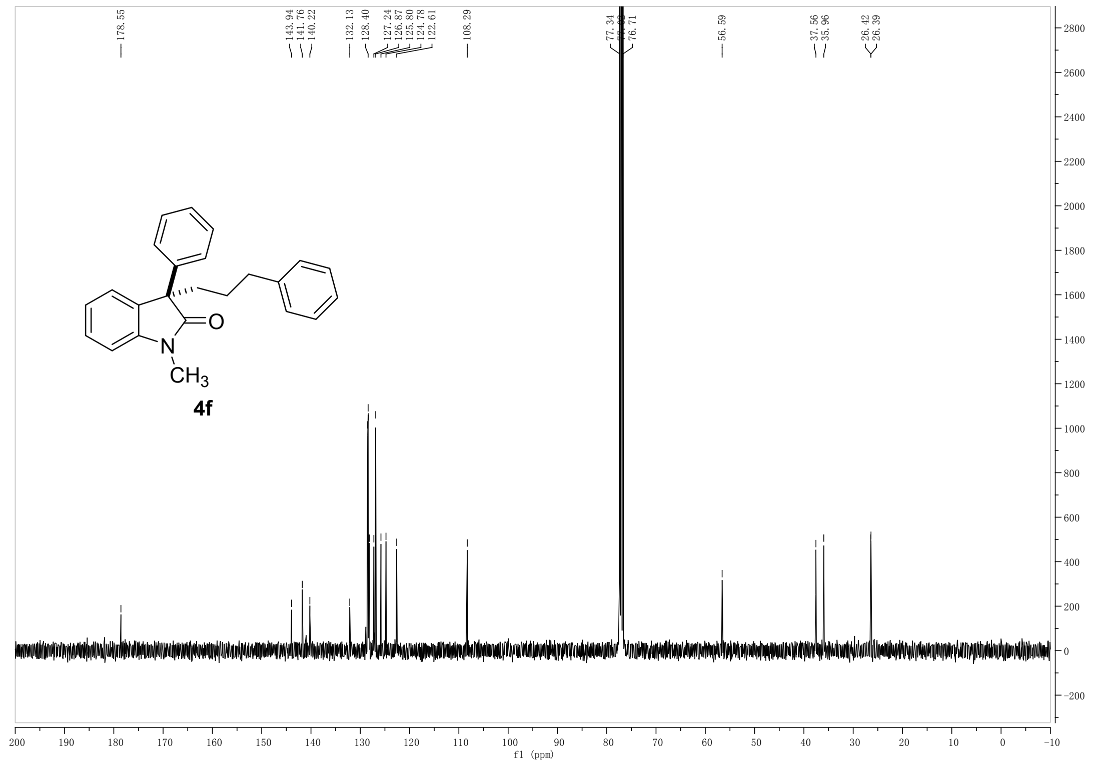

O
N
CH3
4f
f1 (ppm)

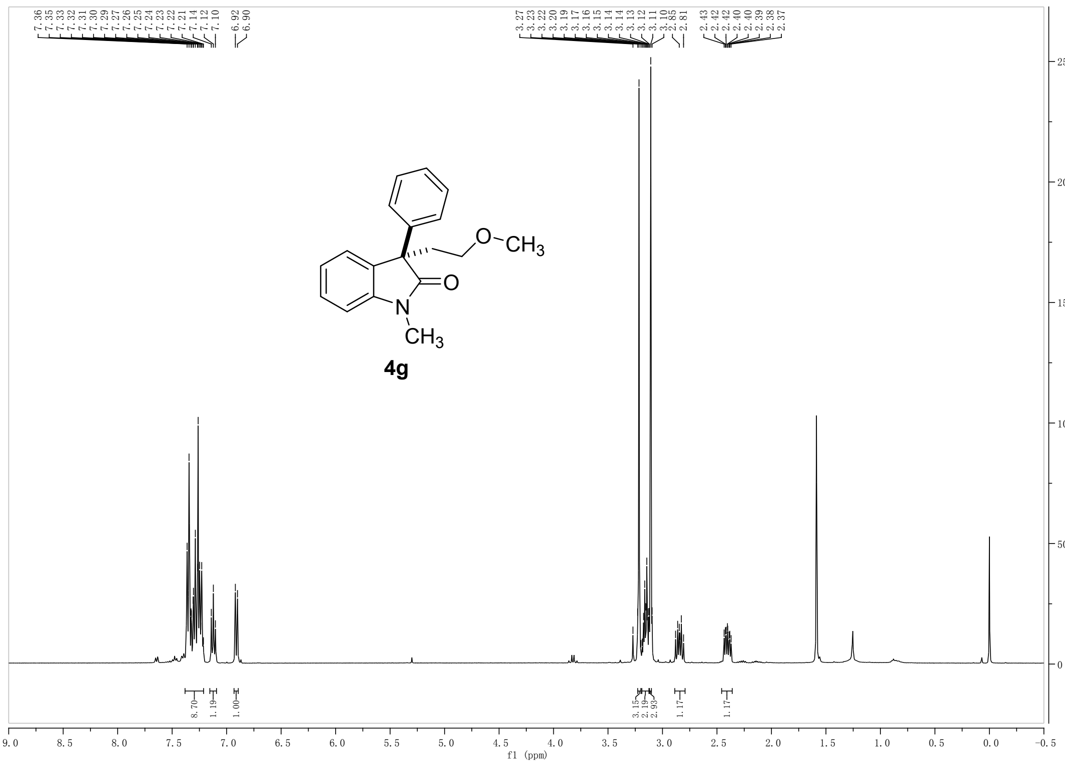

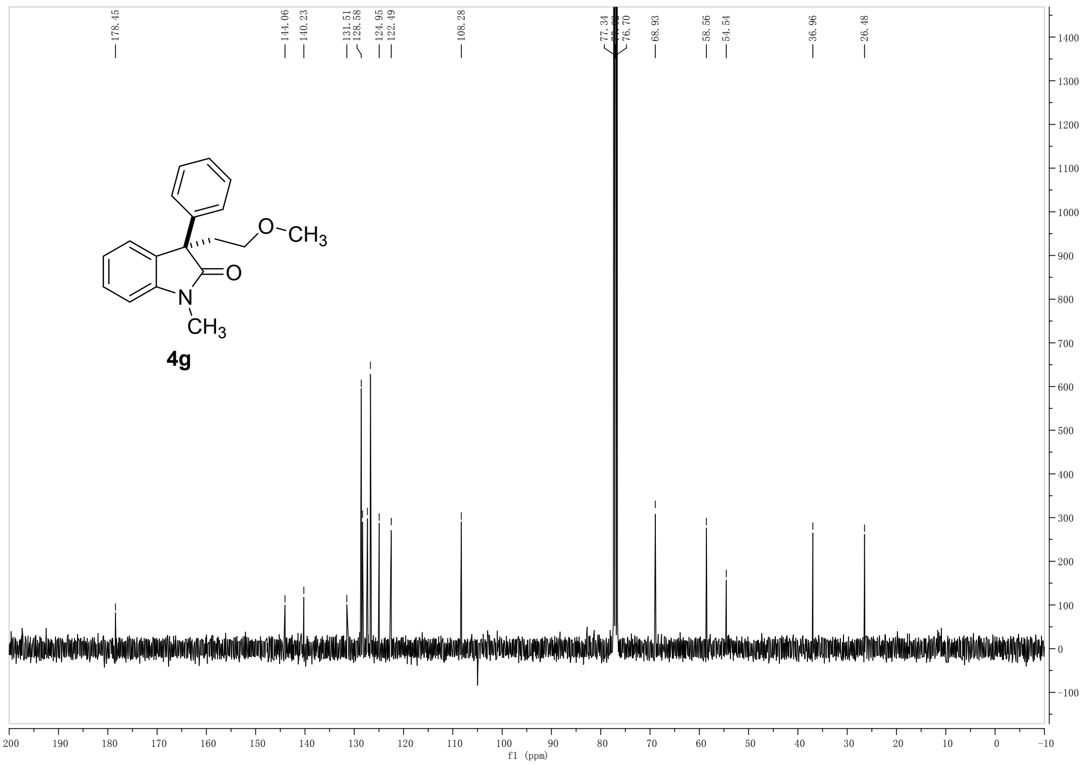

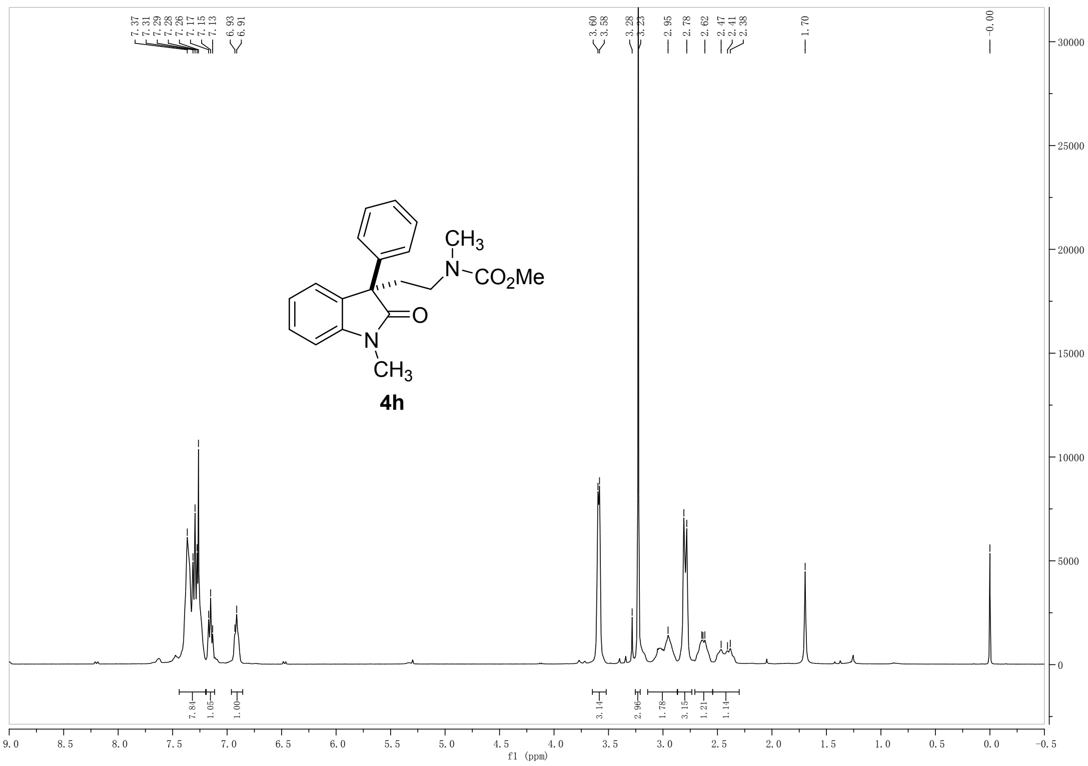
7.37 7.31 7.29 7.28 7.26 7.17 7.15 7.13 6.93 6.91
3.60 3.58 3.28 3.23 2.95 2.78 2.62 2.47 2.41 2.38 1.70 -0.00
CH3
N
CO2Me
O
N
CH3
4h
7.84 1.05 1.00 3.14 2.96 1.78 3.15 1.21 1.14
f1 (ppm)

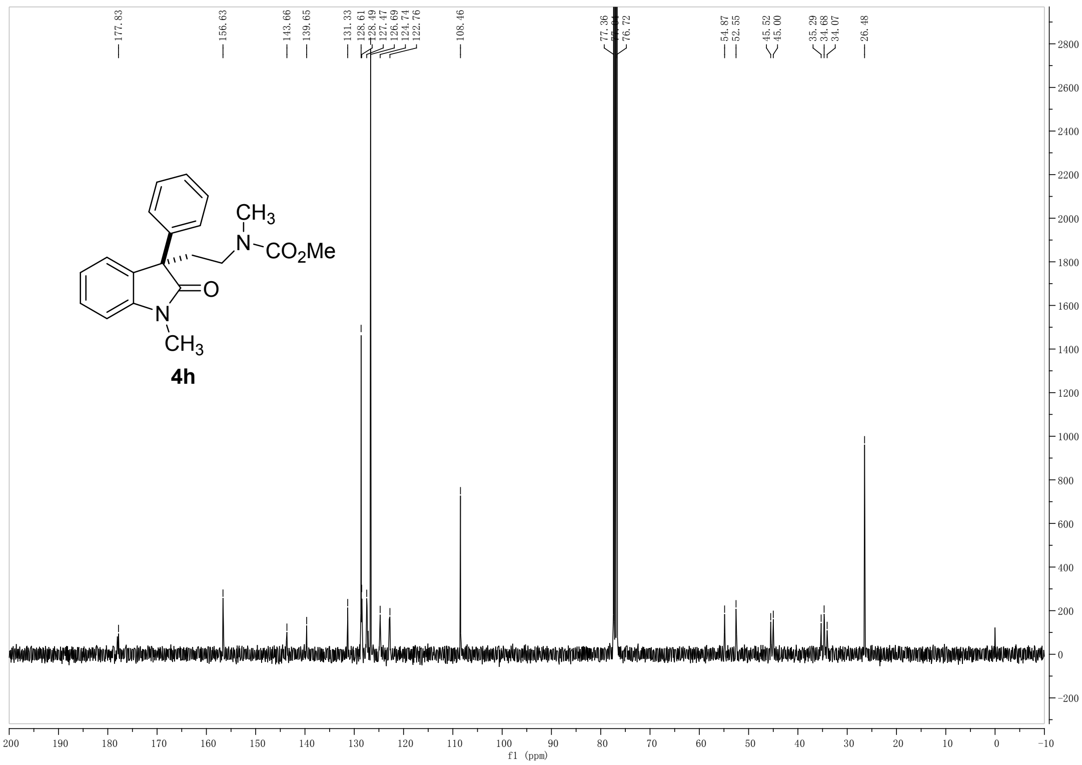
177.83 156.63 143.66 139.65 131.33 128.61 128.49 127.47 126.69 124.74 122.76 108.46
77.36 76.72
54.87 52.55 45.52 45.00 35.29 34.68 34.07 26.48
CH3
N
CO2Me
O
N
CH3
4h
f1 (ppm)

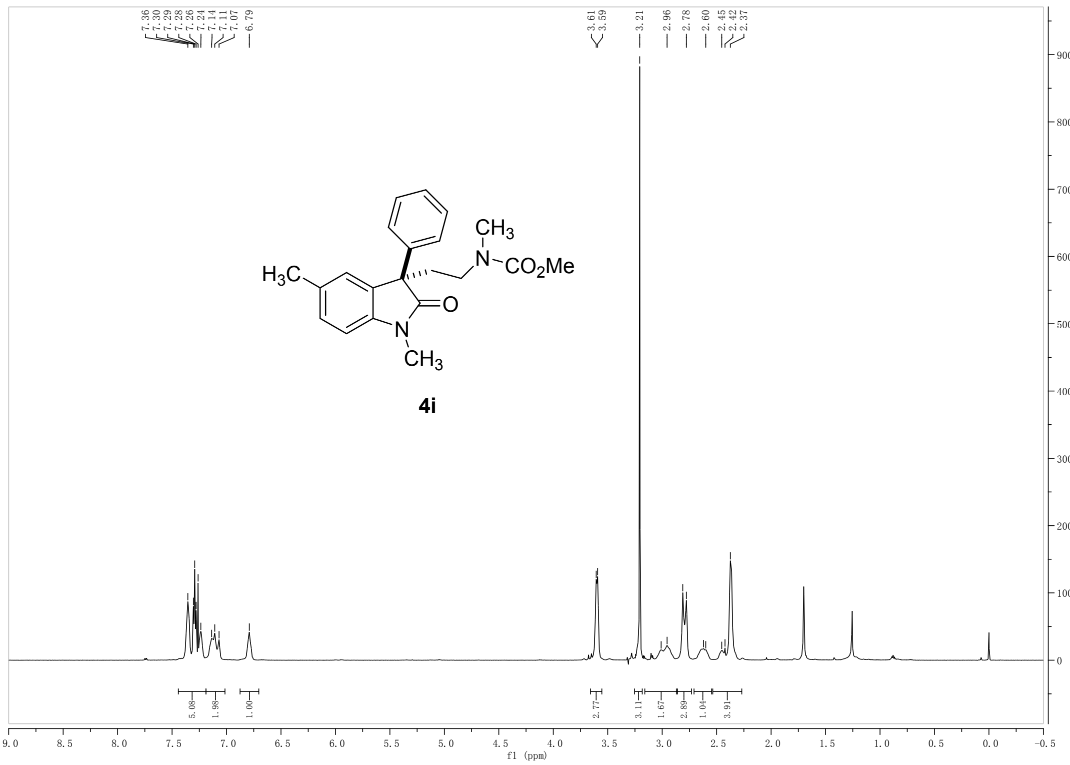
7.36
7.30
7.29
7.28
7.26
7.24
7.14
7.11
7.07
6.79
3.61
3.59
3.21
2.96
2.78
2.60
2.45
2.42
2.37
CH3
H3C
N
CO2Me
O
N
CH3
4i
5.08
1.98
1.00
2.77
3.11
1.67
2.89
1.04
3.91
f1 (ppm)

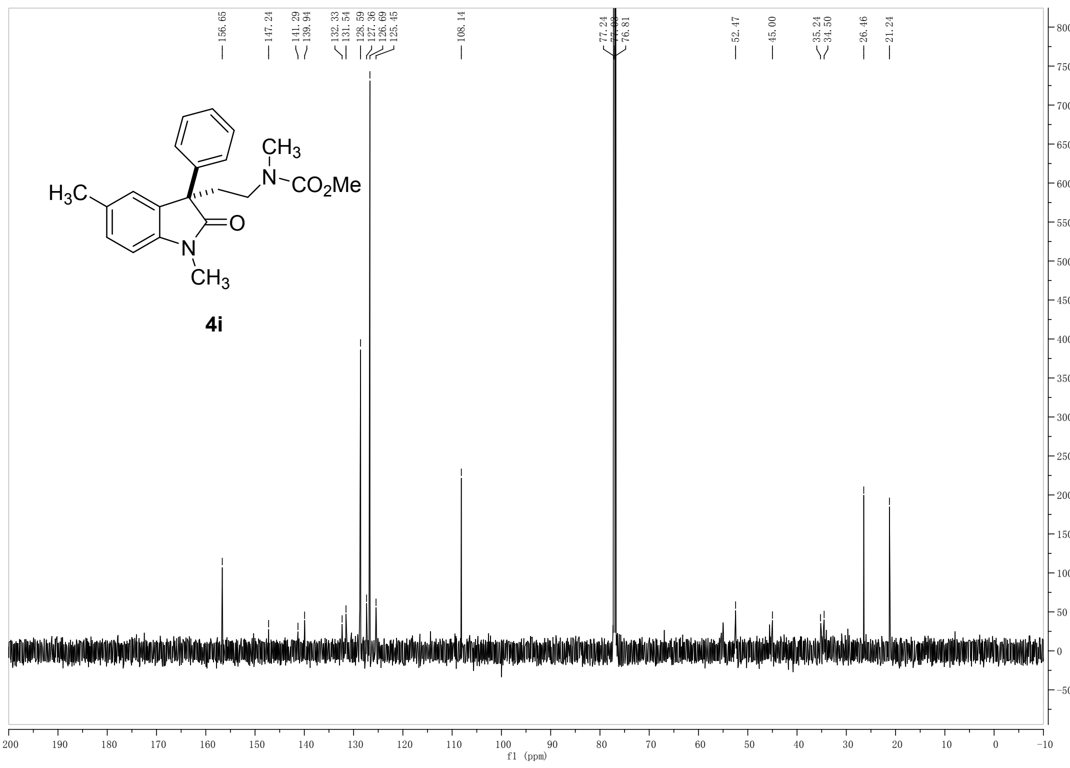
156.65
147.24
141.29
139.94
132.33
131.54
128.59
127.36
126.69
125.45
108.14
77.24
76.81
52.47
45.00
35.24
34.50
26.46
21.24
CH3
H3C
N
CO2Me
O
N
CH3
4i
f1 (ppm)

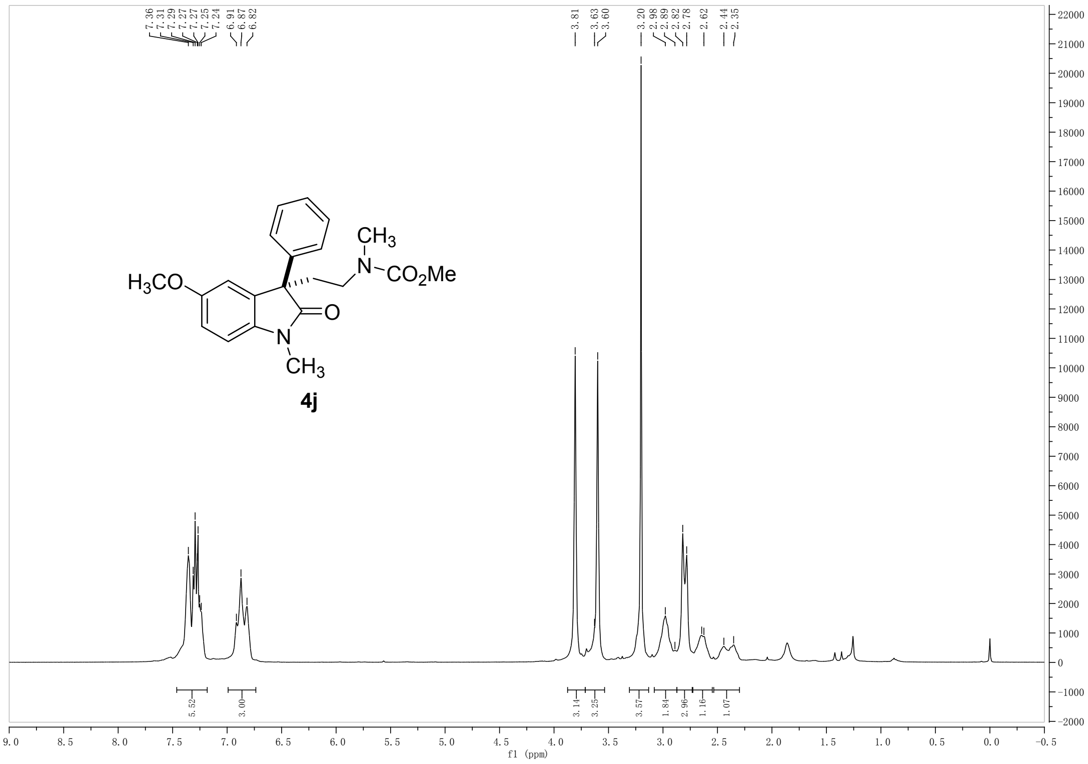

7.36
7.31
7.29
7.27
7.25
7.24
6.91
6.87
6.82
3.81
3.63
3.60
3.20
2.98
2.89
2.82
2.78
2.62
2.44
2.35
H3CO
CH3
N
CO2Me
O
N
CH3
4j
5.52
3.00
3.14
3.25
3.57
1.84
2.96
1.16
1.07
f1 (ppm)

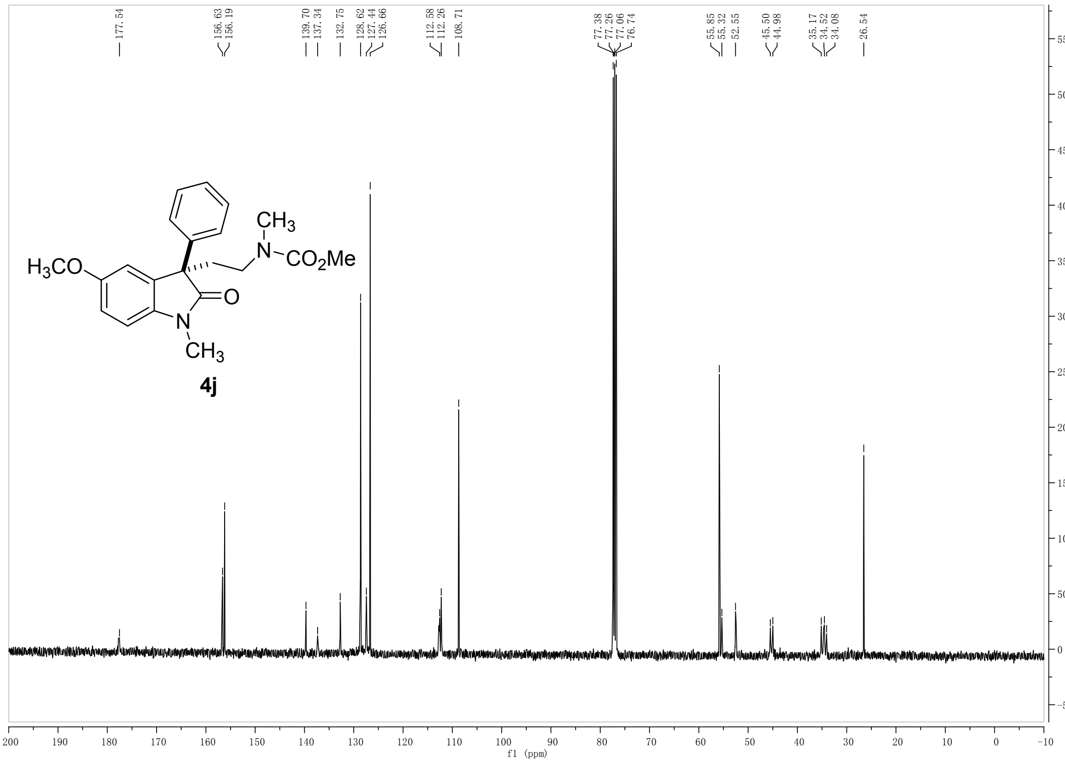

177.54
156.63
156.19
139.70
137.34
132.75
128.62
127.44
126.66
112.58
112.26
108.71
77.38
77.26
77.06
76.74
55.85
55.32
52.55
45.50
44.98
35.17
34.52
34.08
26.54
H3CO
CH3
N
CO2Me
O
N
CH3
4j
f1 (ppm)

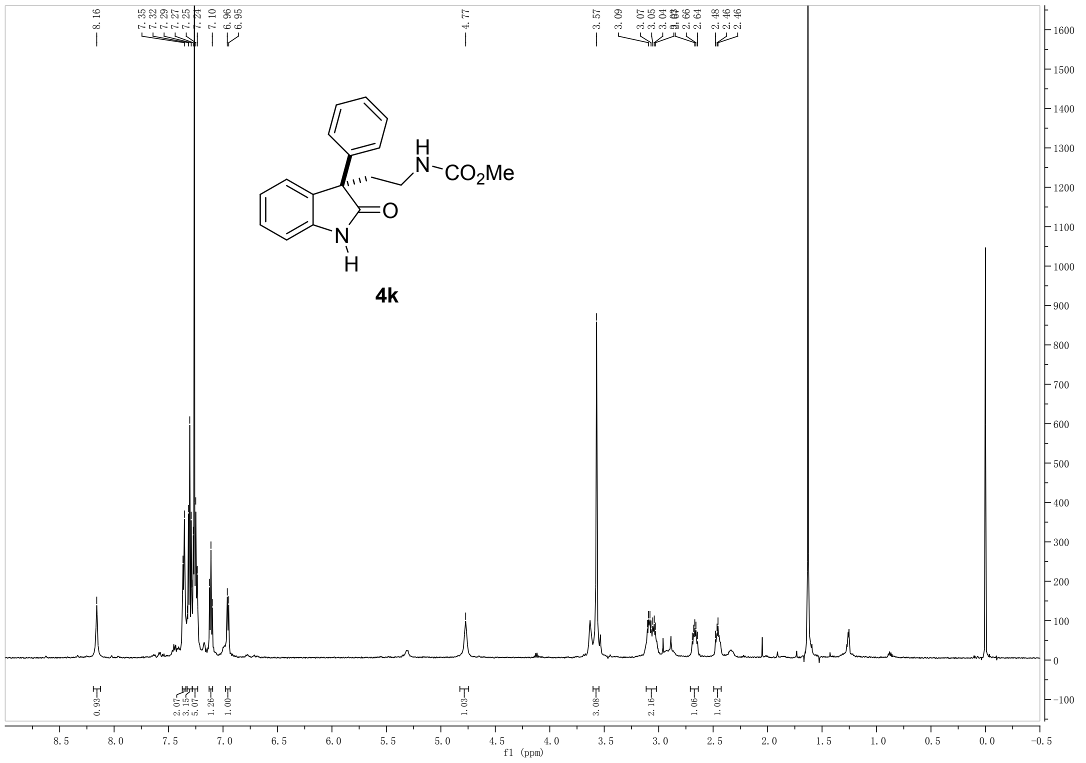

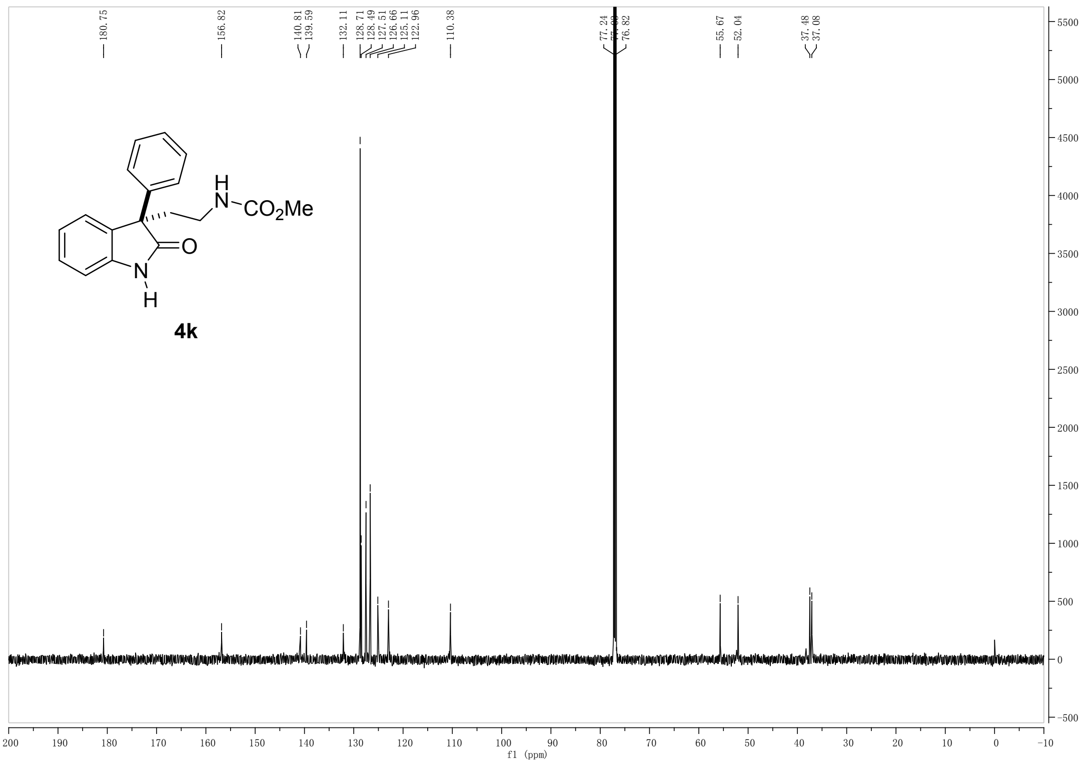

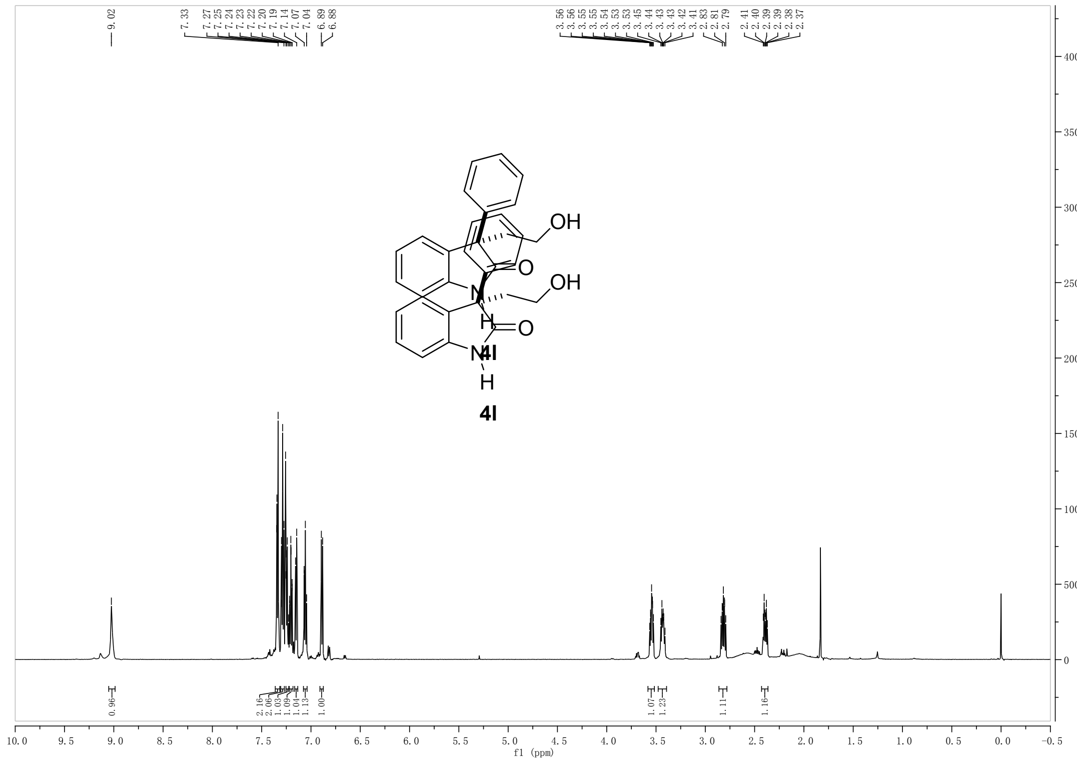
OH
O
OH
N
H
O
N
H
4l
f1 (ppm)

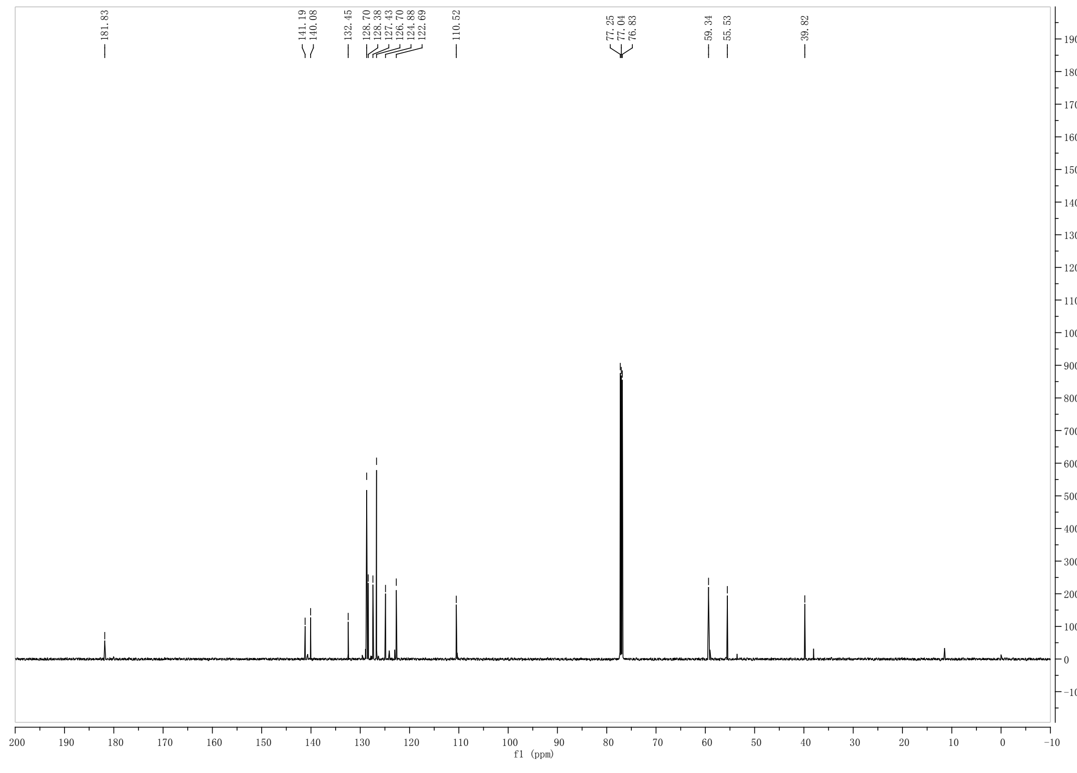
f1 (ppm)

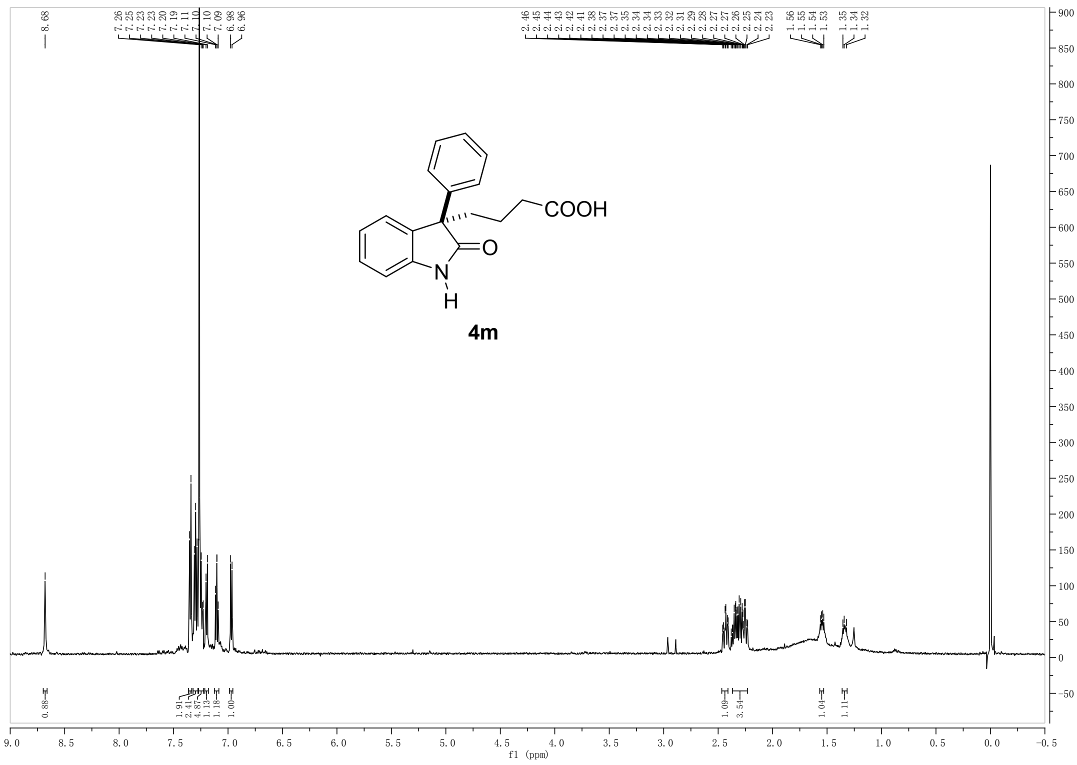
COOH
O
N
H
4m
f1 (ppm)

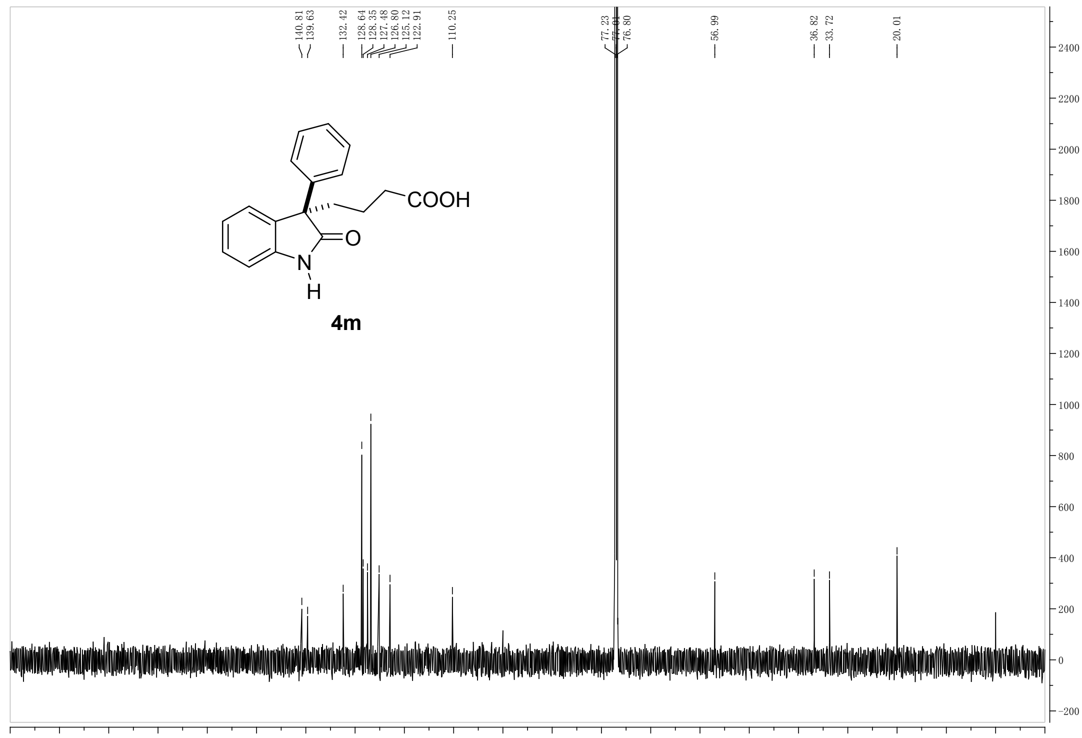
COOH
O
N
H
4m
f1 (ppm)

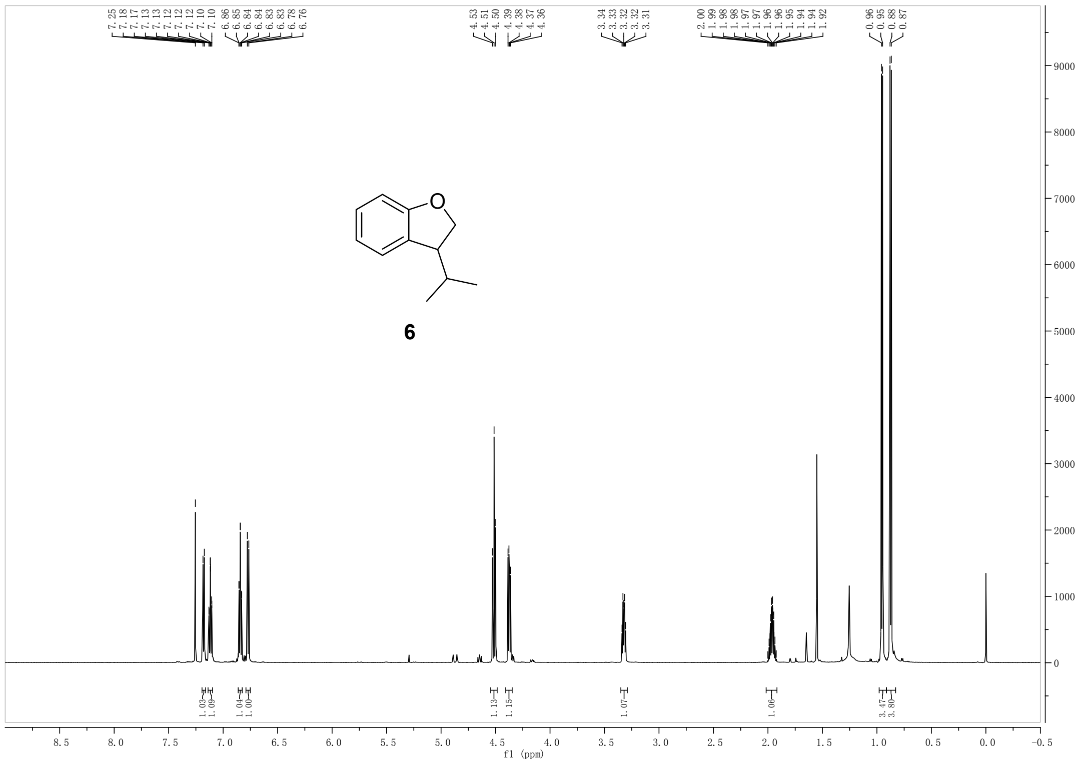

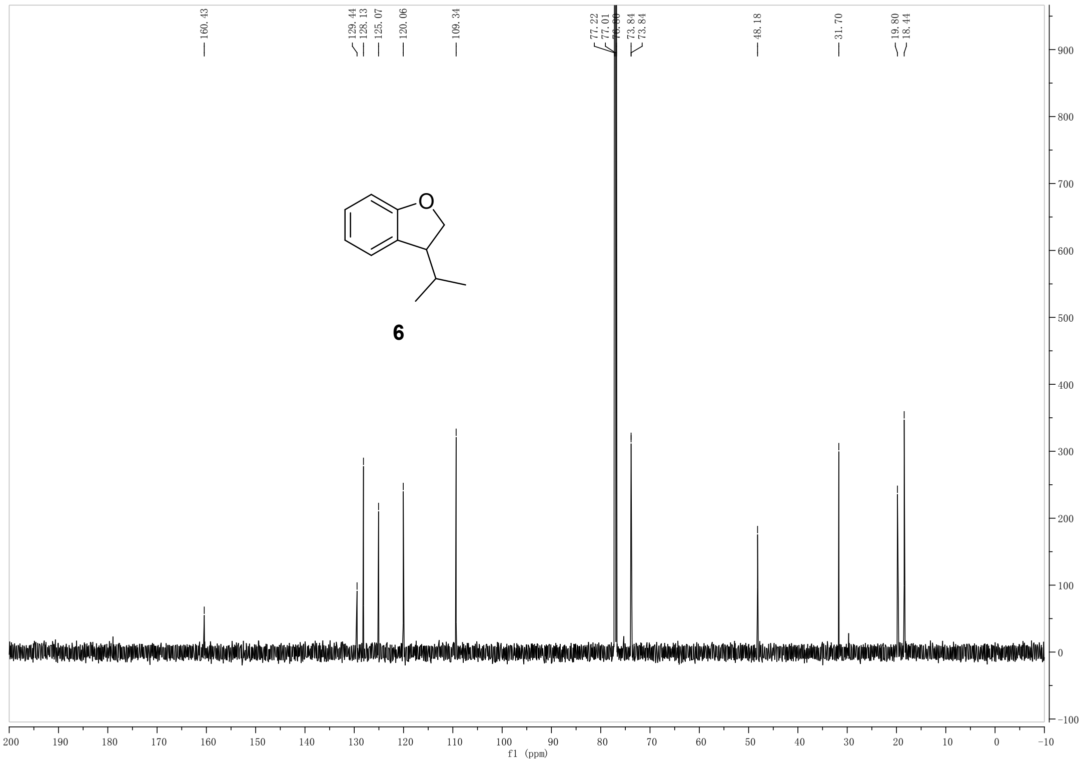

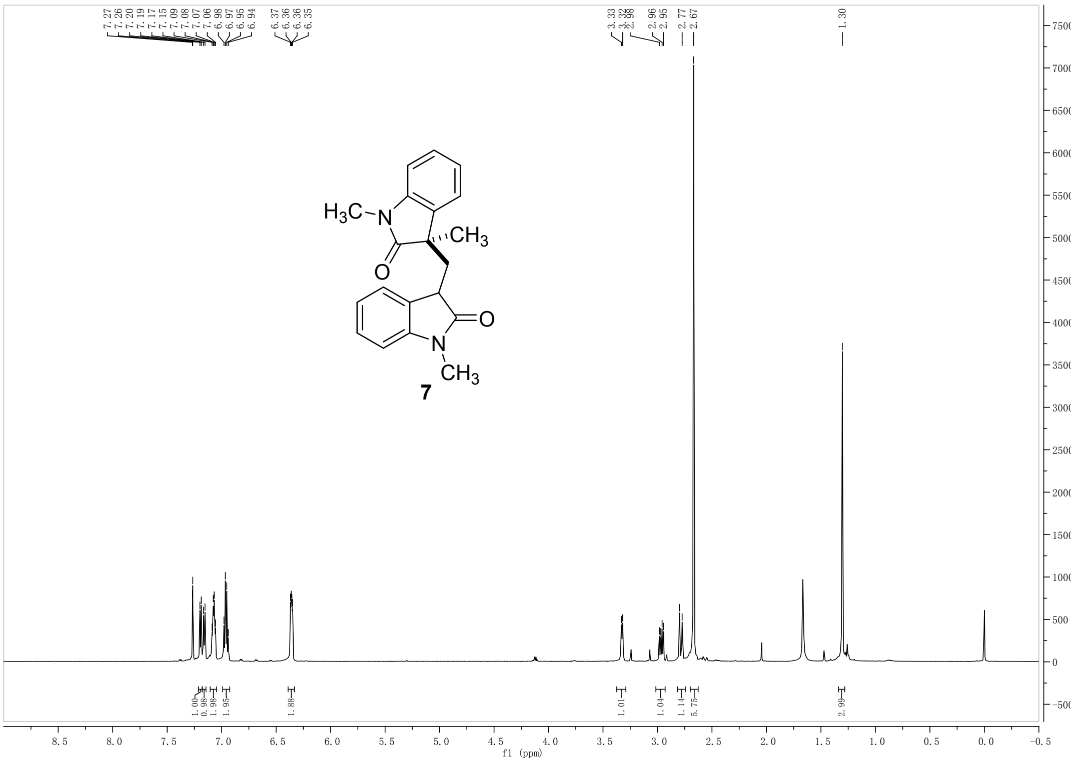
7.27
7.26
7.20
7.19
7.17
7.15
7.09
7.08
7.07
7.06
6.98
6.97
6.95
6.94
6.37
6.36
6.36
6.35
3.33
3.32
2.96
2.95
2.77
2.67
1.30
7
f1 (ppm)

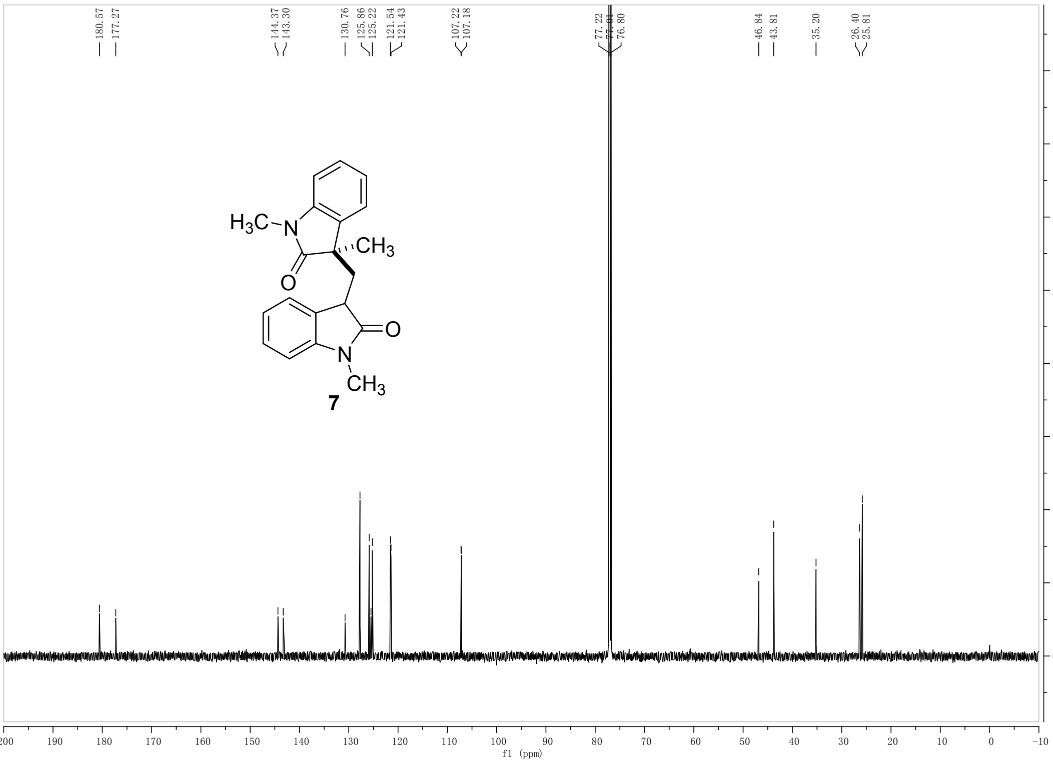
180.57
177.27
144.37
143.30
130.76
125.86
125.22
121.54
121.43
107.22
107.18
77.22
76.80
46.84
43.81
35.20
26.40
25.81
7
f1 (ppm)

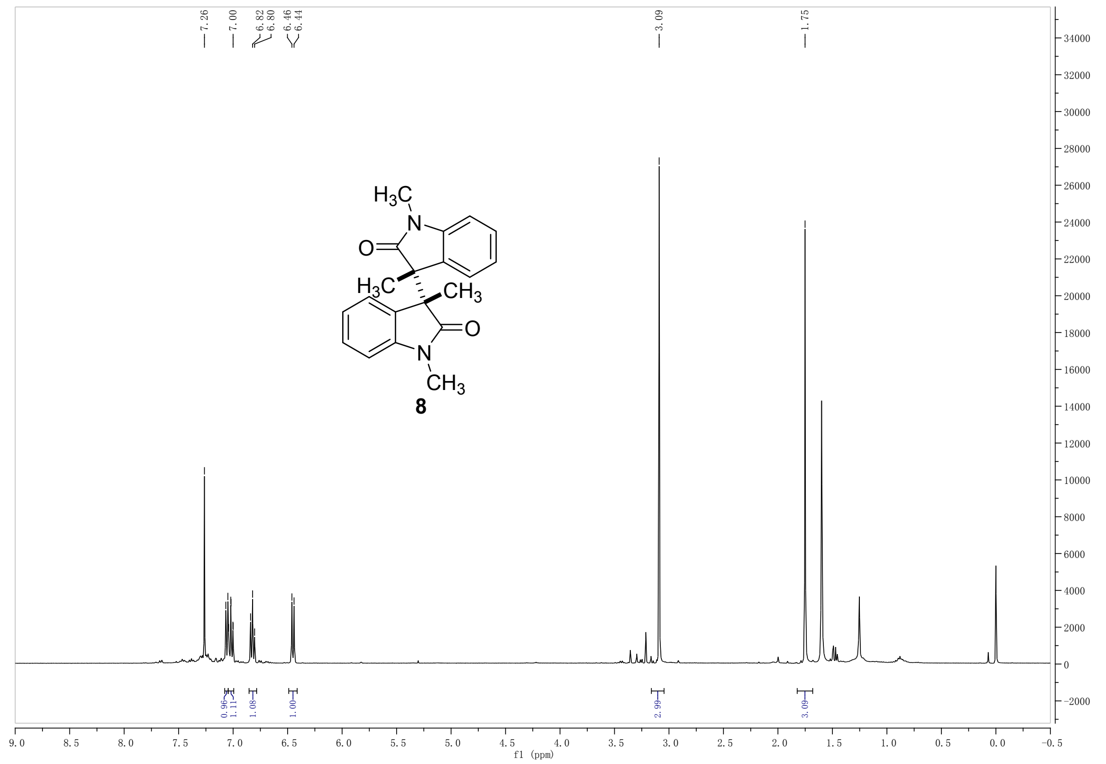
7.26
7.00
6.82
6.80
6.46
6.44
3.09
1.75
0.96
1.11
1.08
1.00
2.99
3.09
8
f1 (ppm)

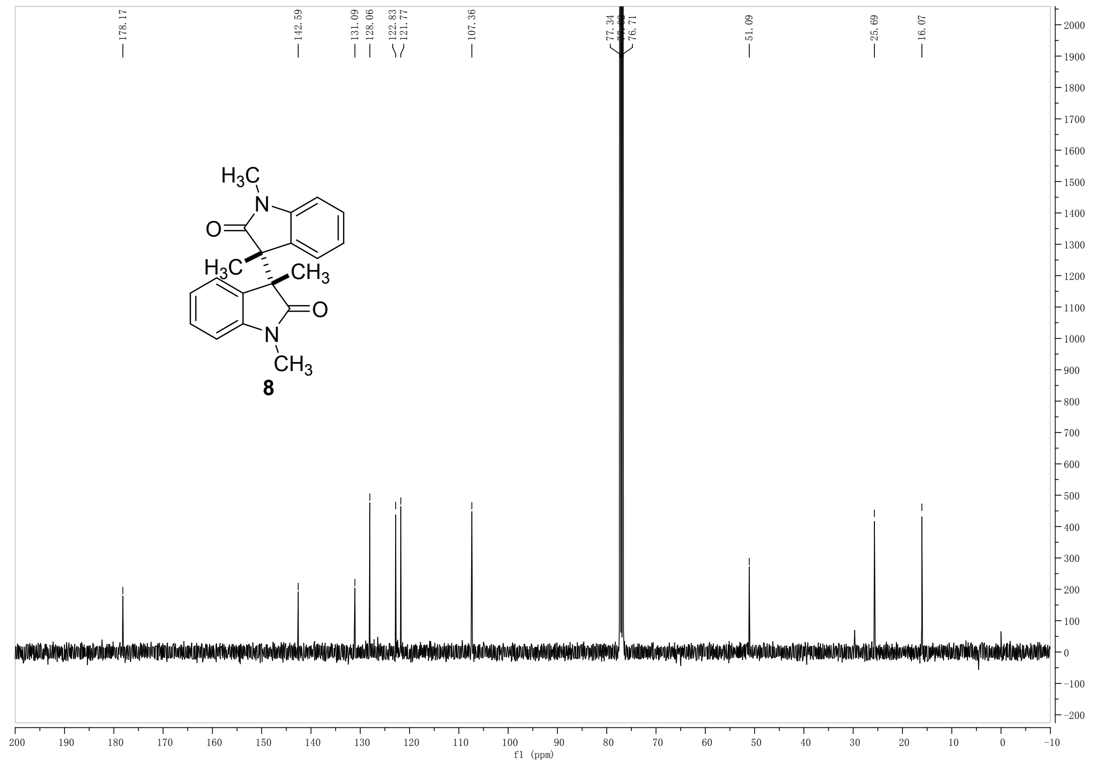
178.17
142.59
131.09
128.06
122.83
121.77
107.36
77.34
76.71
51.09
25.69
16.07
8
f1 (ppm)
